# Fingerprint of rice paddies in spatial–temporal dynamics of atmospheric methane concentration in monsoon Asia

**DOI:** 10.1038/s41467-019-14155-5

**Published:** 2020-01-28

**Authors:** Geli Zhang, Xiangming Xiao, Jinwei Dong, Fengfei Xin, Yao Zhang, Yuanwei Qin, Russell B. Doughty, Berrien Moore

**Affiliations:** 1 College of Land Science and Technology, China Agricultural University, Beijing, 100193 China; 2 Department of Microbiology and Plant Biology, University of Oklahoma, Norman, OK 73019 USA; 3 Key Laboratory of Land Surface Pattern and Simulation, Institute of Geographic Sciences and Natural Resources Research, Chinese Academy of Sciences, Beijing, 100101 China; 4 Ministry of Education Key Laboratory of Biodiversity Science and Ecological Engineering, Institute of Biodiversity Science, Fudan University, Shanghai, 200433 China; 5 Department of Earth and Environmental Engineering, Columbia University, New York, NY 10027 USA; 6 College of Atmospheric and Geographic Sciences, University of Oklahoma, Norman, OK 73019 USA

**Keywords:** Biogeochemistry, Agroecology, Climate-change ecology, Ecosystem ecology, Agriculture

## Abstract

Agriculture (e.g., rice paddies) has been considered one of the main emission sources responsible for the sudden rise of atmospheric methane concentration (XCH_4_) since 2007, but remains debated. Here we use satellite-based rice paddy and XCH_4_ data to investigate the spatial–temporal relationships between rice paddy area, rice plant growth, and XCH_4_ in monsoon Asia, which accounts for ~87% of the global rice area. We find strong spatial consistencies between rice paddy area and XCH_4_ and seasonal consistencies between rice plant growth and XCH_4_. Our results also show a decreasing trend in rice paddy area in monsoon Asia since 2007, which suggests that the change in rice paddy area could not be one of the major drivers for the renewed XCH_4_ growth, thus other sources and sinks should be further investigated. Our findings highlight the importance of satellite-based paddy rice datasets in understanding the spatial–temporal dynamics of XCH_4_ in monsoon Asia.

## Abstract (web summary)

The role of paddy rice agriculture in the spatial and temporal dynamics of atmospheric methane concentration remains unclear. Here, Zhang et al. show that regions with dense rice paddies have high satellite-based column averaged CH_4_ concentrations (XCH_4_), and that seasonal dynamics of XCH_4_ mirror those of paddy rice growth.

## Introduction

Atmospheric methane (CH_4_) concentration has increased substantially since early 2007, after a hiatus during 1999–2006^1–5^; however, there is no consensus on the possible causes for this observed increase^1–11^. Recent studies suggested that biogenic sources may have contributed most to the ongoing increase of CH_4_ emission^4,5,12^, especially the expansion of tropical agriculture^4,5^. Rice paddies are an important biogenic and agricultural source of CH_4_ emission and have thus attracted renewed attention^4,5,12,13^, especially in monsoon Asia with 87% of the global paddy rice harvested area and 90% of the rice production (according to FAOSTAT in 2017), and ~25–36% of global CH_4_ emissions^13,14^. Rice paddy CH_4_ emissions are predicted to increase by the end of the 21st century due to the enhancement of rice plant productivity from a warmer climate and atmospheric carbon fertilization^15^, and increased rice paddy area driven by an increasing market demand for rice as a food staple^16^. Hence, it is critical to understand the role of rice paddies in the observed spatial distribution, seasonal dynamics, and interannual variation of atmospheric CH_4_ concentration.

Advanced satellite measurements of column-averaged concentration of CH_4_ (XCH_4_) provide large-scale constraints for CH_4_ emission estimates^17–19^ and are used to detect surface CH_4_ emission hotspots from space^20,21^. The Scanning Imaging Absorption Spectrometer for Atmospheric Chartography (SCIAMACHY) obtained from short-wavelength infrared (SWIR) sensors can measure atmospheric CH_4_ concentration from a lower altitude down to the surface, which are more indicative of the emissions from the ground^17,18^. SCIAMACHY allows us to uncover and identify the relationships between major sources of surface CH_4_ emissions and atmospheric CH_4_ columns. Satellite data sets for methane production-related variables (e.g., spatial distribution of methane emission sources) could be useful to associate XCH_4_ observations to emission sources^22^. Bloom et al.^18^ analyzed the roles of wetlands and rice paddies in determining the temporal dynamics and spatial distribution of atmospheric CH_4_ concentration based on the correlations between XCH_4_ data from SCIAMACHY and water table depth from the Gravity Recovery and Climate Experiment (GRACE) satellite. They found that the observed seasonal variability of XCH_4_ matched closely with the fluctuations in wetlands and rice CH_4_ emissions over wetland regions, thus explaining 70% of the methane emissions from surface sources. However, the relative contributions of natural wetlands and rice paddies to the seasonal dynamics and spatial distribution of atmospheric CH_4_ concentration in different countries cannot be well quantified using coarse-resolution paddy rice maps (1° × 1°)^18,23^ and water table depth data from GRACE (3° × 3°)^18^. Hayashida et al.^17^ combined XCH_4_ data from SCIAMACHY with satellite-derived land surface water coverage (LSWC) and normalized difference vegetation index (NDVI) to quantify the contributions of rice cultivation to the spatial distribution and seasonality of XCH_4_ over rice paddy areas derived from agricultural statistics in monsoon Asia. Nevertheless, the relationship between rice cultivation and atmospheric CH_4_ concentration in rice paddy regions based on the statistical data of rice harvest areas cannot accurately characterize the role of rice paddies in the seasonal fluctuations of XCH_4_.

Although numerous measurements and analyses of CH_4_ emission from rice paddies at the site scale have been done^24–27^, the influence of rice paddies on the spatial distribution and seasonal dynamics of atmospheric CH_4_ concentration is still poorly understood at the continental scale, in part due to the lack of moderate to high-spatial resolution maps of paddy rice croplands. The accurate spatial and temporal pattern of rice paddies is critical for understanding the contribution of rice paddies to atmospheric CH_4_ concentration.

Here, we first generate annual maps of rice paddies in continental monsoon Asia at 500 -m spatial resolution over the period of 2000–2015 through analyses of time series images from Moderate Resolution Imaging Spectroradiometer (MODIS) sensor, using the robust paddy rice mapping algorithms, which were well documented in our previous studies^28–31^. The resultant annual maps of rice paddies show the spatial–temporal changes of rice paddy areas in monsoon Asia during 2000–2015, including the hot spots and interannual trends. Second, we use the annual MODIS-based paddy rice maps during 2000–2015, MODIS-based vegetation indices, and the XCH_4_ data from SCIAMACHY on the Environmental Satellite (ENVISAT) and the Thermal and Near Infrared Sensor for Carbon Observation Fourier-Transform Spectrometer (TANSO-FTS) onboard the Greenhouse Gases Observing Satellite (GOSAT), to quantify the role of rice paddies in determining the spatial distribution and seasonal dynamics of atmospheric CH_4_ concentration in monsoon Asia. The results show that geographically those regions with relatively larger proportion of rice paddies have high XCH_4_. In those areas dominated by single- or double-paddy rice cropping systems, the seasonal dynamics of XCH_4_ also has one or two peaks in a year, corresponding well with the seasonal dynamics of paddy rice growth. Third, we assess the interannual dynamics of rice paddy area and XCH_4_ in monsoon Asia. The results show a decreasing trend of rice paddy area and a renewed growth of XCH_4_ in monsoon Asia since 2007. Implications of this study include both a broader perspective regarding satellite-based annual maps of rice paddies at moderate-to-high spatial resolutions and the potential and challenges in understanding the spatial–temporal dynamics of XCH_4_ in monsoon Asia.

## Results and discussion

### Dynamics of rice paddies in monsoon Asia during 2000–2015

**Fig. 1:**
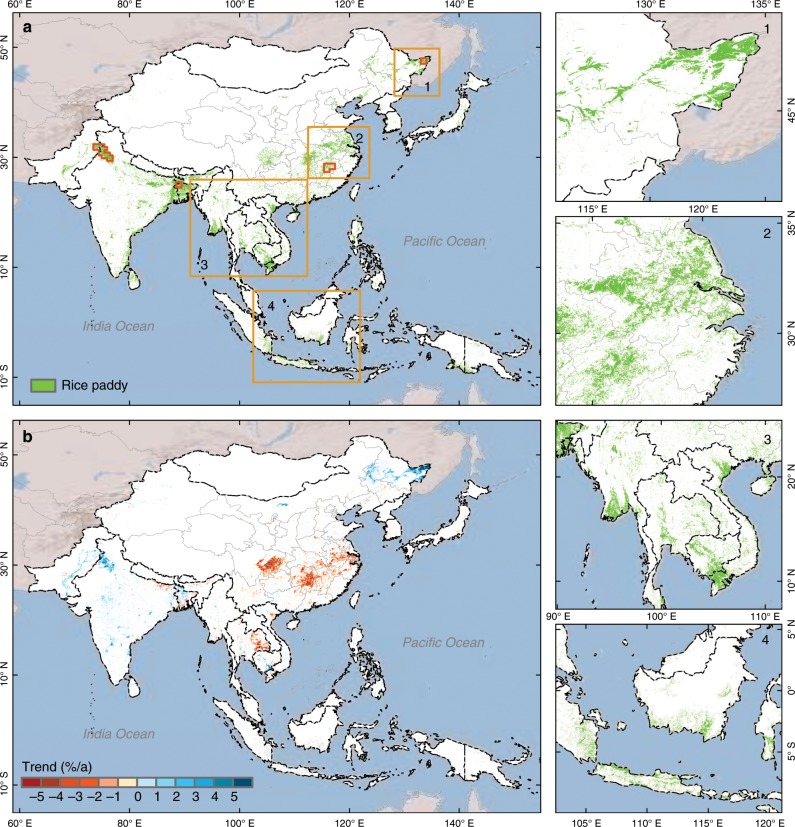
Spatial distributions of paddy rice croplands and its trend from 2000 to 2015 in monsoon Asia. **a** The paddy rice map was retrieved from MODIS data with 500 -m resolution in monsoon Asia in 2015. **b** The spatial pattern of trends in rice paddy area at 5 × 5 km gridcells during 2000–2015. **1**–**4** Detailed spatial distributions of rice paddies in local regions, labeled in **a** with orange rectangles. The small and red polygons in **a** are the samples illustrated in Fig. 3. Source data are provided as a Source Data file.

We generated the annual paddy rice maps during 2000–2015 and quantified the spatial–temporal changes in rice paddy area in monsoon Asia. Figure 1a shows the spatial pattern of rice paddies in 2015 over monsoon Asia at 500 -m spatial resolution. China and India had the largest total area of rice paddies, and together accounted for over half the total rice paddy area in monsoon Asia (Supplementary Fig. 1). Rice paddies were mainly located in the alluvial plains of major rivers in this region, including the Indo-Gangetic Plain in eastern India, Yangtze Plain in southern China, Ayeyarwady Delta in southern Myanmar, and Mekong Basin in Southeast Asia. The rice paddies in monsoon Asia substantially increased from 2000 to 2007, but then decreased from 2007 to 2015 (Supplementary Fig. 2). Geographically, those regions with significant decreasing trends in rice paddy area during 2000–2015 included the Yangtze Plain of southern China and eastern Thailand, while Northeast China and India had significantly increasing trends in rice paddy area (Fig. 1b). These annual maps provide improved data and knowledge of the spatial distribution and interannual variation of rice paddy in monsoon Asia.

### Spatial consistency between rice paddies and XCH_4_

**Fig. 2:**
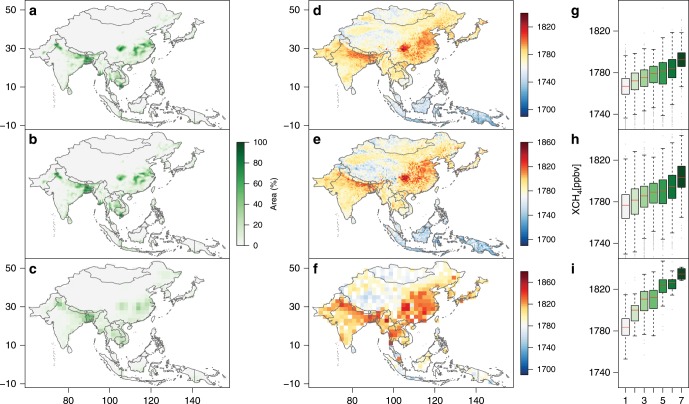
Consistency of spatial distributions between paddy rice croplands and atmospheric methane concentration. The three periods (2003–2005, 2007–2009, and 2011–2013) were selected in the main text to illustrate the spatial relationships between rice paddies and XCH_4_. **a**–**c** Spatial distributions of 3-year averaged area proportions of MODIS-based paddy rice croplands for 2003–2005 with 0.5° gridcells (**a**), 2007–2009 with 0.5° gridcells (**b**), and 2011–2013 with 2.0° gridcells (**c**). **d**–**f** Spatial distributions of 3-year averaged annual column-averaged concentration of CH_4_ (XCH_4_) from SCIAMACHY for 2003–2005 with 0.5° resolution (**d**), 2007–2009 with 0.5° resolution (**e**), and from TANSO-FTS for 2011–2013 with 2.0° resolution (**f**). **g**–**i** 3-year averaged annual XCH_4_ for different rice paddy area proportions for 2003–2005 (**g**), 2007–2009 (**h**), and 2011–2013 (**i**). The *x*-axis values in figures (**g**–**i**) represent levels of rice paddy area proportions in monsoon Asia, and 1–7 correspond to <0.5%, 0.5–1%, 1–5%, 5–10%, 10–20%, 20–40%, and >40% rice paddy area proportions, respectively. Source data are provided as a Source Data file.

We investigated the relationships between the MODIS-based paddy rice maps and satellite observed XCH_4_ in monsoon Asia at various spatial and temporal scales. The spatial distributions of rice paddies were consistent with those of atmospheric CH_4_ concentration over six 3-year moving-window periods (2003–2005, 2005–2007, 2007–2009, 2009–2011, 2011–2013, and 2013–2015, Fig. 2a–f; Supplementary Fig. 3a–f). Those regions with high densities of rice paddies also had high XCH_4_. For example, the high densities of rice paddies in Indo-Gangetic Plain in north India, Bangladesh, and the Yangtze Plain and Sichuan Basin in China matched closely with those areas with high XCH_4_ (Fig. 2a–f; Supplementary Fig. 3a–f).

Different densities (% percentage) of rice paddies within individual gridcells over each of the 3-year periods were divided into seven intervals (a gradient), and the XCH_4_ within each interval were analyzed. The 3-year mean XCH_4_ increased as the density of rice paddies rose for all these 3-year periods (Fig. 2g–i; Supplementary Fig. 3g–i). Similar relationships were also found in different seasons in these periods (Supplementary Figs. 4–7). We used the spatial error model (SEM) to quantify their spatial relationships during the six 3-year periods, and the results showed that the density of rice paddies had high spatial correlation with the 3-year mean XCH_4_ (*P*-value of coefficients < 0.001, and *P*-value of Moran’s I test of residuals > 0.3) for all these periods (Supplementary Table 1). It should be noted that clouds and shadows in the inter-tropical convergence zone (e.g., Indonesia and Malaysia) occur frequently (Supplementary Fig. 8), which could affect satellite-based optical images used for paddy rice mapping and XCH_4_ retrieval. In those areas with frequent cloud cover and shadows, there is potentially a multicollinearity issue in the rice paddy area and XCH_4_ data. We re-ran the SEM analysis for the other monsoon Asia countries after excluding Indonesia and Malaysia, and the results still showed strong spatial consistency between rice paddy area and XCH_4_ (Supplementary Figs. 9–12 and Supplementary Table 2). These results suggested that the spatial distribution of rice paddies was one of the major factors that determine the spatial distributions of atmospheric CH_4_ concentration in monsoon Asia.

### Seasonal consistency of paddy rice growth and XCH_4_

Enhanced vegetation index (EVI) is related to vegetation canopy^32^ (Supplementary Fig. 13), and has been used to estimate gross primary production of paddy rice^33^. We used MODIS-based EVI as a proxy for rice plant growth to quantify the seasonal relationship between the paddy rice growth and atmospheric CH_4_ concentration in a year. We analyzed time series EVI and XCH_4_ data at selected regions of interest (ROIs) with different rice cropping systems and rice paddy-dominated regions in monsoon Asia.

**Fig. 3:**
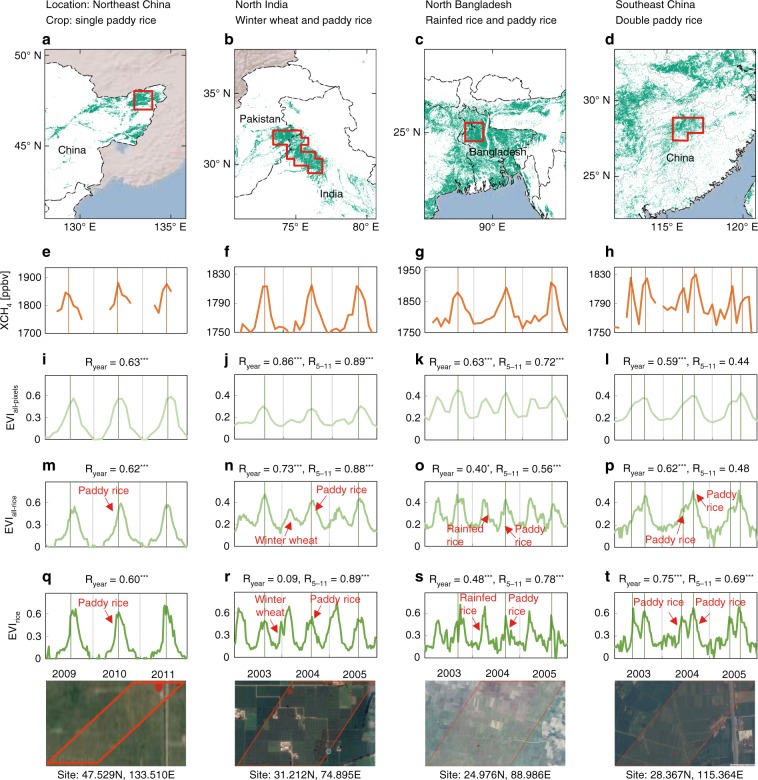
Seasonal dynamics of atmospheric methane concentration and rice growth. The column-averaged methane concentration (XCH_4_) and the enhanced vegetation index (EVI) were analyzed in four regions of interest (ROIs) with dense rice paddies and different cropping systems. **a**–**d** Spatial distributions of rice paddies retrieved from MODIS data with 500 -m resolution in the Sanjiang Plain of Northeast China in 2010 with single cropping paddy rice (**a**), North India in 2005 with winter wheat and paddy rice (**b**), North Bangladesh in 2005 with rainfed rice and paddy rice (**c**), and Poyang Lake of China in 2005 with early and late paddy rice (**d**). The four ROIs are shown in Fig. 1a with small and red polygons. **e**–**h** Time series of monthly SCIAMACHY CH_4_ column volume mixing ratios (VMRs, in parts per billion) over the corresponding four ROIs labeled in (**a**–**d**) with red polygons. **i**–**l** Time series of monthly MODIS-based EVI for all pixels within 0.5° gridcells (EVI_all-pixels_) over the aforementioned four ROIs. **m**–**p** Time series of 8-day EVI values for all 500 m rice paddy pixels within the 0.5° gridcells (EVI_all-rice_). **q**–**t** Time series of 8-day EVI values for one rice paddy pixel with 500 m resolution (EVI_rice_). The four bottom images are the corresponding landscapes for each rice paddy pixel (**q**–**t**) with the extent of 500 m by 500 m from Google Earth. R_year_ and R_5–11_ offer the Pearson’s correlation coefficients between XCH_4_ and EVI for the whole year and summer-fall season from May to November during the corresponding period labeled in the *x*-axis of (**q**–**t**). ***P < 0.01; *P < 0.10. Correlation coefficients without asterisk are insignificant (*P* > 0.10). Source data are provided as a Source Data file.

First, we analyzed the seasonal dynamics of XCH_4_ and paddy rice growth in four typical ROIs with a high density of rice paddies and different cropping systems (single- and double-cropping systems) (Fig. 3). We calculated mean EVI for all pixels within 0.5° gridcells (EVI_all-pixels_), mean EVI for all rice paddy pixels within 0.5° gridcells (EVI_all-rice_), and EVI for one rice paddy pixel with 500 -m resolution (EVI_rice_). The seasonal dynamics of XCH_4_ had one peak per year in three regions (Northeast China, northern India, and northern Bangladesh, Fig. 3e–g), and two peaks per year in one region (southern China, Fig. 3h). The consistency in the peak timings of XCH_4_ and EVI clearly increased from EVI_all-pixels_ to EVI_all-rice_ and EVI_rice_, suggesting the contribution of rice growth to XCH_4_. The Sanjiang Plain of Northeast China (Fig. 3a) had a single crop per year, mostly paddy rice. Remarkably, both XCH_4_ (Fig. 3e) and EVI (Fig. 3i, m, q) had a single peak around August in each year, and the seasonal dynamics of XCH_4_ corresponded well with those of the three EVI data sets (all of Pearson’s correlation coefficients between EVI and XCH_4_ during the entire year R_year_ ≥ 0.60, *P* < 0.001, Fig. 3i, m, q). Northern India and northern Bangladesh (Fig. 3b, c) had a double-cropping rotation with winter wheat/rainfed rice and paddy rice in each year according to previous studies^34,35^. The seasonality of XCH_4_ (Fig. 3f, g) had only one peak, which corresponded well with the seasonality of paddy rice (the second crop in these regions) in the EVI_all-pixel_, EVI_all-rice_, and EVI_rice_ data (Fig. 3 j, k, n, o, r, s, all of the Pearson’s correlation coefficients between EVI and XCH_4_ during the summer-fall season from May to November R_5–11_ larger than R_year_). The Poyang Lake region of southern China (Fig. 3d) had a mixture of single rice and rice–rice double-cropping rotations in one year^36^. The annual XCH_4_ (Fig. 3h) in this region had two distinct seasonal cycles, which corresponded well with the seasonal dynamics of EVI_rice_ (Fig. 3t). It should be noted that there were no obvious double peaks for EVI_all-pixel_ and EVI_all-rice_ in one year at a coarse spatial gridcell resolution of 0.5° resolution (Fig. 3l, p) due to the mixture of single and double rice cropping rotations in this region. These results suggested that the growth cycle of paddy rice contributed significantly to the seasonality of XCH_4_.

It is well recognized that rice plants play a critical role in the processes of methane production^37,38^. Several recent in situ studies found a strong correlation between daily CH_4_ flux and rice plant biomass, which suggested that methane flux in rice paddies is affected by rice growth and development^37–44^. Here, we compared the observed CH_4_ emission and EVI at eight paddy rice sites^25–27,41,42,45–47^, and the results confirmed their consistency in terms of seasonality (Supplementary Fig. 14). The synchrony of rice growth and CH_4_ emissions is due to the fact that the rhizodeposition from current season photosynthesis and plant growth controlled the organic matter of the flooded soils, and subsequently determined the methanogenesis in paddy soils^38,48,49^. Rice plants provide root exudates, which is the important organic matter used by soil microbes for CH_4_ production in rice paddies^37,44^. The quantity of root exudates varies during the rice plant growing season, which rises as the rice plants grow, reaches a maximum during the flowering stage with a peak in root biomass, and then decreases (Supplementary Fig. 13, Supplementary Note 1)^38^.

Although this synchronized peak has been previously found at field site, our results using satellite data show synchrony at large scales for the first time with the aid of high-resolution paddy rice maps. Moreover, the rice cropping systems affected the peaks in XCH_4_, that is, the XCH_4_ variation was controlled by the paddy rice growth cycle regardless of whether it was a single- or double-cropping system (wheat–rice, rice–rice) (Fig. 3). The ROI-scale analyses suggested the modeling of the seasonal dynamics of atmospheric CH_4_ concentration need to consider the rice cropping system (single rice, double rice, or rice plus other crop) rather than general cropping intensity (single, double, or multiple cropping).

**Fig. 4:**
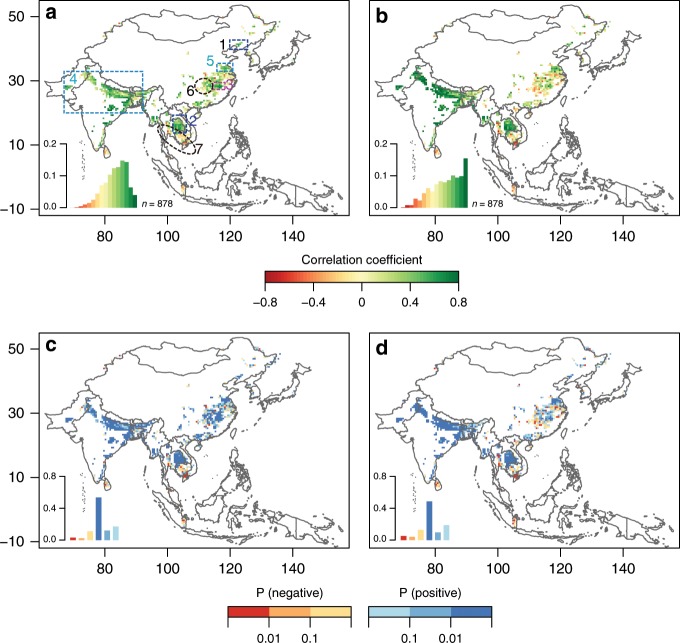
Spatial patterns of correlations between seasonal atmospheric methane concentration and rice growth. The monthly atmospheric column-averaged methane concentration (XCH_4_) from SCIAMACHY and the enhanced vegetation index (EVI) from MODIS were analyzed in the dense rice paddy areas of monsoon Asia during 2003–2005. **a**, **b** The spatial distributions of Pearson’s correlation coefficients between XCH_4_ and EVI for the whole year and summer-fall season from May to November, respectively. **c**, **d** The spatial distributions of significance levels of Pearson’s correlation between the two for the whole year and summer-fall season from May to November, respectively. The region with a 3-year averaged rice paddy gridcells with area proportion larger than 10% during 2003–2005 is considered here. The insets in (**a**–**d**) are the corresponding frequency diagrams of Figure (**a**–**d**). Source data are provided as a Source Data file.

Second, we further analyzed the spatial patterns of seasonal consistency between paddy rice growth and XCH_4_ in monsoon Asia using monthly EVI_all-rice_ and XCH_4_ at 0.5° gridcells during 2003–2005 (Fig. 4). Figure 4a, b showed the spatial patterns of the Pearson’s correlation coefficients between EVI and XCH_4_ during the entire year (R_year_) and the summer-fall season from May to November (R_5–11_) during 2003–2005, respectively. Regions with the same or similar cropping system and planting schedule demonstrated clear relationships between paddy rice growth and XCH_4_. Most 0.5° gridcells in regions dominated by a single rice crop in a year had statistically significant and positive Pearson’s correlation coefficients between EVI_all-rice_ and XCH_4_ at both yearly and seasonal scales (R_year_ and R_5–11_), as seen in the Liaohe Plain in China^31^ (Region 1 in Fig. 4a) and eastern Thailand^50,51^ (Region 2 in Fig. 4a, Supplementary Fig. 15). Most 0.5° gridcells in regions dominated by double rice cropping systems (rice–rice rotation) also had statistically significant and positive Pearson’s correlation coefficients between EVI_all-rice_ and XCH_4_ data at both annual and seasonal scales (R_year_ and R_5–11_), as seen in southern China^36^ (Region 3 in Fig. 4a); however, the area was small due to the limited double-rice croplands^52^. The 0.5 gridcells in regions with double cropping systems of rice and another crop (e.g., rice and winter wheat) had positive R_year_ and R_5–11_ values, but R_5–11_ values were obviously larger than R_year_. These results can be seen in North India and Bangladesh^34,35^ (Region 4 in Fig. 4a, Supplementary Fig. 15), as well as North China^53^ (Region 5 in Fig. 4a).

The relationships between EVI_all-rice_ and XCH_4_ data in regions with heterogeneous annual cropping systems and rotation schedules were more complicated and irregular, and the 0.5° gridcells in these regions had non-significant or negative R_year_ and R_5–11_ values. Such complexities were observed in the Yangtze Plain in southern China and western Thailand, which had a mixture of single- and double-rice cropping systems^36,50–52^, and in Cambodia and southern Vietnam, which had a mixture of single-, double-, and triple-rice cropping systems^50,54^ (Regions 6 and 7 in Fig. 4a; Supplementary Fig. 15). The two measures most likely failed to positively correlate because EVI and XCH_4_ at 0.5° gridcells could be affected by the heterogeneity of cropping systems, crop types, and rice planting schedules, as well as cloud cover. For example, the Mekong Delta of Vietnam has single-, double-, and triple-rice cropping systems that are either rain-fed or irrigated^54^. The EVI and XCH_4_ profiles from this region with mixed cropping systems had irregular seasonal patterns and accordingly weak or negative correlations (Supplementary Fig. 16), which could also be affected by the uncertainty of observed EVI and XCH_4_ due to intensive cloud cover. A similar result was found for the period of 2005–2007, 2007–2009, and 2009–2011 (Supplementary Figs. 17–19). The significant correlations between EVI and XCH_4_ in rice paddy areas with the same or similar cropping systems and planting schedules (Figs. 3, 4; Supplementary Figs. 17–19) demonstrated the role of rice paddies in determining the seasonal dynamics of atmospheric CH_4_ concentration in monsoon Asia.

Our analyses demonstrate that the satellite-observed XCH_4_ is indicative of CH_4_ emissions from rice paddies, and paddy rice cultivation dominates the spatial distribution and seasonal dynamics of atmospheric CH_4_ concentration over those regions with dense rice paddies. This study also shows the importance of annual paddy rice maps for assessing the effects of rice paddies on the spatial pattern and seasonal dynamics of atmospheric CH_4_ concentration by providing more details on the location and area proportion within gridcells. On one hand, these maps could show area fractional information within the 0.5° and 2° gridcells when analyzing spatial relationship between rice paddy area and XCH_4_. On the other hand, they allow us to capture the seasonal dynamics of vegetation indices for individual rice paddies (pixels) when analyzing seasonal relationship between them. The temporal statistics of vegetation indices for all pixels within individual gridcells often differ from those for the pure rice paddy pixels (e.g., Fig. 3j, n). The pure rice paddy pixel-based analyses had clear double peaks (EVI_all-rice_ in Fig. 3n), while only one peak was found for all the pixels (EVI_all-pixels_ in Fig. 3j) due to disturbances from other upland crops or natural vegetation types in the gridcells. Although the 500 m paddy rice maps in this study still had mixed pixel issues, this paddy rice product provided unprecedented details on location and area proportion within the 0.5° or 2° gridcells for the entirety of monsoon Asia, in comparison with previous efforts that used county- or province-level paddy rice data^14,17,55,56^. Therefore, it is necessary to use accurate paddy rice maps at moderate spatial resolution as masks to track seasonal fluctuations of atmospheric CH_4_ concentration.

In addition to the density of rice paddies within gridcells, our results also demonstrate the importance of the information about rice cropping intensity (single or double) and timing of rice crop calendar within gridcells. The seasonal dynamics of XCH_4_, which have one or two peaks in a year, mirror paddy rice growth in the areas dominated by single- and double-rice cropping systems (Fig. 3). Together, the accurate information on the locations of rice paddies allow us to fingerprint the effects of rice paddies on atmospheric CH_4_ concentration, which can reduce influence from other land cover types in the gridcells. Furthermore, the spatial distribution of rice paddies in monsoon Asia has changed substantially since 2000, including a northward shift of rice paddies in China^30^. Therefore, the methane emission simulations should be conducted with annual maps of rice paddies to more accurately estimate the effects of changing paddy rice distribution on methane emissions since 2000.

### Interannual dynamics of rice paddy area and XCH_4_

Recent increases in atmospheric CH_4_ concentration since 2007 are not well understood as evidenced by many hypotheses currently debated^1–11^. Some studies reported that biogenic sources, most notably agriculture, may be the key contributor to renewed growth in atmospheric CH_4_^4,5^. Rice paddy is one of the main agricultural sources of CH_4_ emission. In this study, we also investigated whether interannual variations in rice paddy area contributed to the renewed growth of atmospheric CH_4_ concentration since 2007 at the national and continental scales. We analyzed the interannual variations of XCH_4_ during 2003–2015 in monsoon Asia, especially China and India, the two countries with the largest rice paddy areas.

**Fig. 5:**
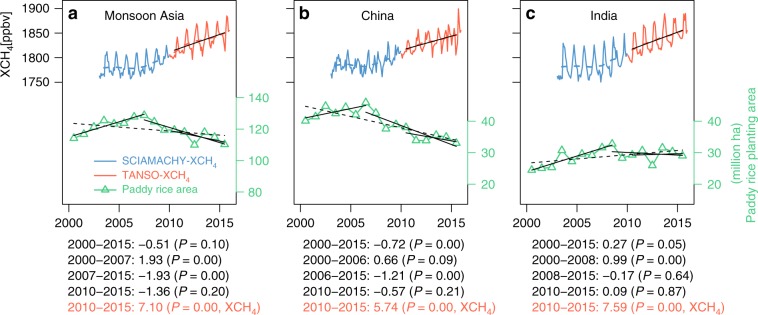
Temporal dynamics of atmospheric methane concentration and rice paddy area. Temporal (seasonal and interannual) dynamics of atmospheric column-averaged methane concentration (XCH_4_) over rice paddy-dominated regions and interannual variations in MODIS-based rice paddy areas during 2000–2015 in monsoon Asia (**a**), China (**b**), and India (**c**). The curves above are time series of monthly SCIAMACHY CH_4_ column volume mixing ratios (VMRs, in parts per billion) during 2003–2009 and monthly TANSO-FTS CH_4_ column VMRs during 2010–2015. The XCH_4_ outliers in winter have been removed. The blue and red dashed lines are average annual values of SCIAMACHY XCH_4_ and TANSO-FTS XCH_4_, respectively. The black lines and black dashed lines below indicate trends of rice paddy area for different periods in monsoon Asia, China, and India. The rice paddy-dominated regions are shown in Supplementary Fig. 35. The linear trends and its significance levels for different periods are shown below the panels; the formulas in black color are for paddy rice planting area, and the formula in red is for XCH_4_. Source data are provided as a Source Data file.

The interannual variations in XCH_4_ in the rice paddy-dominated areas of monsoon Asia, China, and India were relatively stable during 2003–2006, but changed into an increasing trend beginning in 2007 (Fig. 5), which agreed well with the trends of global XCH_4_^1^. The rice paddy area increased during 2003–2006 in China, India, and monsoon Asia, but after 2007 rice paddy area decreased in China and monsoon Asia and remained stable in India (Fig. 5). The total rice paddy area in monsoon Asia has declined since 2007 over the time period of renewed XCH_4_ growth. Similar results were also found for the whole region (rice paddy and non-rice paddy areas) of monsoon Asia, China, and India (Supplementary Fig. 20). In theory, if the interannual trends of rice paddy area and the atmospheric CH_4_ concentration are consistent, we cannot conclude that rice paddy area is the main driver for the regrowth of atmospheric CH_4_ concentration since 2007. If their trends are opposite, we can conclude that the rice paddy area is not the main driver for the regrowth of atmospheric CH_4_ concentration. The decreasing trends of rice paddy area and the increasing trends of atmospheric CH_4_ concentration in both the rice paddy-dominated areas and the whole area since 2007 suggested that the change in rice paddy area was not the main driver for the renewed increase of atmospheric CH_4_ concentration. A study using atmospheric methane observations and an atmospheric transport model reported that the annual methane emission has not significantly changed in India during 2010–2015 and the major CH_4_ sources (ruminants, rice paddies, waste, and fossil fuels) did not much change^57^, which is in line with the stable rice paddy areas in India from this study during the same period (Fig. 5). Another country-scale study showed that rice paddies did not contribute to the increase of atmospheric CH_4_ concentration in China and India during 2010–2015 using the emission estimates from the inverse model and the spatial distribution of different source sectors within the EDGAR emissions inventory^58^, which agrees with our result from 2010–2015 (Fig. 5). Our study at monsoon Asia scale suggests that the renewed growth of atmospheric CH_4_ concentration was unlikely attributed to the dynamics of rice paddy area.

What factors drove the interannual variations of atmospheric CH_4_ concentration since 2007 is still a hotly debated issue^1–11^, as CH_4_ emissions are controlled by multiple sources and sinks. At the rice paddy fields, there are several factors which could affect its methane emission, including paddy rice planting area, cropping system, yield, management (e.g., irrigation, fertilization), and rice varieties^14,59^. For the first time, our study shows the fingerprint of rice paddies in the spatial distribution and seasonal dynamics of atmospheric methane concentration by using unprecedented annual paddy rice maps. We are not in position to do a full-scale (multivariate) attribution analysis on the question why atmospheric XCH_4_ increased again after 2007, thus a thorough explanation of the renewed growth in XCH_4_ since 2007 needs further study.

### Potential of annual paddy rice maps for CH_4_ models

To our knowledge, our annual maps of rice paddies in monsoon Asia during 2000–2015 are the first of its kind and are likely to have significant implications for simulations of biogeochemical models and atmospheric chemistry and transport models. Several empirical and process-based biogeochemical models have been used to predict CH_4_ emissions from rice paddies^13,14,55,56,60^. However, most of the estimates of rice paddy CH_4_ emission from those models were generally driven by rice paddy data from agricultural statistical sources such as province- or county-level administrative units^13,14,59–61^, which led to a large spatial uncertainty in estimates of rice paddy CH_4_ emission. For example, Zhang et al.^60^ estimated CH_4_ emission from rice paddies at the global scale based on inundation areas, statistical data, and additional cropland masks. However, the methane emission estimates in some regions with dense rice paddies were missed due to inaccurate rice paddy distribution information, such as the northwest of Indo-Gangetic Plain in India and Pakistan. The annual maps of rice paddies at moderate spatial resolution in this study offer new opportunities to improve simulations of biogeochemical models for CH_4_ emission from rice paddies.

The improved estimates of CH_4_ emission fluxes with high-accuracy annual paddy rice maps could then be used by atmospheric chemistry and transport models to study the spatial and seasonal consistency between atmospheric CH_4_ concentration and rice paddies, as well as the relative contribution of rice paddies to the interannual dynamics of atmospheric CH_4_ concentration. These new paddy rice maps will likely shed new light on the role of rice paddies in atmospheric CH_4_ concentration and represent a critical step for improving the understanding of long-term dynamics of atmospheric CH_4_ concentration.

Agricultural emissions account for more than half of the total CH_4_ emissions budget in monsoon Asia, of which one-third is estimated to originate from rice paddies^13^. Using annual paddy rice maps at 500-m spatial resolution and spaceborne XCH_4_ data in monsoon Asia, this study clearly characterizes the fingerprints of rice paddies in the spatial distribution and seasonal dynamics of atmospheric CH_4_ concentration over the dense rice paddy regions. It highlights an important impact of rice agriculture on the spatial and seasonal patterns of regional atmospheric CH_4_. The observed changes in the spatial distribution of rice paddies in monsoon Asia during 2000–2015 do warrant urgent and further use of the annual paddy rice maps in biogeochemical model simulations. Considering the fertilization effect of global rising atmospheric CO_2_, climate change, and projected increases in demand for rice in the coming decades, CH_4_ emissions from rice production is expected to rapidly increase^15^, possibly leading to stronger effects on the variability of atmospheric CH_4_ at various spatial and temporal scales. This study also provides new insight to the debates on the driving factors for the renewed growth of atmospheric CH_4_ concentration since 2007 from the perspective of paddy rice planting area; and the results show the inconsistent interannual variations of rice paddy area and XCH_4_. However, considering the complexity in the CH_4_ emission and atmospheric transport, more methane sources and sinks should be considered for a thorough explanation of the renewed growth of atmospheric CH_4_ concentration since 2007.

## Methods

### Atmospheric CH_4_ concentration data

We used the IMAP 7.1 product from SCIAMACHY^62^ and the OCPR7.0 product from TANSO-FTS^63^, which were downloaded from the data portal (http://www.esa-ghg-cci.org/sites/default/files/documents/public/documents/GHG-CCI_DATA.html). The SCIAMACHY XCH_4_ data during 2003–2011 and the TANSO-FTS XCH_4_ data during 2010–2015 were arranged into 0.5° × 0.5° and 2° × 2° grids in latitude and longitude, respectively, and then composited it into monthly average XCH_4_.

### MODIS data

We used MODIS Collection-6 surface reflectance, land surface temperature (LST), and land cover type to extract rice paddies in monsoon Asia. The 8-day composite surface reflectance products from EOS-Terra-MODIS (MOD09A1) at 500 m spatial resolution during 2000–2015 were used to calculate three spectral indices: EVI^32^, Land Surface Water Index (LSWI)^64^, and Normalized Difference Snow Index (NDSI)^65^. The bad observations containing clouds or snow were excluded before mapping rice paddies. We used the 8-day composite LST products from EOS-Aqua-MODIS (MYD11A2) at 1 km spatial resolution during 2003–2015 to determine the start and end of thermal growing season (Supplementary Fig. 21) and the time window of rice transplanting. The EOS-Terra-MODIS (MOD12Q1) provided wetland maps as a non-crop mask based on the land cover scheme of International Geosphere-Biosphere Programme (IGBP) (Supplementary Fig. 22). More detailed MODIS data processing is stated in Supplementary Note 2.

### Paddy rice mapping

The annual maps of paddy rice in monsoon Asia at 500 m resolution from 2000–2015 were developed using time series MODIS data and a well-documented phenology-based algorithm^28–31^. The flooding signal during the rice transplanting phase is a critical feature used to identify rice paddies, as paddy rice is the sole crop type to be transplanted and grown in water–soil mixture fields. Through temporal profile analysis of remote sensing data, the mixture of rice plants and water (open canopy) during the transplanting phase can be tracked by the relationship between the vegetation greenness index (i.e., EVI) and the water index (i.e., LSWI) simply using the equation: LSWI + 0.05 ≥ EVI^28,29^. We recently improved the algorithm by incorporating the temperature-based time window for rice transplanting, which was derived from MODIS LST data^31^. We applied this method successfully in China and India in our previous study^30^. Here, the algorithm was used for monsoon Asia over the period of 2000–2015 (Supplementary Note 3).

In order to improve the accuracy of the MODIS-based paddy rice mapping, several non-cropland masks were generated^28–31^, including evergreen vegetation, forest, sparse vegetation, natural wetlands, topographic masks, and a temperate-based mask (Supplementary Note 3, Supplementary Fig. 22). After excluding areas using these masks, annual paddy rice maps were generated.

The resultant maps were then validated through comparing with existing products in different countries (Supplementary Note 4 and Supplementary Figs. 23–26), as well as the statistical data of FAO Statistical Databases (FAOSTAT) at national scale during 2000–2015 (Supplementary Note 4 and Supplementary Fig. 27). We also validated the maps based on pixel-level comparison using higher resolution Landsat-based paddy rice maps in sample regions (Supplementary Note 4, Supplementary Fig. 28, and Supplementary Table 3). The validation and comparison results showed the continental-scale paddy rice maps were reliable. To analyze the spatial and seasonal relationships between rice paddies and satellite-observed XCH_4_, the paddy rice maps with 500 m resolution (binary map with 0 or 1) were aggregated to 0.5° and 2° gridcells with rice paddy area proportions to match the resolutions of SCIAMACHY and TANSO-FTS based XCH_4_, respectively (Supplementary Fig. 29).

### Trend analysis of rice paddy area

We investigated the spatial pattern of changes in rice paddy area in monsoon Asia using the least square method. We aggregated binary paddy rice maps into fractional rice maps within 10 × 10 pixel gridcells (~5 km spatial resolution) and calculated a spatially explicit map of the linear trend of rice paddy area changes from 2000–2015 within the gridcells (Fig. 1b).

### Analysis of spatial consistency between rice paddy and XCH_4_

We investigated the spatial correlation between XCH_4_ from SCIAMACHY/TANSO-FTS and MODIS-based rice paddy area percentage over six 3-year periods (2003–2005, 2005–2007, 2007–2009, 2009–2011, 2011–2013, and 2013–2015), forming a complete coverage in all the 3-year moving windows from 2003 to 2015 (Supplementary Fig. 30). The composites of six periods of observations were set considering the available years of the observed atmospheric CH_4_ concentration data and the effects of potential abnormal observations in some grids and certain year. First, we analyzed variations in 3-year average yearly and seasonal XCH_4_ within different rice paddy density gradients to see whether the variations of XCH_4_ and rice paddy area were consistent. Then, the spatial error model (SEM)^66^ was used to evaluate the correlation between XCH_4_ and rice paddy area percentage in monsoon Asia, as these two variables spatially auto-correlate over geographical distribution, which would affect correlation analyses between them. Taking the data during 2003–2005 for example, we tested the spatial autocorrelation of our dependent variable (XCH_4_) and found a significant autocorrelation, with Moran’s I equal to 0.83 (*P* < 0.001) (Supplementary Table 1). When we used the ordinary least-squares model (OLS) to analyze the spatial relationship between XCH_4_ and paddy rice croplands, the Moran’s I of model residual was 0.80 with *P* < 0.001 (Supplementary Table 4), showing the relationship did not satisfy the hypothesis of independent identical distribution and OLS was inapplicable. We then used the SEM to analyze the spatial relationship between them, and Moran’s I of model residual was −0.15 with *P* equal to 1 (Supplementary Table 1), indicating that the SEM-based result was reliable. The SEM coefficients showed that there was a significant positive correlation (all of *P*-value < 0.001) between spatial distributions of XCH_4_ and rice paddy area percentage during the six 3-year periods (Supplementary Table 1).

### Analysis of seasonal dynamics of paddy rice growth and XCH_4_

The effects of paddy rice growth on the seasonal variations of XCH_4_ were examined by time series analysis of XCH_4_ and MODIS-based EVI (as a proxy of rice plant growth), over four typical regions of interest (ROIs) with dense rice paddy areas and different rice cropping systems, and the rice paddy-distributed region in monsoon Asia. We also compared landscape scale EVI data and CH_4_ emissions from eight existing rice paddy eddy flux sites to verify the seasonal consistency between observed CH_4_ emission and rice plant growth^25–27,41,42,45–47^ (Supplementary Fig. 14). Given the small size of the study plots at these flux sites, the EVI with 250 -m resolution derived from MOD13Q1 during the corresponding period was used to characterize the paddy rice growth. At the regional scale, four ROIs (Fig. 3a–d) with different rice phenology and cropping systems were extracted from dense rice paddy gridcells with rice paddy proportions over 20% (Supplementary Fig. 31) to analyze the seasonal relationships between the growth of paddy rice and XCH_4_. We calculated mean EVI values from different statistical approaches: all pixels within 0.5° gridcells (EVI_all-pixels_), all rice paddy pixels within 0.5° gridcells (EVI_all-rice_), and single rice paddy pixel with 500 m resolution (EVI_rice_). Then we analyzed the seasonal dynamics of XCH_4_ and three EVI proxies in these four ROIs (Fig. 3), by using correlation analysis for the entire year (R_year_) and summer-fall season (R_5–11_, rice growing season in the wheat–rice or other cropping system). Monsoon Asia is dominated by single- and double-cropping system^52,54,67,68^, and the triple-cropping is very limited in some regions like Mekong Delta^54,67^ (Supplementary Fig. 32). The main growing season of rice plants in monsoon Asia are mostly during summer-fall season from May to November (Supplementary Figs. 33, 34) based on statistics on the monthly distribution of rice paddy area for Asia^69^. In order to reflect the seasonal controls of paddy rice growth on atmospheric CH_4_ concentration, the period of the major rice cycle (from May to November) is used to analyze the seasonal relationships between them. At the continental scale, a wall-to-wall correlation analysis was used to quantify the relationship between the seasonal variations of XCH_4_ and EVI at 0.5° gridcells for the entire year (R_year_) and summer-fall season (R_5–11_) during 2003–2005 (Fig. 4), 2005–2007, 2007–2009, and 2009–2011 (Supplementary Figs. 17–19). The periods after 2011 were not considered due to the coarse resolution of the TANSO-FTS XCH_4_ data. The region with a 3-year averaged rice paddy gridcells with area proportion larger than 10% during the corresponding period was considered here.

### Analysis of interannual variations of rice paddy and XCH_4_

The interannual variations and trends in MODIS-based rice paddy areas and XCH_4_ were analyzed to examine the potential contribution of rice paddy area dynamics to atmospheric CH_4_ concentration in monsoon Asia, China, and India. The analyses were conducted from two levels: the rice paddy-dominated area (Fig. 5) and the whole area of monsoon Asia, China, and India (Supplementary Fig. 20). The rice paddy-dominated area here meant those gridcells (0.5° × 0.5°) with maximum rice paddy area proportion larger than 10% during 2000–2015 (Supplementary Fig. 35).

### Rice paddy CH_4_ emission reflected by estimates of EDGAR

We used the independent bottom-up estimates of Emissions Database for Global Atmospheric Research (EDGAR) data sets v4.3.2^70^ to analyze the relative contribution of rice agriculture to total CH_4_ emission. The result showed that the paddy rice cultivation is the dominated sector for the CH_4_ emission in the paddy rice planting area of monsoon Asia (Supplementary Fig. 36); specifically, the relative contribution of paddy rice cultivation CH_4_ emission is >30% in the gridcells where the rice paddy area proportion is >5%. This added analysis can help us to understand the fingerprint of rice paddies in the spatial distribution and seasonal dynamics of atmospheric methane concentration in monsoon Asia.

### Reporting summary

Further information on research design is available in the Nature Research Reporting Summary linked to this article.

## Supplementary information

Supplementary Information

Reporting Summary

## Data Availability

The atmospheric CH_4_ data were derived from the SCIAMACHY and TANSO-FTS data sets, available from the data portal (http://www.esa-ghg-cci.org/sites/default/files/documents/public/documents/GHG-CCI_DATA.html). The EDGAR CH_4_ emissions inventory data are downloaded from http://edgar.jrc.ec.europa.eu/. The paddy rice maps can be accessed by contacting Geli Zhang, Xiangming Xiao or Jinwei Dong. All the relevant data from this study are also available from the corresponding authors upon request.

## Code availability

Codes were written in R and will be made available upon request by contacting the corresponding authors.

## References

[1] Nisbet EG, Dlugokencky EJ, Bousquet P (2014). Methane on the rise-again. Science.

[2] Kai FM, Tyler SC, Randerson JT, Blake DR (2011). Reduced methane growth rate explained by decreased Northern Hemisphere microbial sources. Nature.

[3] Kirschke S (2013). Three decades of global methane sources and sinks. Nat. Geosci..

[4] Schaefer H (2016). A 21st-century shift from fossil-fuel to biogenic methane emissions indicated by (CH4)-C-13. Science.

[5] Nisbet EG (2016). Rising atmospheric methane: 2007–2014 growth and isotopic shift. Glob. Biogeochem. Cy.

[6] Schwietzke S (2016). Upward revision of global fossil fuel methane emissions based on isotope database. Nature.

[7] Turner AJ, Frankenberg C, Wennberg PO, Jacob DJ (2017). Ambiguity in the causes for decadal trends in atmospheric methane and hydroxyl. Proc. Natl Acad. Sci. USA.

[8] Rigby M (2017). Role of atmospheric oxidation in recent methane growth. Proc. Natl Acad. Sci. USA.

[9] Prather MJ, Holmes CD (2017). Overexplaining or underexplaining methane’s role in climate change. Proc. Natl Acad. Sci. USA.

[10] Allen G (2016). Biogeochemistry: Rebalancing the global methane budget. Nature.

[11] Turner AJ, Frankenberg C, Kort EA (2019). Interpreting contemporary trends in atmospheric methane. Proc. Natl Acad. Sci. USA.

[12] Newton A (2016). Atmospheric methane: shifting sources. Nat. Geosci..

[13] Saunois M (2016). The global methane budget: 2000-2012. Earth Syst. Sci. Data.

[14] Yan X, Akiyama H, Yagi K, Akimoto H (2009). Global estimations of the inventory and mitigation potential of methane emissions from rice cultivation conducted using the 2006 Intergovernmental Panel on Climate Change Guidelines. Glob. Biogeochem. Cy.

[15] van Groenigen KJ, van Kessel C, Hungate BA (2013). Increased greenhouse-gas intensity of rice production under future atmospheric conditions. Nat. Clim. Change.

[16] Elert E (2014). Rice by the numbers: a good grain. Nature.

[17] Hayashida S (2013). Methane concentrations over Monsoon Asia as observed by SCIAMACHY: signals of methane emission from rice cultivation. Remote Sens Environ..

[18] Bloom AA (2010). Large-scale controls of methanogenesis inferred from methane and gravity spaceborne data. Science.

[19] Frankenberg C (2005). Assessing methane emissions from global space-borne observations. Science.

[20] Kort EA (2014). Four corners: the largest US methane anomaly viewed from space. Geophys Res. Lett..

[21] Buchwitz M (2017). Satellite-derived methane hotspot emission estimates using a fast data-driven method. Atmos. Chem. Phys..

[22] Jacob DJ (2016). Satellite observations of atmospheric methane and their value for quantifying methane emissions. Atmos. Chem. Phys..

[23] Matthews E (1983). Global vegetation and land use: new high-resolution data bases for climate studies. J. Clim. Appl Meteorol..

[24] Hatala JA (2012). Greenhouse gas (CO_2_, CH_4_, H_2_O) fluxes from drained and flooded agricultural peatlands in the Sacramento-San Joaquin Delta. Agriculture, Ecosyst. Environ..

[25] McMillan AMS, Goulden ML, Tyler SC (2007). Stoichiometry of CH_4_ and CO_2_ flux in a California rice paddy. J. Geophys Res-Biogeo.

[26] Meijide A (2011). Seasonal trends and environmental controls of methane emissions in a rice paddy field in Northern Italy. Biogeosciences.

[27] Bhattacharyya P (2014). Tropical low land rice ecosystem is a net carbon sink. Agr. Ecosyst. Environ..

[28] Xiao X (2006). Mapping paddy rice agriculture in South and Southeast Asia using multi-temporal MODIS images. Remote Sens Environ..

[29] Xiao X (2005). Mapping paddy rice agriculture in southern China using multi-temporal MODIS images. Remote Sens Environ..

[30] Zhang G (2017). Spatiotemporal patterns of paddy rice croplands in China and India from 2000 to 2015. Sci. Total Environ..

[31] Zhang G (2015). Mapping paddy rice planting areas through time series analysis of MODIS land surface temperature and vegetation index data. ISPRS J. Photogramm. Remote Sens..

[32] Huete A (2002). Overview of the radiometric and biophysical performance of the MODIS vegetation indices. Remote Sens Environ..

[33] Xin FF (2017). Modeling gross primary production of paddy rice cropland through analyses of data from CO2 eddy flux tower sites and MODIS images. Remote Sens Environ..

[34] Lobell DB, Sibley A, Ortiz-Monasterio JI (2012). Extreme heat effects on wheat senescence in India. Nat. Clim. Change.

[35] Shelley, I. et al. Rice cultivation in Bangladesh: present scenario, problems, and prospects. *J. Int. Coop. Agric. Develop.* **14**, 20–29 (2016).

[36] Li P (2012). Changes in rice cropping systems in the Poyang Lake Region, China during 2004-2010. J. Geogr. Sci..

[37] Huang Y, Sass RL, Fisher FM (1997). Methane emission from Texas rice paddy soils. 2. Seasonal contribution of rice biomass production to CH_4_ emission. Glob. Change Biol..

[38] Hayashi K (2015). Cropland soil-plant systems control production and consumption of methane and nitrous oxide and their emissions to the atmosphere. Soil Sci. Plant Nutr..

[39] Sass RL, Fisher FM, Harcombe PA, Turner FT (1990). Methane production and emission in a Texas rice field. Glob. Biogeochem. Cy.

[40] Huang Y (2001). Comparison of field measurements of CH_4_ emission from rice cultivation in Nanjing, China and in Texas, USA. Adv. Atmos. Sci..

[41] Knox SH (2016). Biophysical controls on interannual variability in ecosystem-scale CO_2_ and CH_4_ exchange in a California rice paddy. J. Geophys. Res.-Biogeosciences.

[42] Alberto MCR (2014). Measuring methane flux from irrigated rice fields by eddy covariance method using open-path gas analyzer. Field Crop Res.

[43] Gogoi N, Baruah K, Gogoi B, Gupta PK (2008). Methane emission from two different rice ecosystems (Ahu and Sali) at lower Brahmaputra Valley Zone of North East India. Appl Ecol. Env Res..

[44] Neue HU (1997). Factors and processes controlling methane emissions from rice fields. Nutrient Cycl. Agroecosystems.

[45] Ge H-X (2018). The characteristics of methane flux from an irrigated rice farm in East China measured using the eddy covariance method. Agric. For. Meteorol..

[46] Zhang, Y. et al. Simulation and estimation of methane emissions from rice paddies in Sanjiang Plain of the Northeast China. *Trans. Chin. Soc. Agric. Eng.* **27**, 293–298 (2011).

[47] Zhao, M. et al. Simulation of greenhouse gas effluxes in rice fields based on DNDC model. *Chin. J. Ecol.* **38**, 1057–1066 (2019).

[48] Watanabe A, Takeda T, Kimura M (1999). Evaluation of origins of CH_4_ carbon emitted from rice paddies. J. Geophys Res-Atmos..

[49] Tokida T (2011). Methane and soil CO_2_ production from current-season photosynthates in a rice paddy exposed to elevated CO_2_ concentration and soil temperature. Glob. Change Biol..

[50] Chen CF, Chen CR, Son NT, Chang LY (2012). Delineating rice cropping activities from MODIS data using wavelet transform and artificial neural networks in the Lower Mekong countries. Agr. Ecosyst. Environ..

[51] Lv TT, Liu C (2010). Extraction of information of cultivated land using time-series MODIS data in Thailand. Trans. Chin. Soc. Agric Eng..

[52] Yan H (2019). Tracking the spatio-temporal change of cropping intensity in China during 2000–2015. Environ. Res. Lett..

[53] Wang J (2015). Mapping paddy rice planting area in wheat-rice double-cropped areas through integration of Landsat-8 OLI, MODIS, and PALSAR images. Sci. Rep..

[54] Son N-T (2013). A phenology-based classification of time-series MODIS data for rice crop monitoring in Mekong Delta, Vietnam. Remote Sens..

[55] Fung I (1991). Three-dimensional model synthesis of the global methane cycle. J. Geophys. Res.: Atmospheres.

[56] Li C (2002). Reduced methane emissions from large-scale changes in water management of China’s rice paddies during 1980-2000. Geophys Res. Lett..

[57] Ganesan AL (2017). Atmospheric observations show accurate reporting and little growth in India’s methane emissions. Nat. Commun..

[58] Miller SM (2019). China’s coal mine methane regulations have not curbed growing emissions. Nat. Commun..

[59] Tian H (2016). The terrestrial biosphere as a net source of greenhouse gases to the atmosphere. Nature.

[60] Zhang B (2016). Methane emissions from global rice fields: magnitude, spatiotemporal patterns, and environmental controls. Glob. Biogeochem. Cy.

[61] Matthews E, Fung I, Lerner J (1991). Methane emission from rice cultivation: geographic and seasonal distribution of cultivated areas and emissions. Glob. Biogeochem. Cy.

[62] Frankenberg C (2011). Global column-averaged methane mixing ratios from 2003 to 2009 as derived from SCIAMACHY: trends and variability. J. Geophys Res..

[63] Parker R (2011). Methane observations from the Greenhouse Gases Observing SATellite: comparison to ground-based TCCON data and model calculations. Geophys Res. Lett..

[64] Xiao X (2002). Observation of flooding and rice transplanting of paddy rice fields at the site to landscape scales in China using VEGETATION sensor data. Int. J. Remote Sens.

[65] Hall DK (2002). MODIS snow-cover products. Remote Sens Environ..

[66] Baltagi BH, Song SH, Koh W (2003). Testing panel data regression models with spatial error correlation. J. Econ..

[67] Xiao XM (2007). Remote sensing, ecological variables, and wild bird migration related to outbreaks of highly pathogenic H5N1 avian influenza. J. Wildl. Dis..

[68] Li P (2016). Mapping rice cropping systems using Landsat-derived Renormalized Index of Normalized Difference Vegetation Index (RNDVI) in the Poyang Lake Region, China. Front. Earth Sci..

[69] Laborte AG (2017). RiceAtlas, a spatial database of global rice calendars and production. Sci. Data.

[70] Janssens-Maenhout G (2017). EDGAR v4.3.2 global atlas of the three major greenhouse gas emissions for the period 1970-2012. Earth Syst. Sci. Data Discuss.

