# Supplementary material for: Fingerprint of rice paddies in spatial–temporal dynamics of atmospheric methane concentration in monsoon Asia
Source: Nat Commun. 2020 Jan 28;11:554. doi: 10.1038/s41467-019-14155-5 (PMC6987195; doi:10.1038/s41467-019-14155-5)
Supplement: Supplementary file 1 — Supplementary Information [file 41467_2019_14155_MOESM1_ESM.pdf]

Supporting Information

Fingerprint of rice paddies in spatial-temporal dynamics of atmospheric methane concentration in monsoon Asia

Zhang, et al.

Includes:

Supplementary Figures 1-36

Supplementary Tables 1-4

Supplementary Notes 1-4

Supplementary References

Supplementary Figure 1

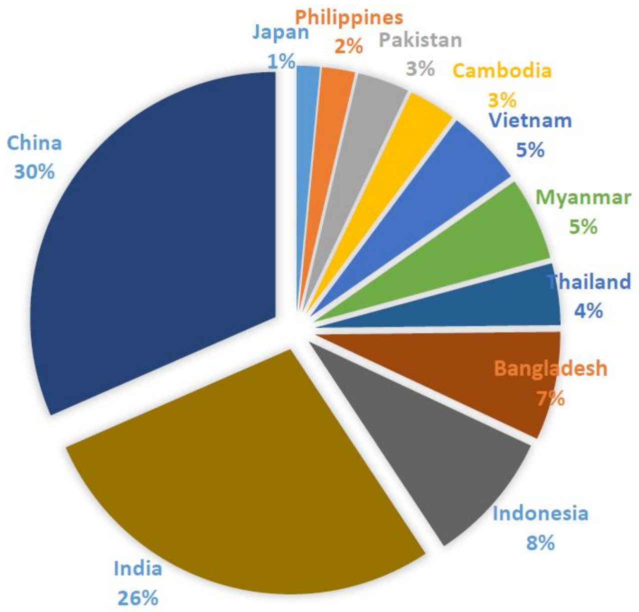

Supplementary Figure 1. Area proportions of MODIS-based paddy rice croplands of the major rice-planting countries in monsoon Asia in 2015.

Supplementary Figure 2

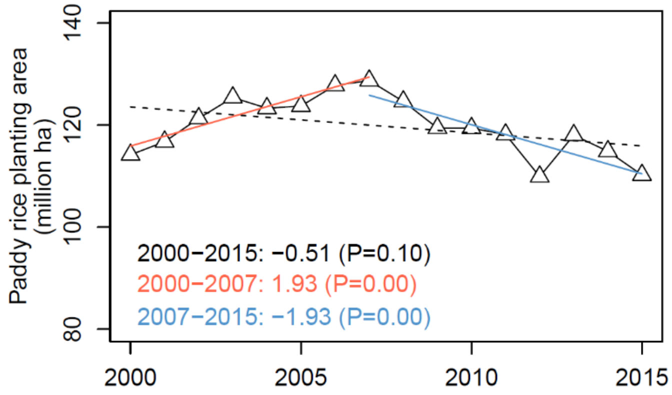

Supplementary Figure 2. Interannual variation of MODIS-based paddy rice croplands during 2000–2015 in monsoon Asia. The black dashed line, red line, and blue line indicate trends of rice paddy area in monsoon Asia for the period of 2000–2015, 2000–2007, and 2007–2015, respectively. The linear trend and its significance level, P, for the three periods are shown in the figure.

Supplementary Figure 3

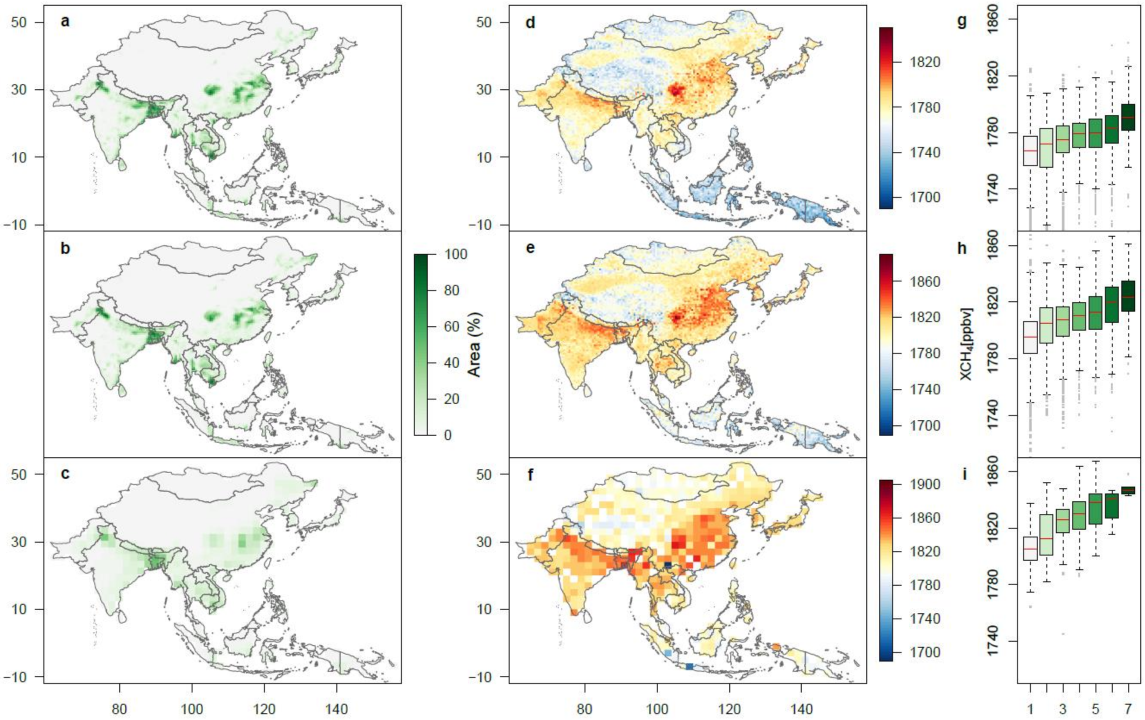

Supplementary Figure 3. Consistency of spatial distributions between paddy rice croplands and atmospheric methane concentration. **a-c** Spatial distributions of multi-year averaged area proportions of MODIS-based paddy rice croplands for 2005–2007 with 0.5° gridcells (**a**), 2009–2011 with 0.5°gridcells (**b**), and 2013–2015 with 2.0°gridcells (**c**). **d-f** Spatial distributions of multi-year averaged annual column-averaged concentration of CH_4 (XCH_4) from SCHAMACHY for 2005–2007 with 0.5° resolution (**d**), 2009–2011 with 0.5° resolution (**e**), and from TANSO-FTS for 2013–2015 with 2.0° resolution (**f**). **g-i** Multi-year averaged annual XCH_4 for different rice paddy area proportions for 2005–2007 (**g**), 2009–2011 (**h**), and 2013–2015 (**i**). The x-axis values in figures **g-i** represent levels of rice paddy area proportions in monsoon Asia, and 1–7 correspond to <0.5%, 0.5~1%, 1~5%, 5~10%, 10~20%, 20~40%, and >40% rice paddy area proportions, respectively.

Supplementary Figure 4

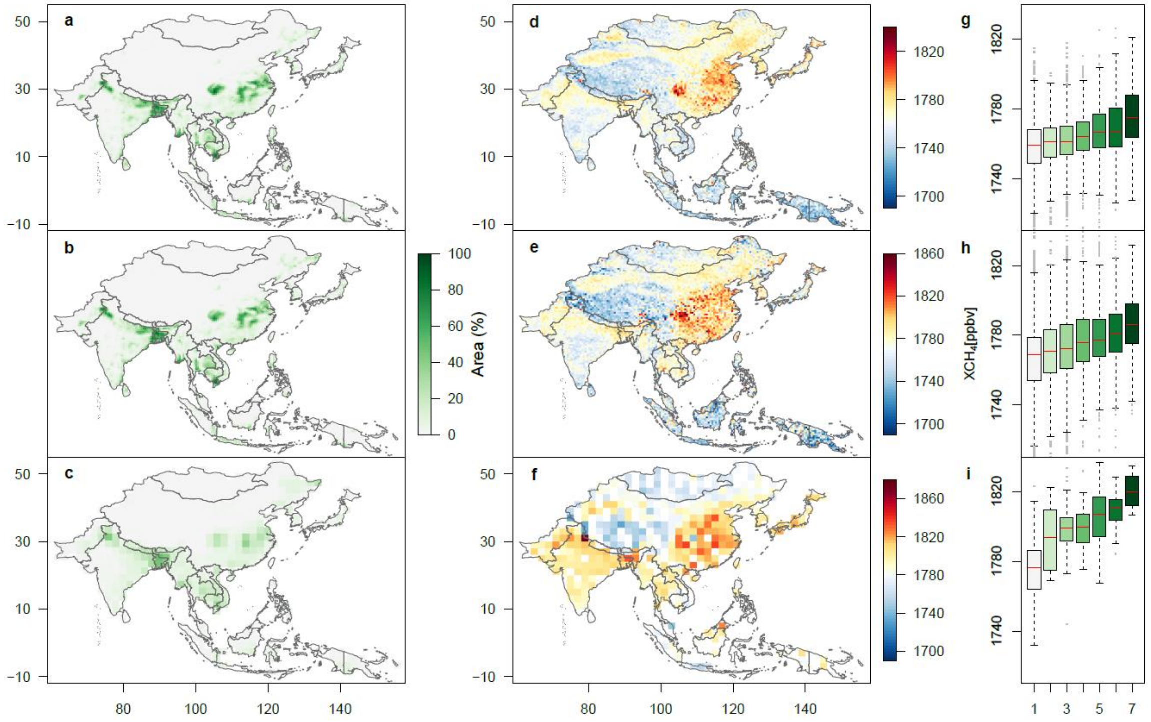

Supplementary Figure 4. Consistency of spatial distributions between rice paddies and atmospheric methane concentration in monsoon Asia. The three periods (2003-2005, 2007-2009, and 2011-2013) were selected to illustrate the spatial relationships between them for spring. **a-c** Spatial distributions of multi-year averaged area proportions of MODIS-based rice paddies for 2003–2005 with 0.5° (latitude/longitude) gridcells (**a**), 2007–2009 with 0.5°gridcells (**b**), and 2011–2013 with 2.0°gridcells (**c**). **d-f** Spatial distributions of multi-year averaged spring column-averaged concentration of CH_4 (XCH_4) from SCHAMACHY for 2003–2005 with 0.5° resolution (**d**), 2007–2009 with 0.5° resolution (**e**), and from TANSO-FTS for 2011–2013 with 2.0° resolution (**f**). **g-i** Multi-year averaged spring XCH_4 for different rice paddy area proportions for 2003–2005 (**g**), 2007–2009 (**h**), and 2011–2013 (**i**). The x-axis values in figure **g-i** represent levels of rice paddy area proportions in monsoon Asia, and 1–7 correspond to <0.5%,

0.5~1%, 1~5%, 5~10%, 10~20%, 20~40%, and >40% rice paddy area proportions, respectively.

Supplementary Figure 5

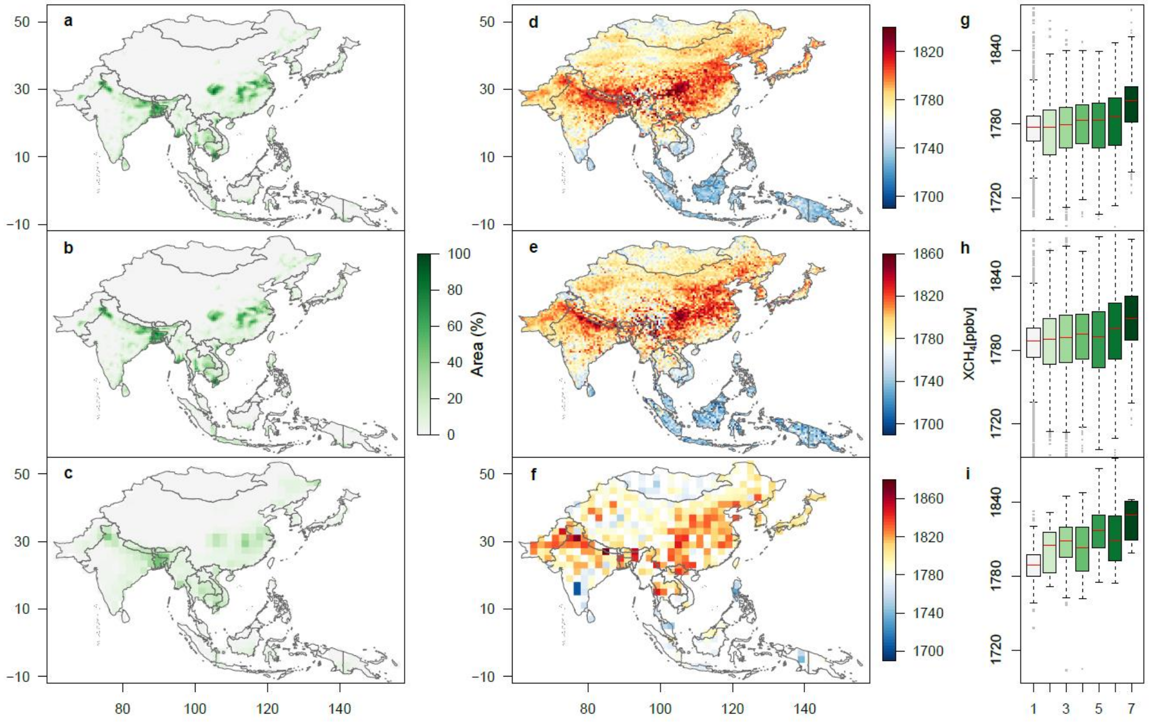

Supplementary Figure 5. Consistency of spatial distributions between rice paddies and atmospheric methane concentration in monsoon Asia. The three periods (2003-2005, 2007-2009, and 2011-2013) were selected to illustrate the spatial relationships between them for summer. **a-c** Spatial distributions of multi-year averaged area proportions of MODIS-based rice paddies for 2003–2005 with 0.5°gridcells (**a**), 2007–2009 with 0.5°gridcells (**b**), and 2011–2013 with 2.0°gridcells (**c**). **d-f** Spatial distributions of multi-year averaged summer column-averaged concentration of CH_4 (XCH_4) from SCHAMACHY for 2003–2005 with 0.5° resolution (**d**), 2007–2009 with 0.5° resolution (**e**), and from TANSO-FTS for 2011–2013 with 2.0° resolution (**f**). **g-i** Multi-year averaged summer XCH_4 for different rice paddy area proportions for 2003–2005 (**g**), 2007–2009 (**h**), and 2011–2013 (**i**). The x-axis values in figure **g-i** represent levels of rice

paddy area proportions in monsoon Asia, and 1–7 correspond to <0.5%, 0.5~1%, 1~5%, 5~10%, 10~20%, 20~40%, and >40% rice paddy area proportions, respectively.

Supplementary Figure 6

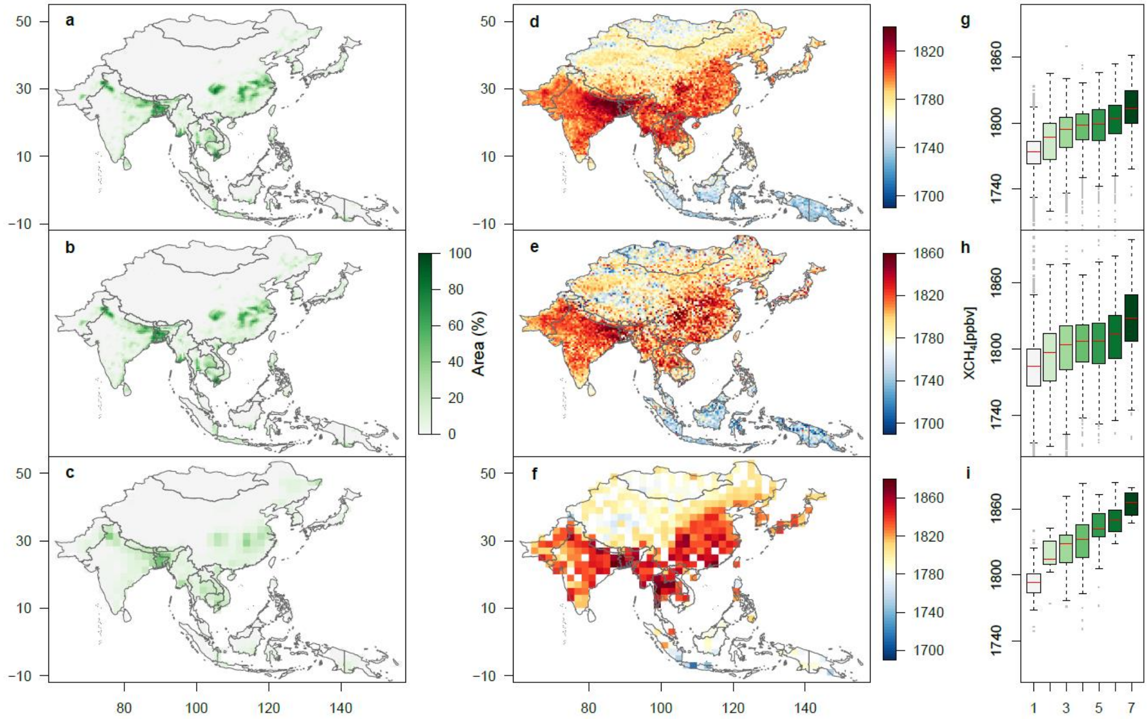

Supplementary Figure 6. Consistency of spatial distributions between rice paddies and atmospheric methane concentration in monsoon Asia. The three periods (2003-2005, 2007-2009, and 2011-2013) were selected to illustrate the spatial relationships between them for autumn. **a-c** Spatial distributions of multi-year averaged area proportions of MODIS-based rice paddies for 2003–2005 with 0.5°gridcells (**a**), 2007–2009 with 0.5°gridcells (**b**), and 2011–2013 with 2.0°gridcells (**c**). **d-f** Spatial distributions of multi-year averaged autumn column-averaged concentration of CH_4 (XCH_4) from SCHAMACHY for 2003–2005 with 0.5° resolution (**d**), 2007–2009 with 0.5° resolution (**e**), and from TANSO-FTS for 2011–2013 with 2.0° resolution (**f**). **g-i** Multi-year averaged autumn XCH_4 for different rice paddy area proportions for 2003–2005 (**g**), 2007–2009 (**h**), and 2011–2013 (**i**). The x-axis values in figure **g-i** represent levels of rice

paddy area proportions in monsoon Asia, and 1–7 correspond to <0.5%, 0.5~1%, 1~5%, 5~10%, 10~20%, 20~40%, and >40% rice paddy area proportions, respectively.

Supplementary Figure 7

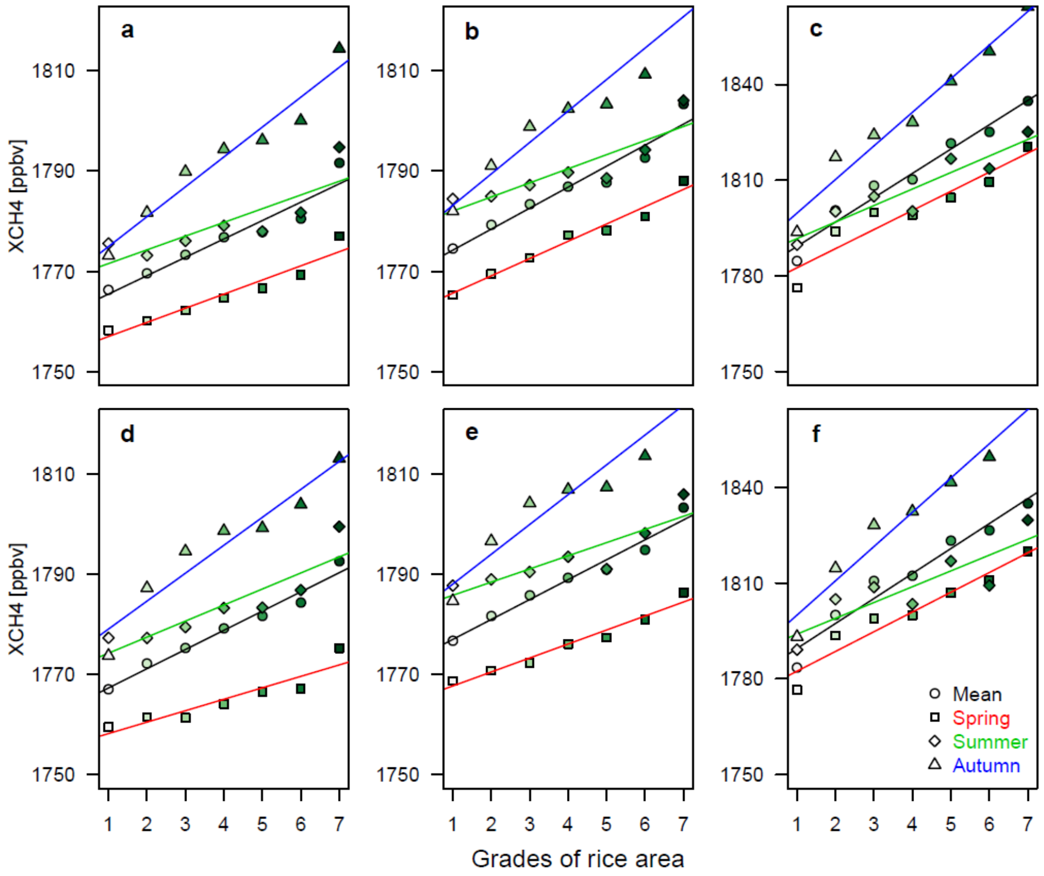

Supplementary Figure 7. Changes in mean (**a-c**) and median (**d-f**) of yearly and seasonal atmospheric column-averaged methane concentration (XCH_4) for different rice paddy area proportions for 2003–2005 (**a, d**), 2007–2009 (**b, e**), and 2011–2013 (**c, f**). These three periods mentioned above were selected to illustrate the changes in XCH_4 for different seasons. The trend lines with different colors mean the column-averaged concentration of CH_4 (XCH_4) during different periods. The x-coordinate values represent levels of rice paddy area proportions in monsoon Asia. 1–7 correspond to <0.5%, 0.5~1%, 1~5%, 5~10%, 10~20%, 20~40%, and >40% rice paddy area proportions, respectively.

Supplementary Figure 8

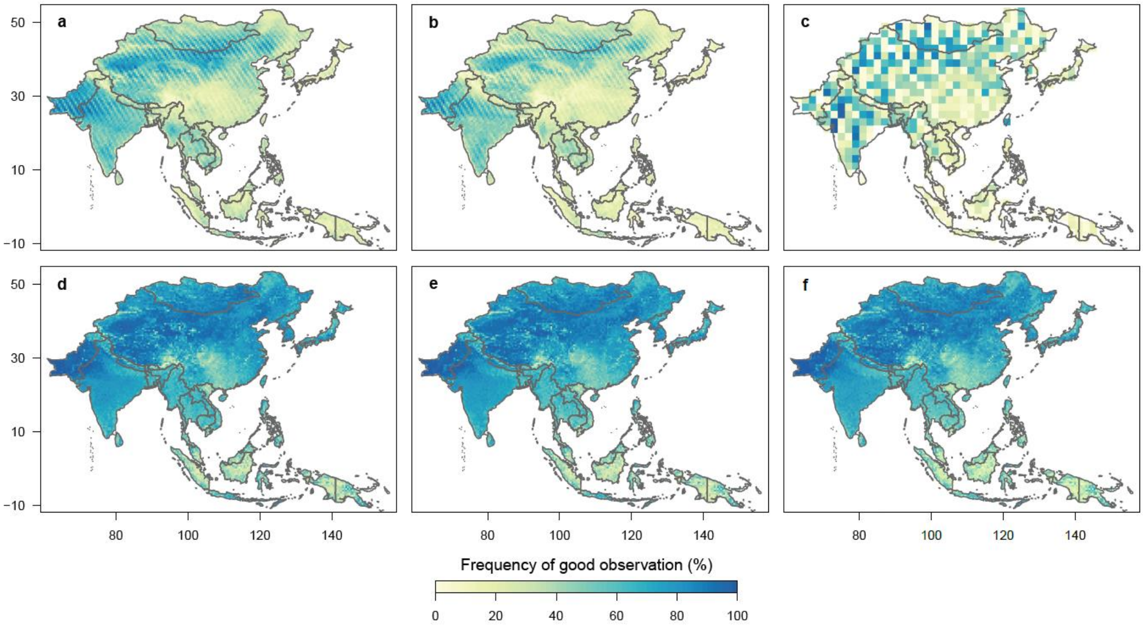

Supplementary Figure 8. Good observations of satellite-based atmospheric methane concentration data (XCH_4) and MODIS data. The three periods (2003-2005, 2007-2009, and 2011-2013) were selected to show the spatial patterns of good observations of XCH_4 and MODIS data. **a-c** are from SCIAMACHY XCH_4 data during 2003-2005 and 2005-2007, and TANSO-FTS XCH_4 data during 2011-2013, respectively. **d-f** are from EOS-Terra-MODIS (MOD09A1) during 2003-2005, 2005-2007, and 2011-2013, respectively.

Supplementary Figure 9

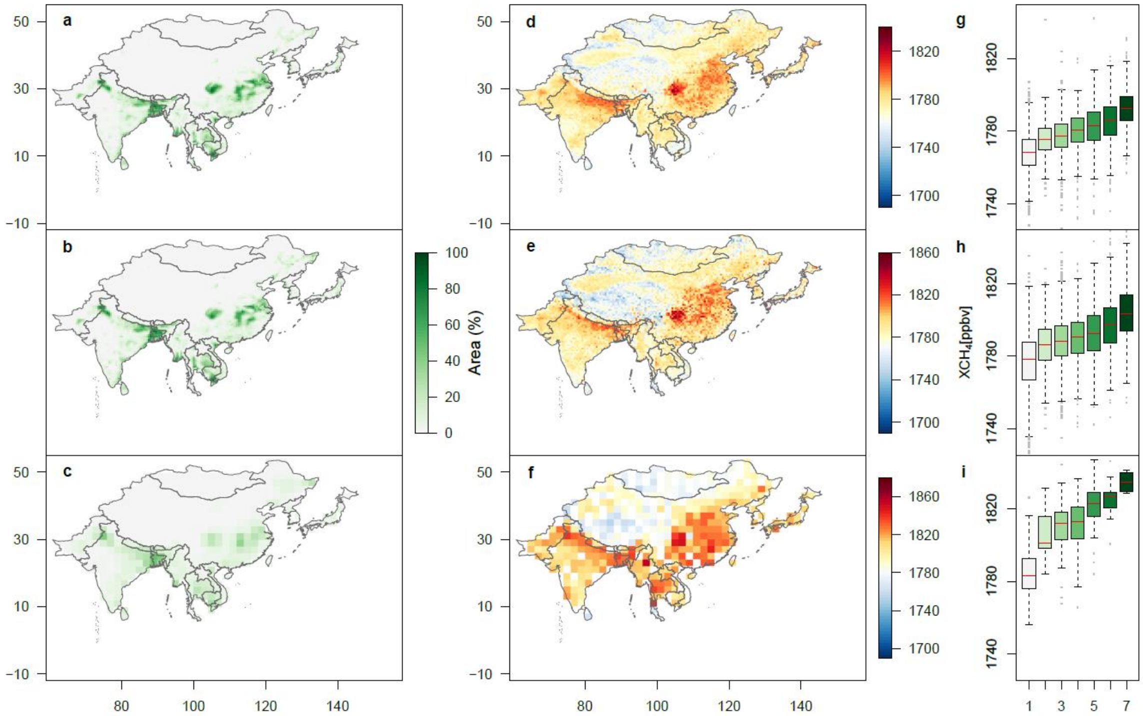

Supplementary Figure 9. Consistency of spatial distributions between paddy rice croplands and atmospheric methane concentration but for Indonesia and Malaysia. The three periods (2003-2005, 2007-2009, and 2011-2013) were selected to illustrate the spatial relationships between them. **a-c** Spatial distributions of multi-year averaged area proportions of MODIS-based paddy rice croplands for 2003–2005 with 0.5° gridcells (**a**), 2007–2009 with 0.5°gridcells (**b**), and 2011–2013 with 2.0°gridcells (**c**). **d-f** Spatial distributions of multi-year averaged annual column-averaged concentration of CH_4 (XCH_4) from SCHAMACHY for 2003–2005 with 0.5° resolution (**d**), 2007–2009 with 0.5° resolution (**e**), and from TANSO-FTS for 2011–2013 with 2.0° resolution (**f**). **g-i** Multi-year averaged annual XCH_4 for different rice paddy area proportions for 2003–2005 (**g**), 2007–2009 (**h**), and 2011–2013 (**i**). The x-axis values in figures **g-i** represent levels of rice paddy area proportions in monsoon Asia, and 1–7 correspond to <0.5%,

0.5~1%, 1~5%, 5~10%, 10~20%, 20~40%, and >40% rice paddy area proportions, respectively. Source data are provided as a Source Data file.

Supplementary Figure 10

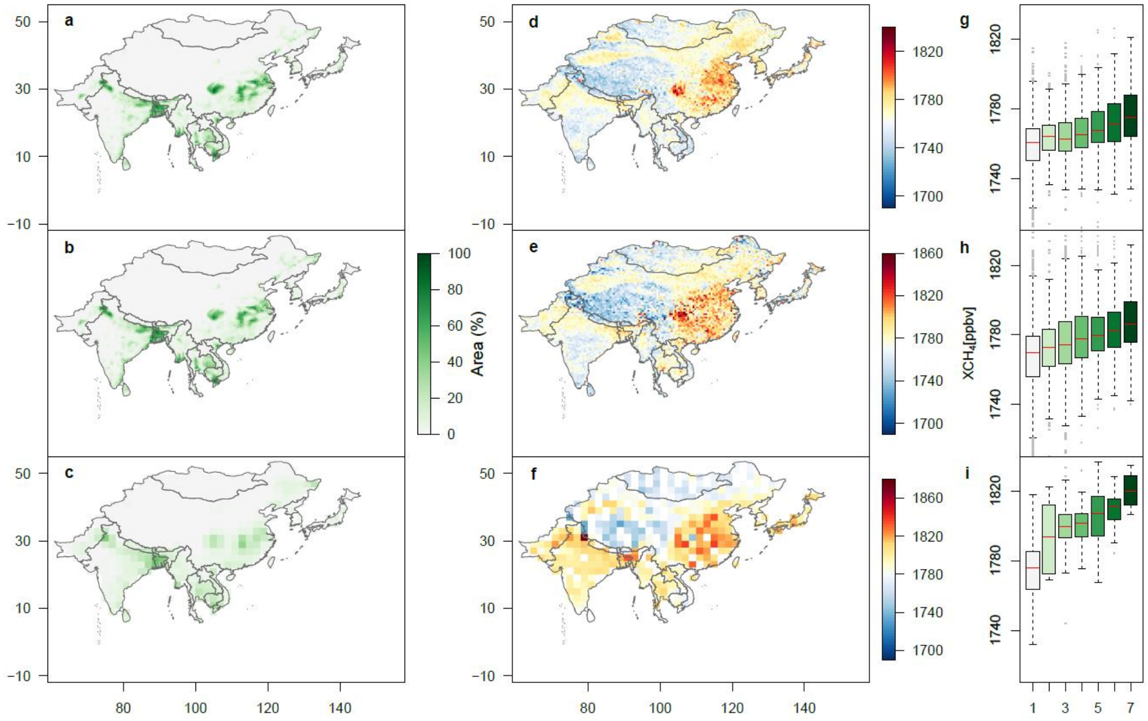

Supplementary Figure 10. Consistency of spatial distributions between paddy rice croplands and atmospheric methane concentration but for Indonesia and Malaysia. The three periods (2003-2005, 2007-2009, and 2011-2013) were selected to illustrate the spatial relationships between them for spring. **a-c** Spatial distributions of multi-year averaged area proportions of MODIS-based rice paddies for 2003–2005 with 0.5° (latitude/longitude) gridcells (**a**), 2007–2009 with 0.5°gridcells (**b**), and 2011–2013 with 2.0°gridcells (**c**). **d-f** Spatial distributions of multi-year averaged spring column-averaged concentration of CH_4 (XCH_4) from SCHAMACHY for 2003–2005 with 0.5° resolution (**d**), 2007–2009 with 0.5° resolution (**e**), and from TANSO-FTS for 2011–2013 with 2.0° resolution (**f**). **g-i** Multi-year averaged spring XCH_4 for different rice paddy area proportions for 2003–2005 (**g**), 2007–2009 (**h**), and 2011–2013 (**i**). The x-axis values in figure **g-i** represent levels of rice paddy area proportions in monsoon Asia, and 1–7

correspond to <0.5%, 0.5~1%, 1~5%, 5~10%, 10~20%, 20~40%, and >40% rice paddy area proportions, respectively.

Supplementary Figure 11

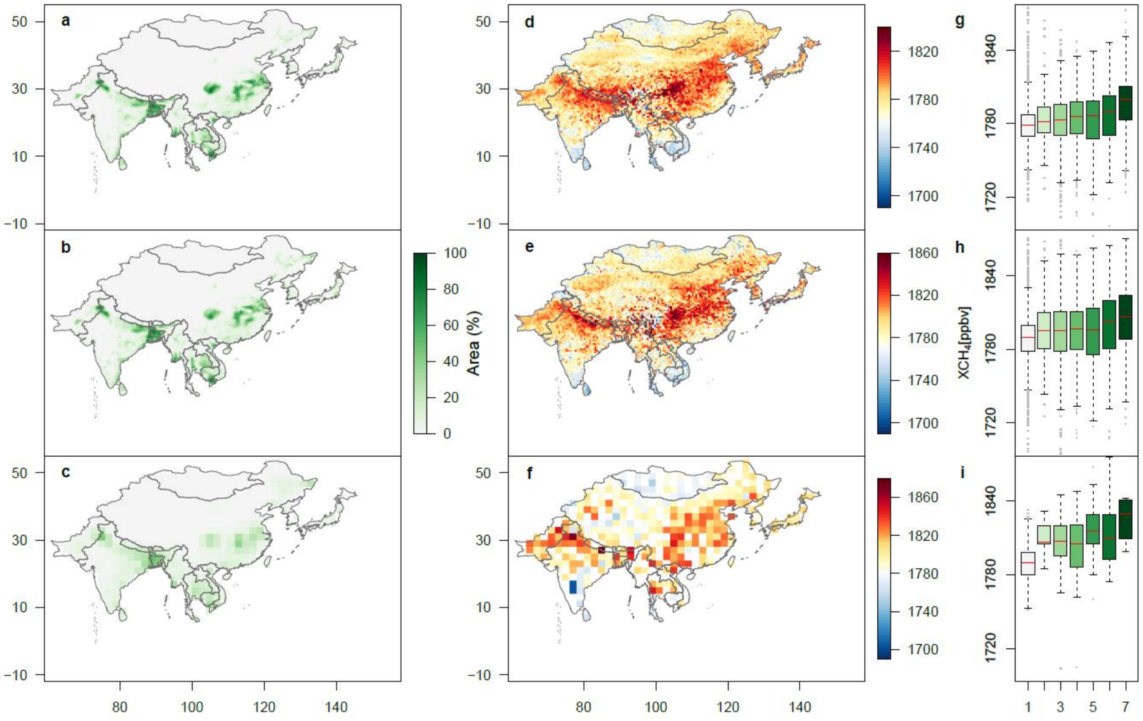

Supplementary Figure 11. Consistency of spatial distributions between paddy rice croplands and atmospheric methane concentration but for Indonesia and Malaysia. The three periods (2003-2005, 2007-2009, and 2011-2013) were selected to illustrate the spatial relationships between them for summer. **a-c** Spatial distributions of multi-year averaged area proportions of MODIS-based rice paddies for 2003–2005 with 0.5°gridcells (**a**), 2007–2009 with 0.5°gridcells (**b**), and 2011–2013 with 2.0°gridcells (**c**). **d-f** Spatial distributions of multi-year averaged summer column-averaged concentration of CH_4 (XCH_4) from SCHAMACHY for 2003–2005 with 0.5° resolution (**d**), 2007–2009 with 0.5° resolution (**e**), and from TANSO-FTS for 2011–2013 with 2.0° resolution (**f**). **g-i** Multi-year averaged summer XCH_4 for different rice paddy area proportions for 2003–2005 (**g**), 2007–2009 (**h**), and 2011–2013 (**i**). The x-axis values in figure **g-i** represent levels of rice paddy area proportions in monsoon Asia, and 1–7

correspond to <0.5%, 0.5~1%, 1~5%, 5~10%, 10~20%, 20~40%, and >40% rice paddy area proportions, respectively.

Supplementary Figure 12

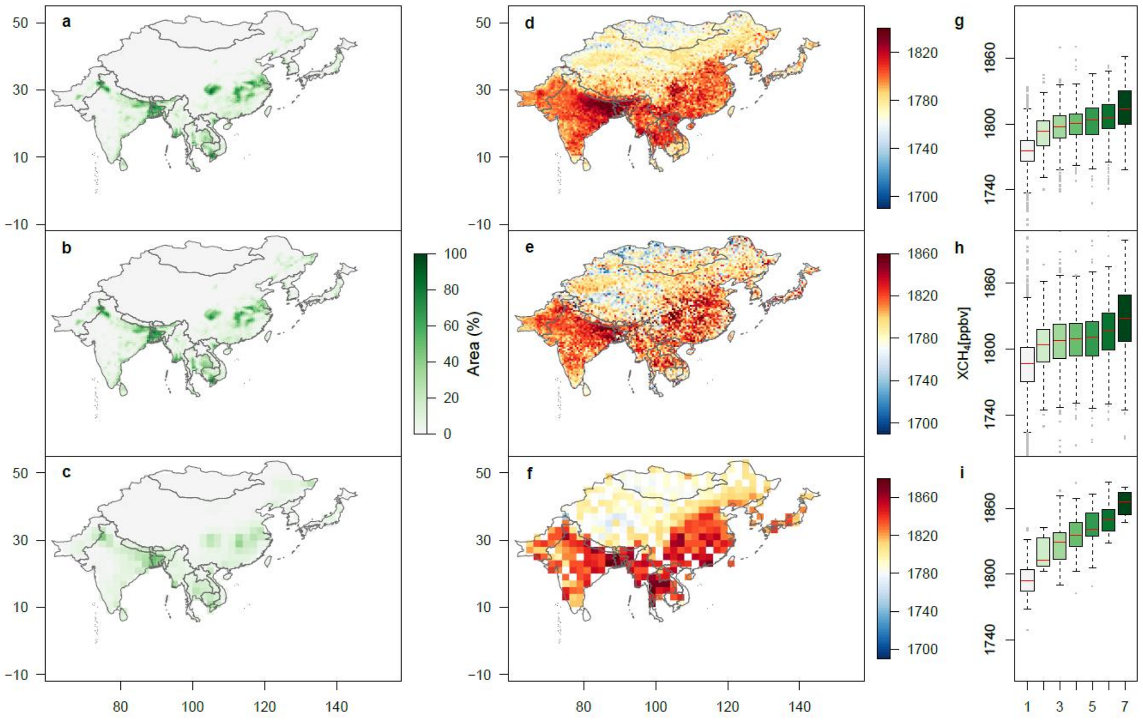

Supplementary Figure 12. Consistency of spatial distributions between paddy rice croplands and atmospheric methane concentration but for Indonesia and Malaysia. The three periods (2003-2005, 2007-2009, and 2011-2013) were selected to illustrate the spatial relationships between them for autumn. **a-c** Spatial distributions of multi-year averaged area proportions of MODIS-based rice paddies for 2003–2005 with 0.5°gridcells (**a**), 2007–2009 with 0.5°gridcells (**b**), and 2011–2013 with 2.0°gridcells (**c**). **d-f** Spatial distributions of multi-year averaged autumn column-averaged concentration of CH_4 (XCH_4) from SCHAMACHY for 2003–2005 with 0.5° resolution (**d**), 2007–2009 with 0.5° resolution (**e**), and from TANSO-FTS for 2011–2013 with 2.0° resolution (**f**). **g-i** Multi-year averaged autumn XCH_4 for different rice paddy area proportions for 2003–2005 (**g**), 2007–2009 (**h**), and 2011–2013 (**i**). The x-axis values in figure **g-i** represent levels of rice paddy area proportions in monsoon Asia, and 1–7

correspond to <0.5%, 0.5~1%, 1~5%, 5~10%, 10~20%, 20~40%, and >40% rice paddy area proportions, respectively.

Supplementary Figure 13

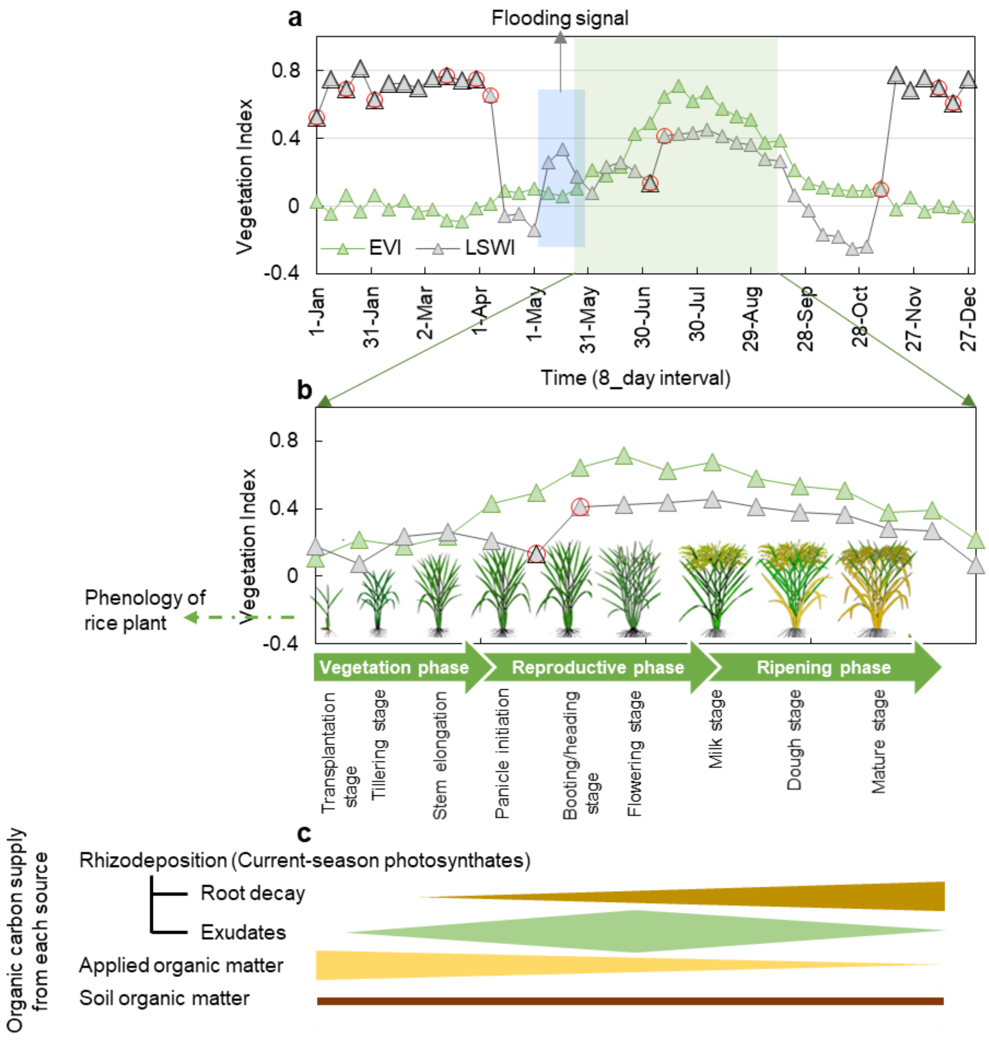

Supplementary Figure 13. The seasonal variations of rice growth. **a** The seasonal dynamics of the Enhanced Vegetation Index (EVI) and Land Surface Water Index (LSWI) from a rice paddy pixel with 500 m resolution in 2010 (location: 132.825 °E, 45.737 °N). The hollow triangular and round symbols in (a) mean the cloud and cloud shadow from the MODIS quality layer and blue band >= 0.2, respectively, which are labeled on the LSWI curves. **b** The schematic of relationships between paddy rice growth and vegetation indies (EVI and LSWI). **c** The schematic of supply rates of organic carbon

from different sources as substrates for methane production with rice growth, which is modified from Hayashi et al. [1]. Applied organic matter includes rice straw, stubble, and roots produced in the former season, organic fertilizers, and so on.

Supplementary Figure 14

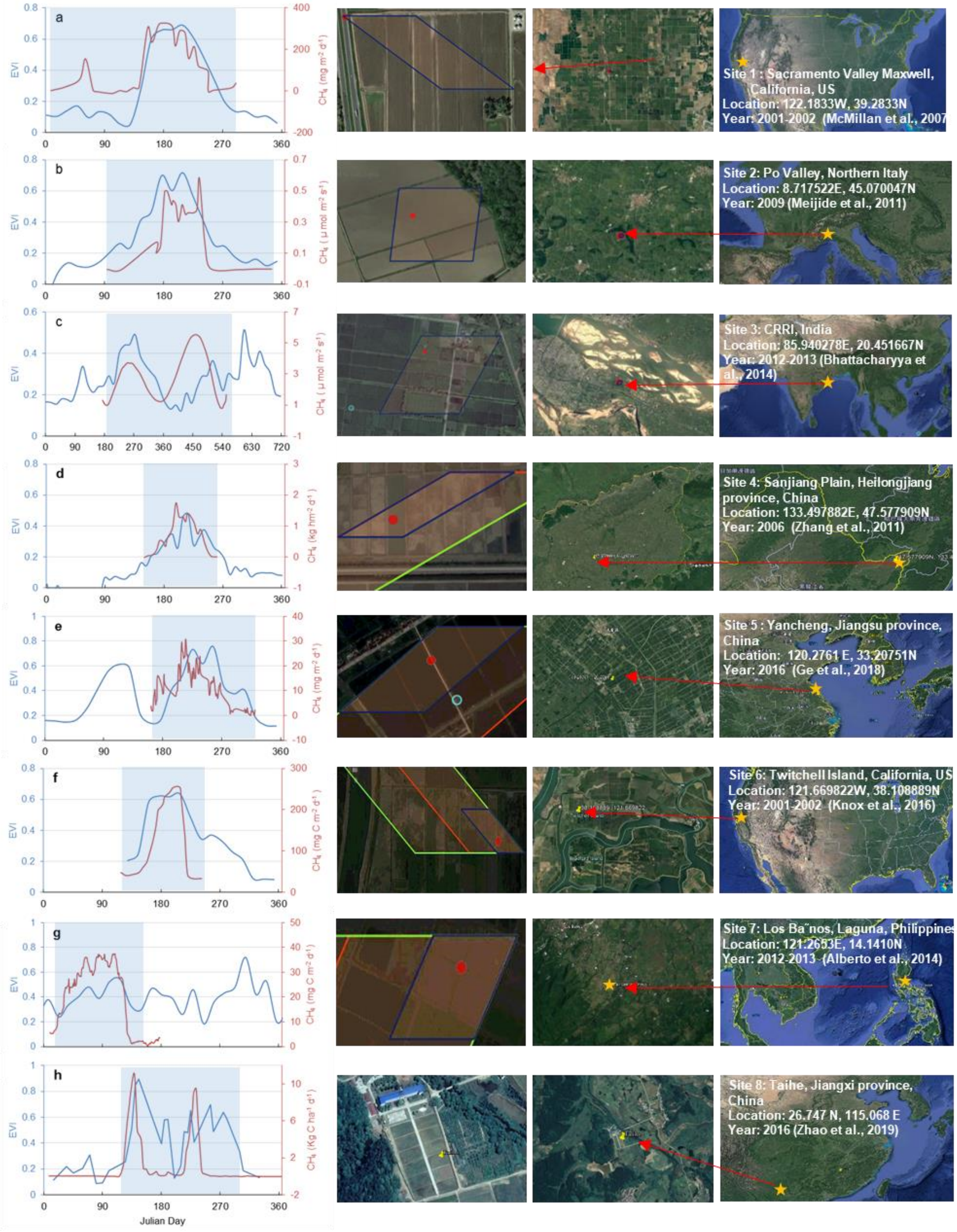

Supplementary Figure 14. Seasonal dynamics of methane emissions from rice paddies and enhanced vegetation index (EVI) of rice paddies for eight sites. **a**-**h** Time series of methane fluxes with red lines and EVI with blue lines of rice paddies over **a**) Sacramento

Valley Maxwell, California, US (122.1833W, 39.2833N) for 2001-2002 [2], **b**) Po Valley, Northern Italy (8.717522E, 45.070047N) for 2009 [3], **c**) CRRI, India (85.940278E, 20.451667N) for 2012-2013 [4], **d**) Sanjiang Plain, Heilongjiang province, China (133.497882E, 47.577909N) for 2006 [5], **e**) Yancheng, Jiangsu province, China (120.2761 E, 33.20751N) for 2016 [6], **f**) Twitchell Island, California, US (121.669822W, 38.108889N) for 2001-2002 [7], **g**) Los Ba˜nos, Laguna, Philippines (121.2653E, 14.1410N) for 2012-2013 [8], and **h**) Taihe, Jiangxi province, China (26.747 N, 115.068 E) for 2016 [9]. The right figures show the detailed geographic locations of those sites. The methane fluxes data are from published papers [2-9]. The EVI data for individual flux tower sites are MODIS 16-day vegetation indices with 250 m (MOD13Q1), downloaded from the data portal at the University of Oklahoma (http://www.eomf.ou.edu/visualization/) according to the detailed geographic information mentioned in corresponding published papers. The timings of seasonal peaks of paddy rice growth and XCH_4 were consistent among the eight sites (RMSE = 14 day), and the start and end dates of rice plant growth and CH_4 emission were also consistent, which suggested that the developmental stages of paddy rice plants affected the seasonality of CH_4 emission.

Supplementary Figure 15

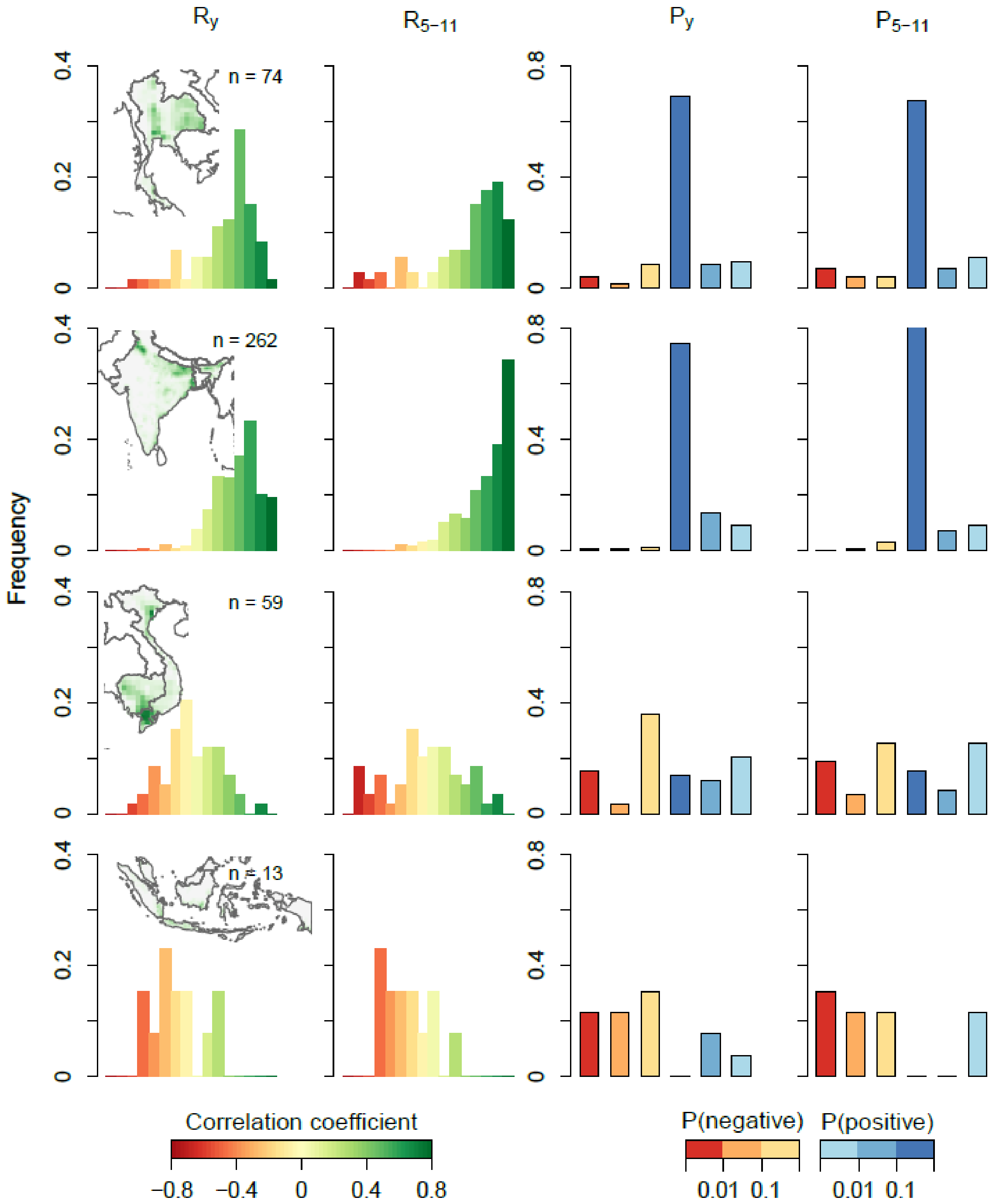

Supplementary Figure 15. Frequency of the correlation between seasonal atmospheric methane concentration and rice growth in the regions with different cropping systems in the period 2003-2005. The four regions are Thailand with single rice in the east part (1st

row), India with winter wheat and rice (2nd row), Cambodia and Vietnam with multiple cropping systems (3rd row), and Indonesia and Malaysia with much cloud (4th row). The first and second columns are the frequency of Pearson's correlation coefficients between XCH_4 and EVI for the whole year (R_y) and summer-fall season from May to November (R_{5-11}) in the four regions, respectively. The third and fourth columns are the frequency of significance levels of Pearson's correlation between the two for the whole year (P_y) and summer-fall season from May to November (P_{5-11}) in these regions, respectively. n means the number of gridcells with valid value.

Supplementary Figure 16

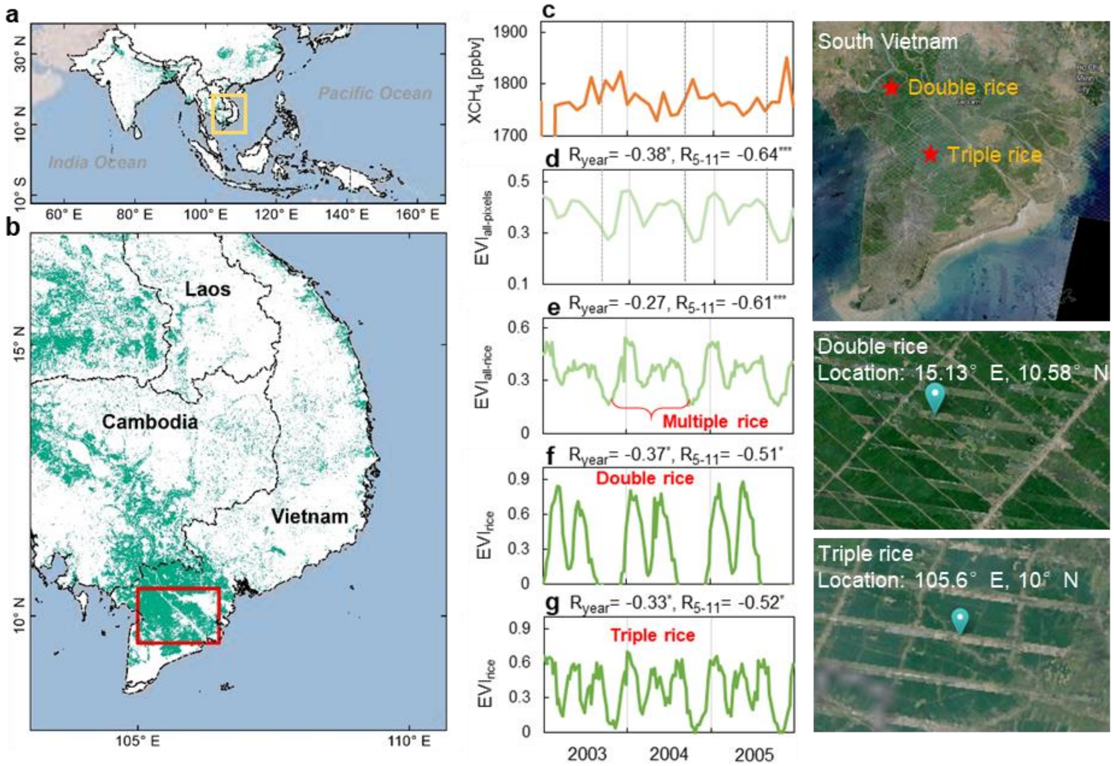

Supplementary Figure 16. Seasonal dynamics of atmospheric methane concentration and rice growth in the Mekong Delta of Vietnam. The column-averaged methane concentration (XCH_4) and the enhanced vegetation index (EVI) were analyzed in the region with dense rice paddies. **a** The location of the Mekong Delta in Monsoon Asia. **b** Spatial distribution of rice paddies retrieved from MODIS data with 500 m resolution in 2004. **c** Time series of mean monthly SCIAMACHY CH_4 column volume mixing ratios (VMRs, in parts per billion) over this region labeled in **b** with red polygon. **d** Time series of monthly EVI for all pixels within 0.5° gridcells ($EVI_{all\text{-}pixels}$) over this region. **e** Time series of 8-day EVI values for all 500 m rice paddy pixels within the 0.5 ° gridcells ($EVI_{all\text{-}rice}$). **f-g** Time series of 8-day EVI values for one rice paddy pixel with double (**f**) and triple (**g**) rice and 500 m resolution (EVI_{rice}). The right images are the landscapes for

the Mekong Delta and rice paddy pixels (**f-g**) with double and triple rice in the extent of 500 m by 500 m from Google Earth, and the locations of these two pixels are labeled on the top one of the right images with red star symbol following Ref Son et al. [10]. R_{year} and $R_{5\text{-}11}$ offer the Pearson's correlation coefficients between XCH_4 and EVI for the whole year and summer-fall season from May to November during the corresponding period labeled in the x-axis of **d-g**. ***P<0.01; *P<0.10. Correlation coefficients without asterisk are insignificant (P>0.10).

Supplementary Figure 17

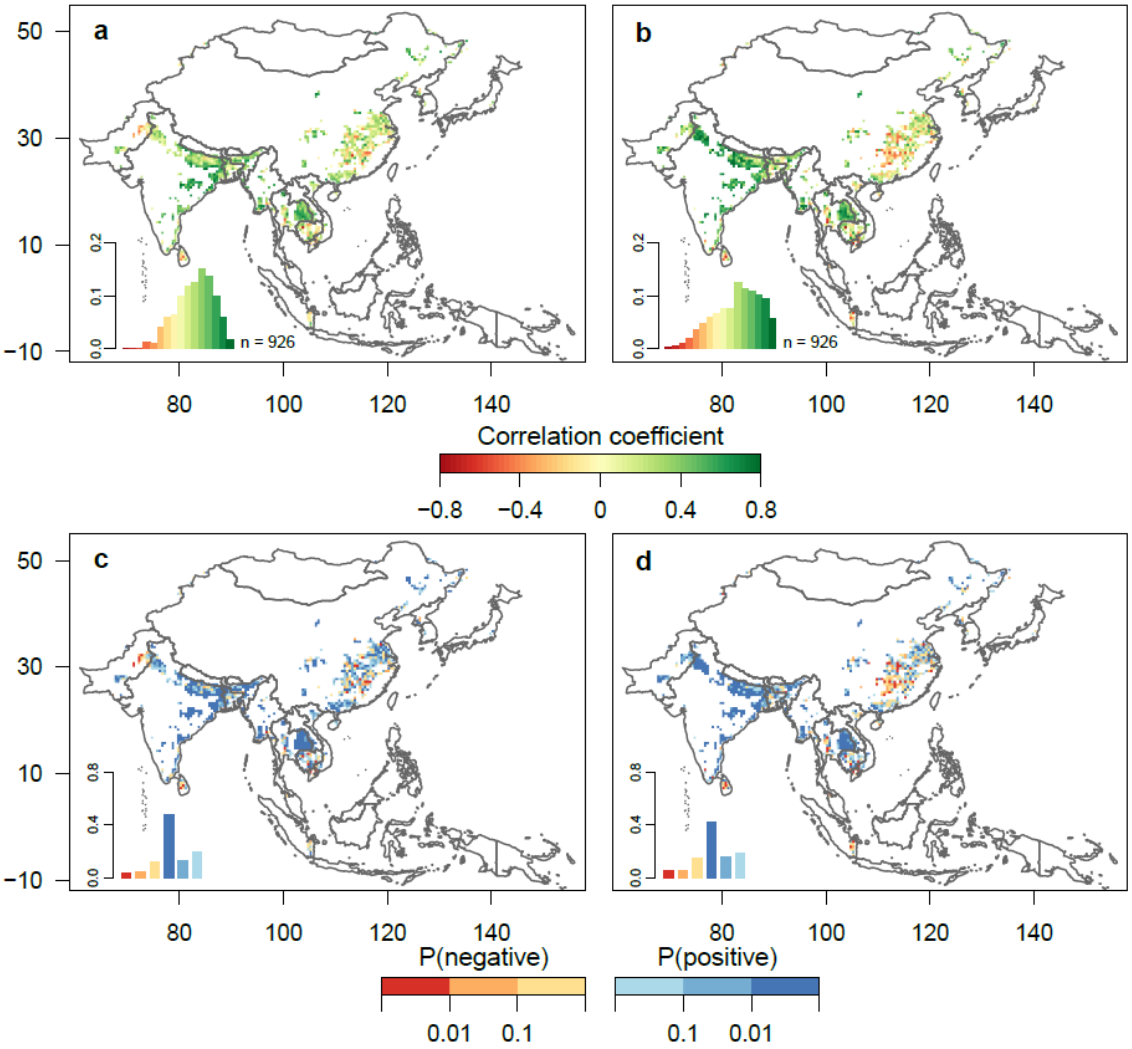

Supplementary Figure 17. Correlations between monthly atmospheric methane concentration from SCIAMACHY and enhanced vegetation index (EVI) in the dense rice paddies of monsoon Asia during 2005–2007. **a**, **b** The spatial distributions of Pearson's correlation coefficients between the two sources for the whole year and summer-fall season from May to November, respectively. **c**, **d** The spatial distributions of significance levels of Pearson's correlation between the two for the whole year and summer-fall season from May to November, respectively. The region with a multi-year averaged rice

paddy gridcells with area proportion larger than 10% during 2005–2007 was considered here. The insets in a-d are the corresponding frequency diagrams of Figure **a-d**.

Supplementary Figure 18

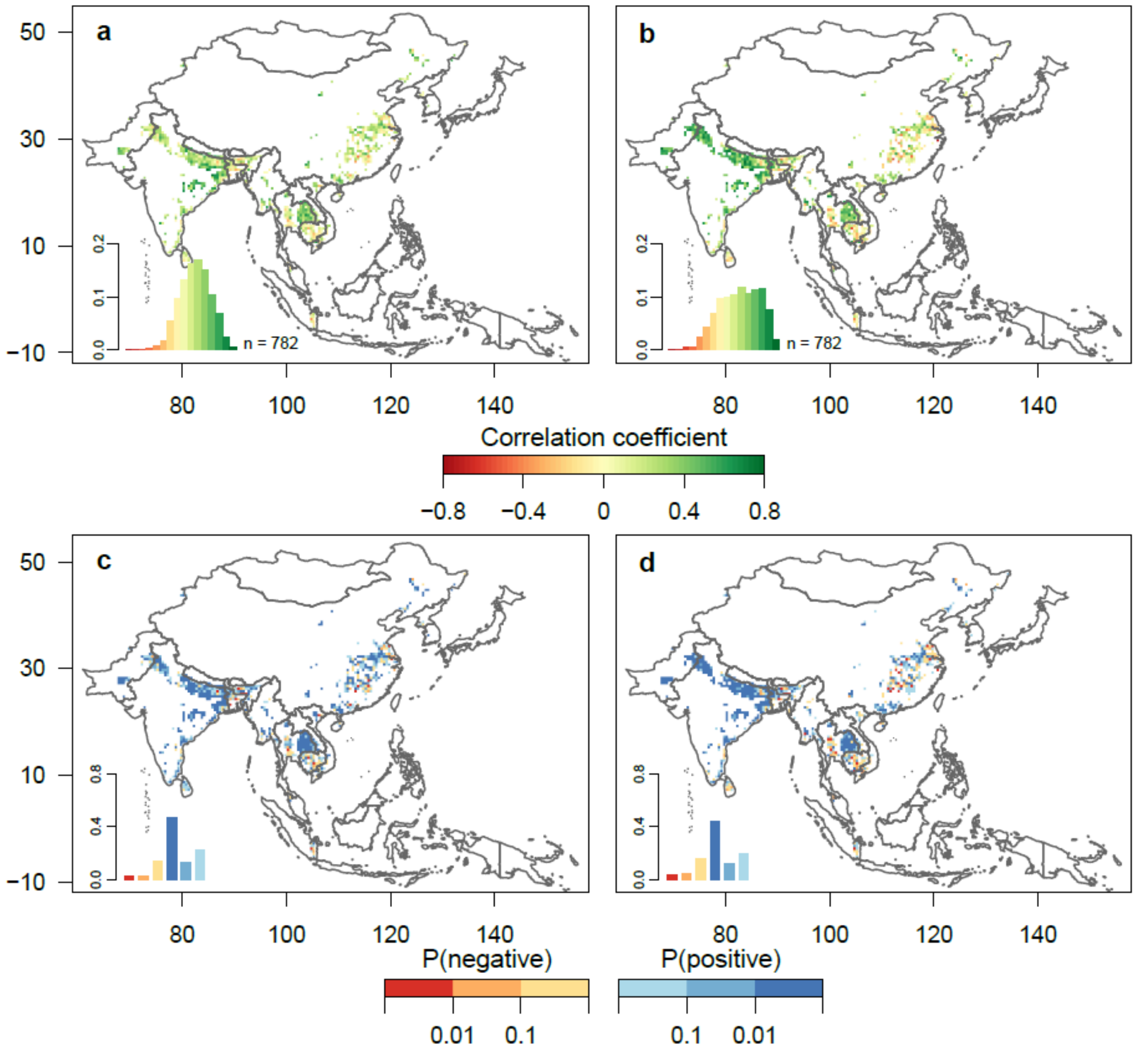

Supplementary Figure 18. Correlations between monthly atmospheric methane concentration from SCIAMACHY and enhanced vegetation index (EVI) in the dense rice paddies of monsoon Asia during 2007–2009. **a**, **b** The spatial distributions of Pearson's correlation coefficients between the two sources for the whole year and summer-fall season from May to November, respectively. **c**, **d** The spatial distributions of significance levels of Pearson's correlation between the two for the whole year and summer-fall season from May to November, respectively. The region with a multi-year averaged rice

paddy gridcells with area proportion larger than 10% during 2007–2009 was considered here. The insets in a-d are the corresponding frequency diagrams of Figure **a-d**.

Supplementary Figure 19

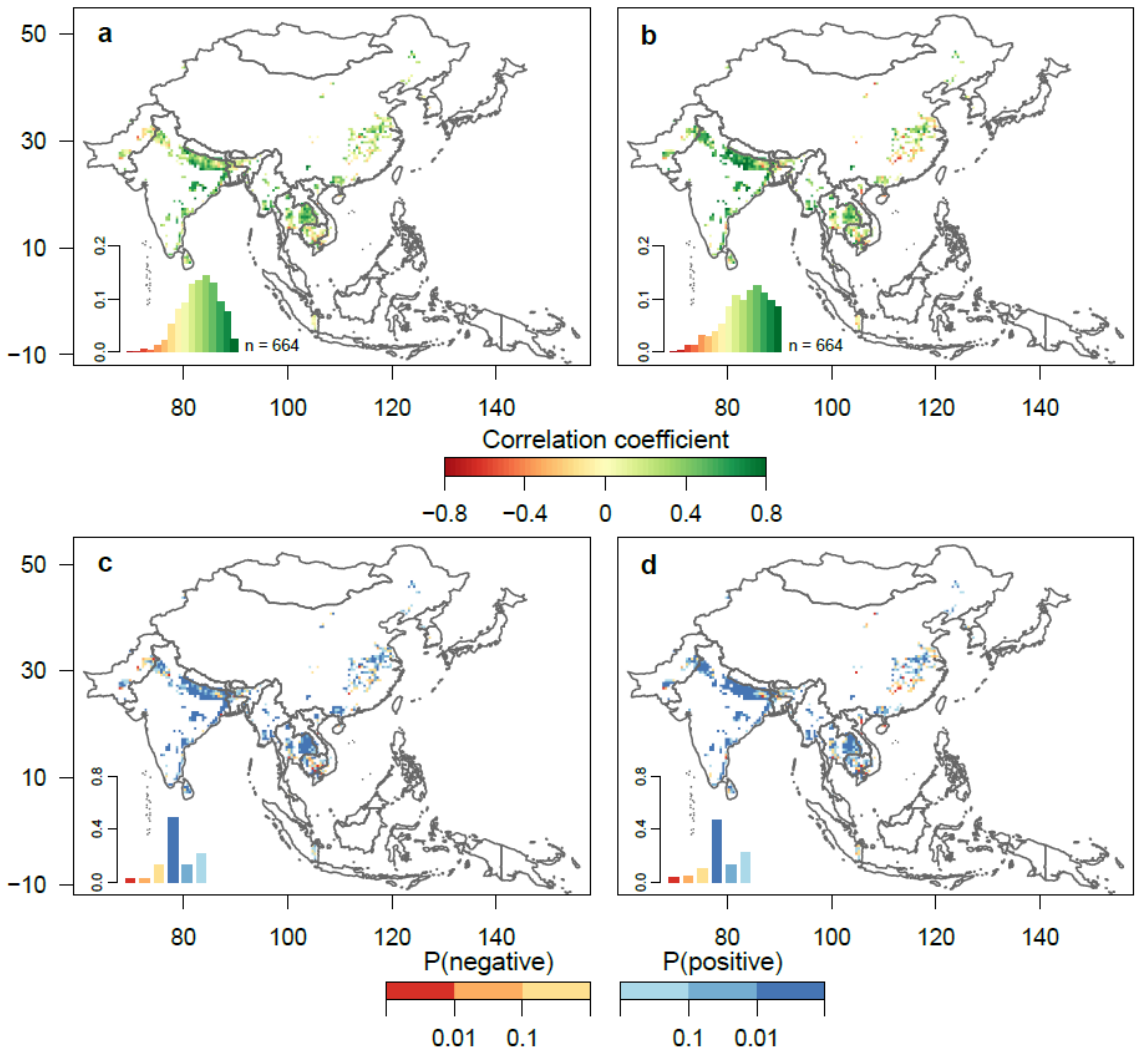

Supplementary Figure 19. Correlations between monthly atmospheric methane concentration from SCIAMACHY and enhanced vegetation index (EVI) in the dense rice paddies of monsoon Asia during 2009–2011. **a**, **b** The spatial distributions of Pearson's correlation coefficients between the two sources for the whole year and summer-fall season from May to November, respectively. **c**, **d** The spatial distributions of significance levels of Pearson's correlation between the two for the whole year and summer-fall season from May to November, respectively. The region with a multi-year averaged rice

paddy gridcells with area proportion larger than 10% during 2009–2011 was considered here. The insets in a-d are the corresponding frequency diagrams of Figure **a-d**.

Supplementary Figure 20

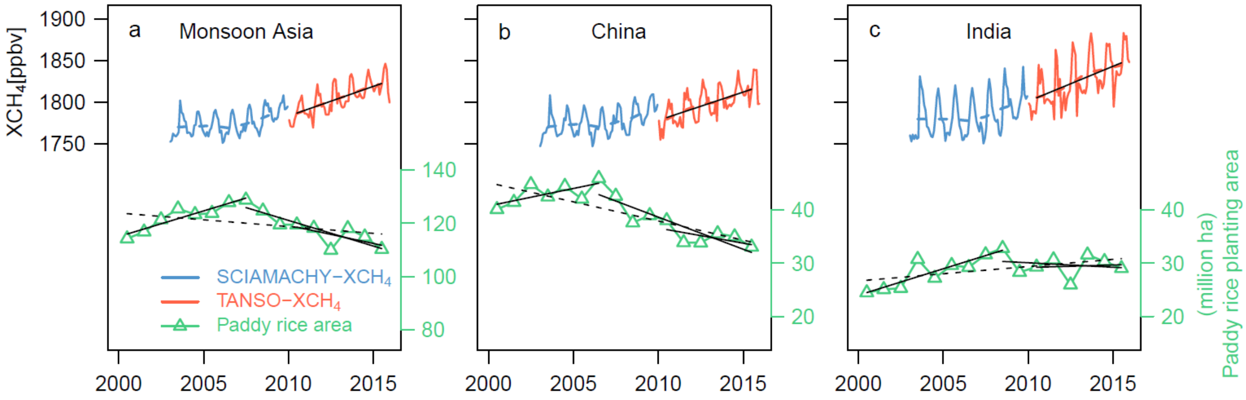

2000−2015: −0.51 (P=0.10)
2000−2007: 1.93 (P=0.00)
2007−2015: −1.93 (P=0.00)
2010−2015: −1.36 (P=0.20)
2010−2015: 7.14 (P=0.00, XCH4)

2000−2015: −0.72 (P=0.00)
2000−2006: 0.66 (P=0.09)
2006−2015: −1.21 (P=0.00)
2010−2015: −0.57 (P=0.21)
2010−2015: 6.81 (P=0.00, XCH4)

2000−2015: 0.27 (P=0.05)
2000−2008: 0.99 (P=0.00)
2008−2015: −0.17 (P=0.64)
2010−2015: 0.09 (P=0.87)
2010−2015: 8.42 (P=0.00, XCH4)

Supplementary Figure 20. Temporal (seasonal and interannual) dynamics of atmospheric column-averaged methane concentration (XCH_4) and interannual variations of MODIS-based rice paddy areas during 2000–2015 in monsoon Asia (**a**), China (**b**), and India (**c**). The atmospheric methane concentration data were calculated based on the spatial domains of monsoon Asia, China, and India, including paddy rice area and no paddy rice area. The curves above are time series of monthly SCIAMACHY CH_4 column volume mixing ratios (VMRs, in parts per billion) during 2003–2009 and monthly TANSO CH_4 column VMRs during 2010–2015. The XCH_4 outliers in winter have been removed. The blue and red dashed lines are averaged annual values of SCIAMACHY XCH_4 and TANSO XCH_4, respectively. The black lines and black dashed lines below indicate trends of paddy rice croplands for different periods in monsoon Asia, China, and India. The linear trend and its significance level, P, for different periods are shown below the panels; the formulas in black color are for paddy rice planting area, and the formula in red is for XCH_4.

Supplementary Figure 21

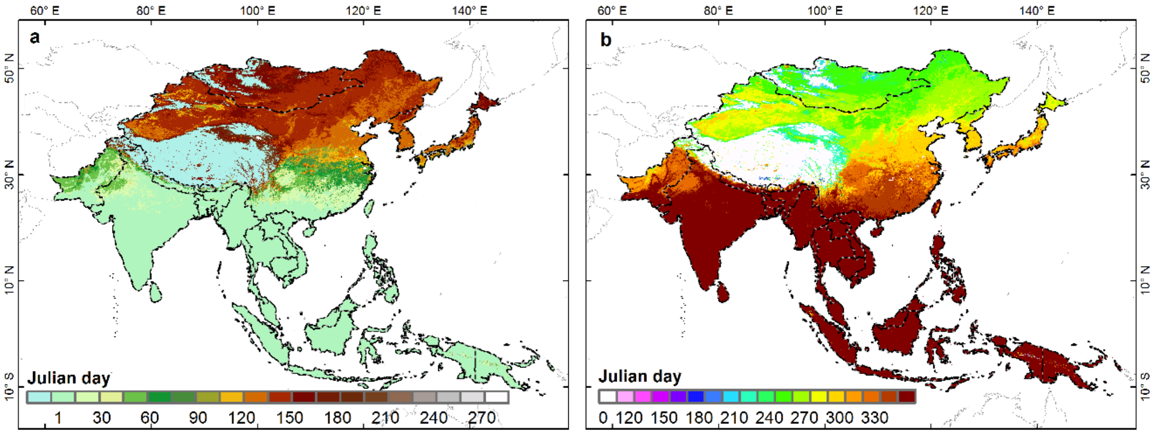

Supplementary Figure 21. Spatial patterns of start date (**a**) and end date (**b**) of nighttime land surface temperature higher than 5 °C in 2010 in monsoon Asia.

Supplementary Figure 22

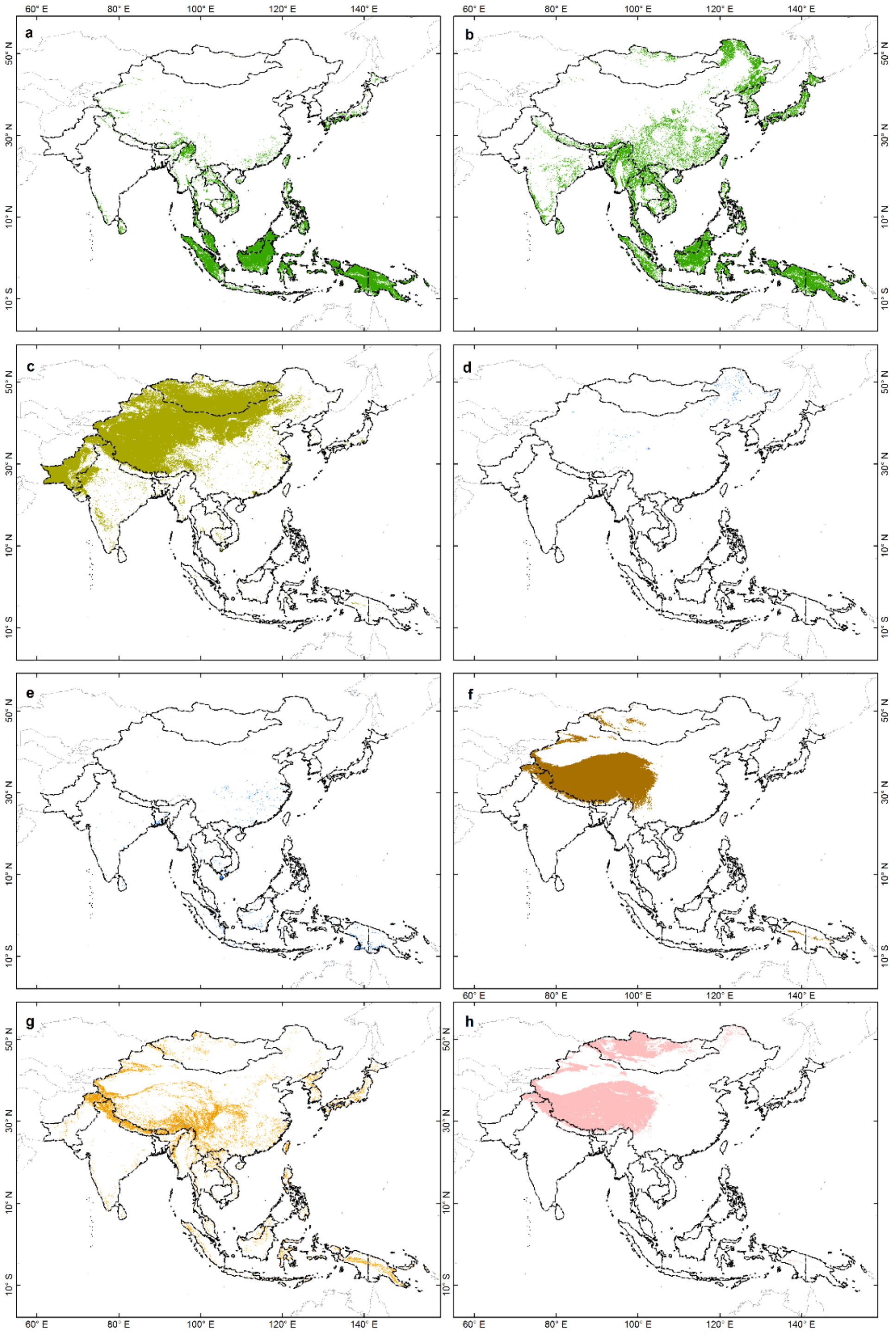

Supplementary Figure 22. Spatial patterns of non-cropland masks for paddy rice mapping in monsoon Asia, including evergreen vegetation (**a**), the Phased Array Type L-band

Synthetic Aperture Radar (PALSAR)-based forests (**b**), MODIS-based sparsely vegetated lands in 2010 (**c**), the National Land Cover and Land Use Dataset (NLCD)-based wetlands in China (**d**), the MODIS land cover type product (MCD12Q1)-based wetlands in 2010 (**e**), the Digital Elevation Model (DEM)-based elevation mask (**f**), DEM-based slope mask (**g**), and the temperature-based mask in 2010 (**h**).

Supplementary Figure 23

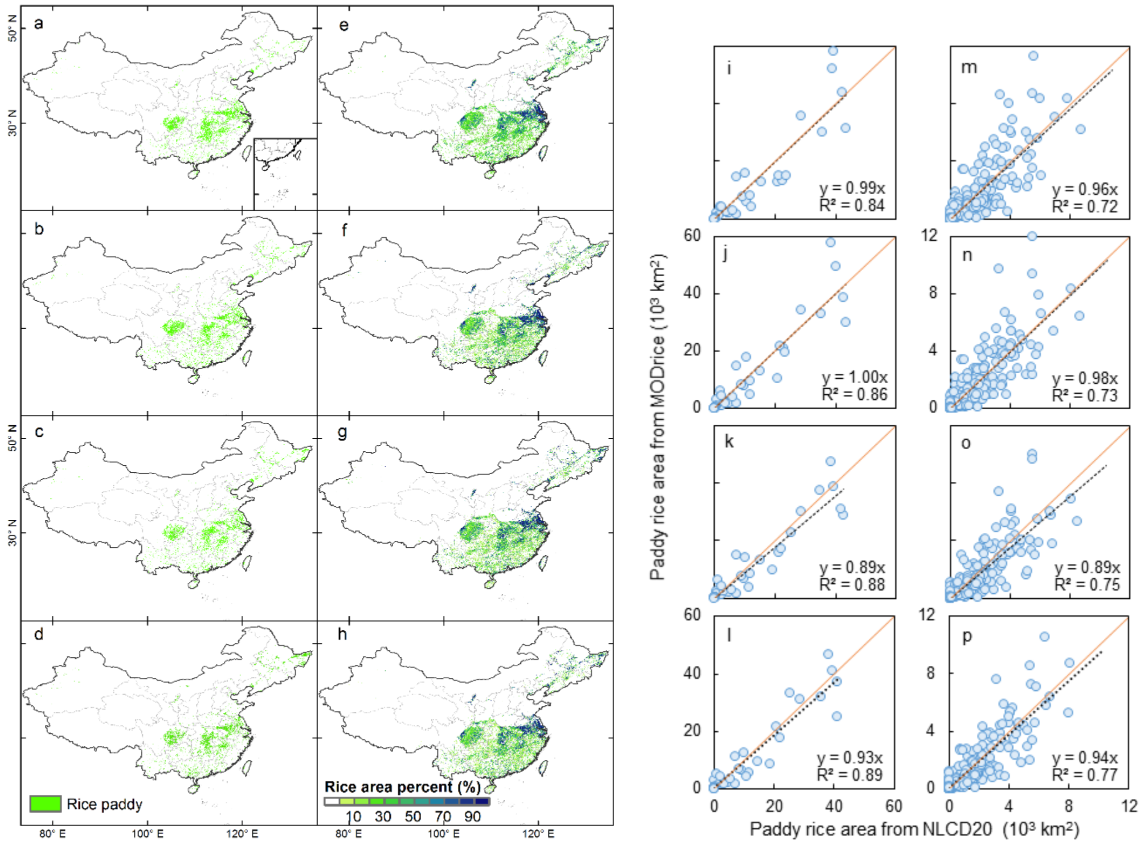

Supplementary Figure 23. The comparisons of MODIS-based paddy rice maps with National Land Cover and Land Use Dataset (NLCD)-based paddy rice maps in China in 2000, 2005, 2008, and 2010. **a-d** The spatial distributions of MODIS-based rice paddy in China in 2000, 2005, 2008, and 2010, respectively. **e-h** The spatial distributions of NLCD-based rice paddy in China in 2000, 2005, 2008, and 2010, respectively. **i-l** The scatter plots for the comparison between MODIS-based and NLCD-based rice paddy area at provincial level in 2000, 2005, 2008, and 2010, respectively. **m-p** The scatter plots for the comparison between MODIS-based and NLCD-based rice paddy area at prefectural level in 2000, 2005, 2008, and 2010, respectively.

Supplementary Figure 24

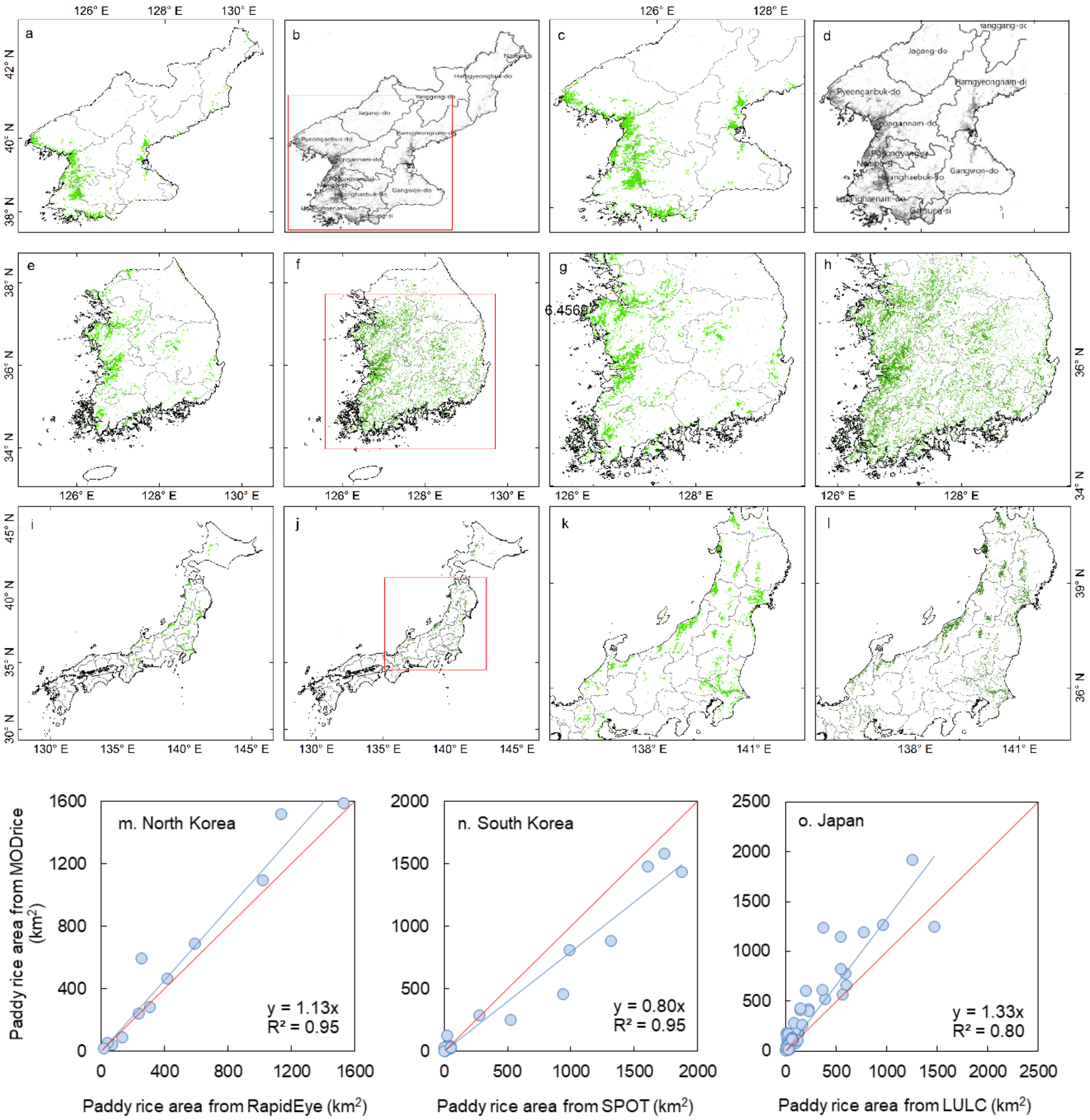

Supplementary Figure 24. The comparisons of MODIS-based paddy rice maps in 2010 with existing land use maps in North Korea (**a-d**), South Korea (**e-h**), and Japan (**i-l**). **a**, **e**, **i** The spatial distributions of MODIS-based rice paddy in North Korea, South Korea, and Japan in 2010, respectively. **b**, **f**, **j** The spatial distributions of RapidEye-based rice paddy in North Korea in 2010 from Hong et al. [11], OECD-derived paddy rice map in South Korea in 2008, and AVNIR-2-based rice paddy in Japan around 2009, respectively. **c/g/k**,

d/h/l The case regions from the MODIS-based paddy rice maps and existing products, respectively. **m**, **n**, **o** The scatter plot diagrams for comparison between MODIS-based paddy rice maps and existing land use maps at provincial level in North Korea, South Korea, and Japan, respectively. For more details about the existing land use products of different countries here, see Supplementary Note 3 for details.

Supplementary Figure 25

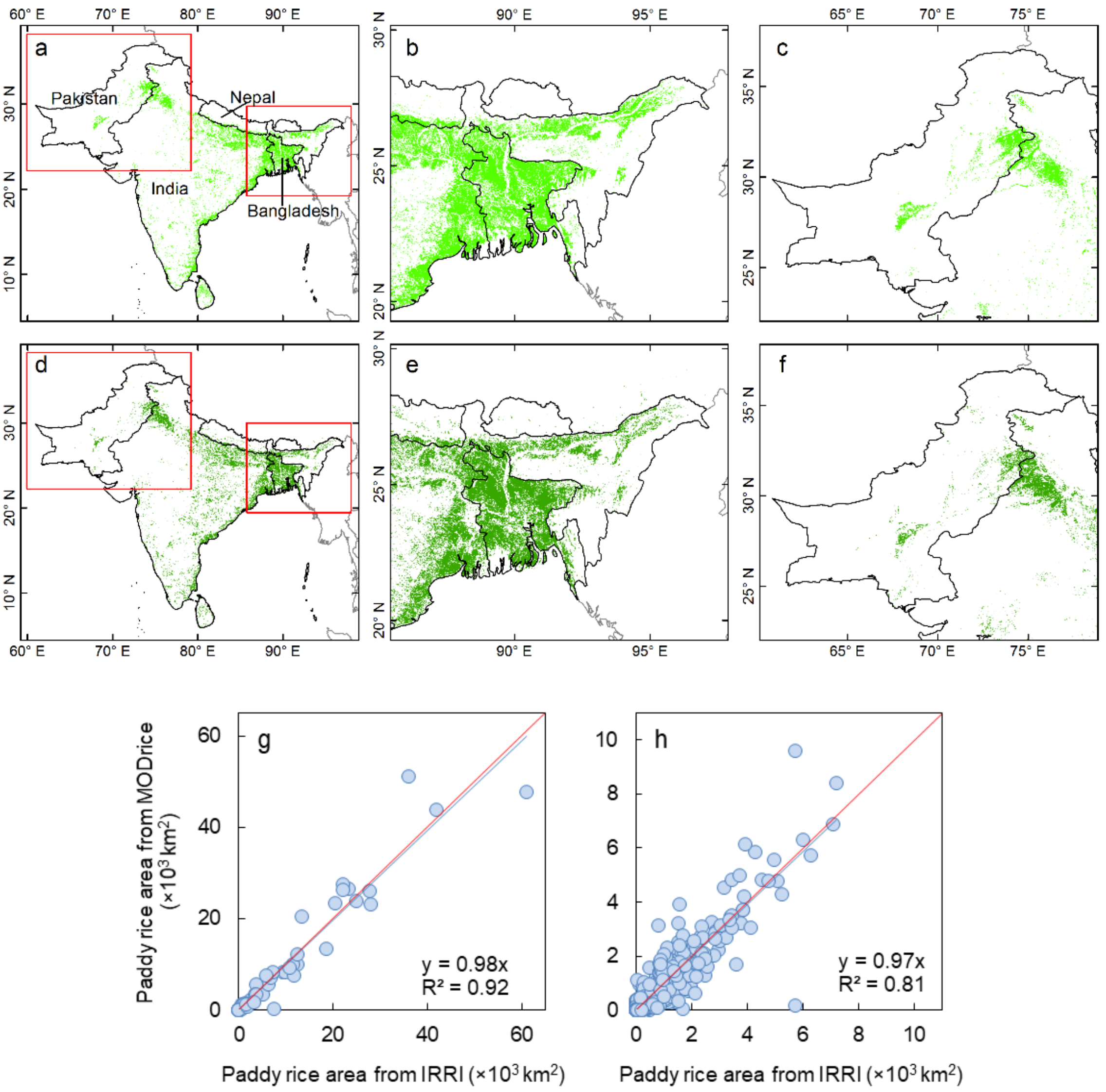

Supplementary Figure 25. The comparisons of MODIS-based paddy rice map (**a**) with the International Rice Research Institute (IRRI)-based paddy rice map (**d**) in South Asia during 2000–2001, including India, Bangladesh, Pakistan, and Nepal. **b**/**c**, **e**/**f** The case regions from the MODIS-based paddy rice maps and IRRI-based paddy rice maps, respectively. **g**, **h** The scatter plots for the comparisons between MODIS-based and IRRI-based rice paddy areas at provincial and prefectural level in South Asia during 2000–2001, respectively.

Supplementary Figure 26

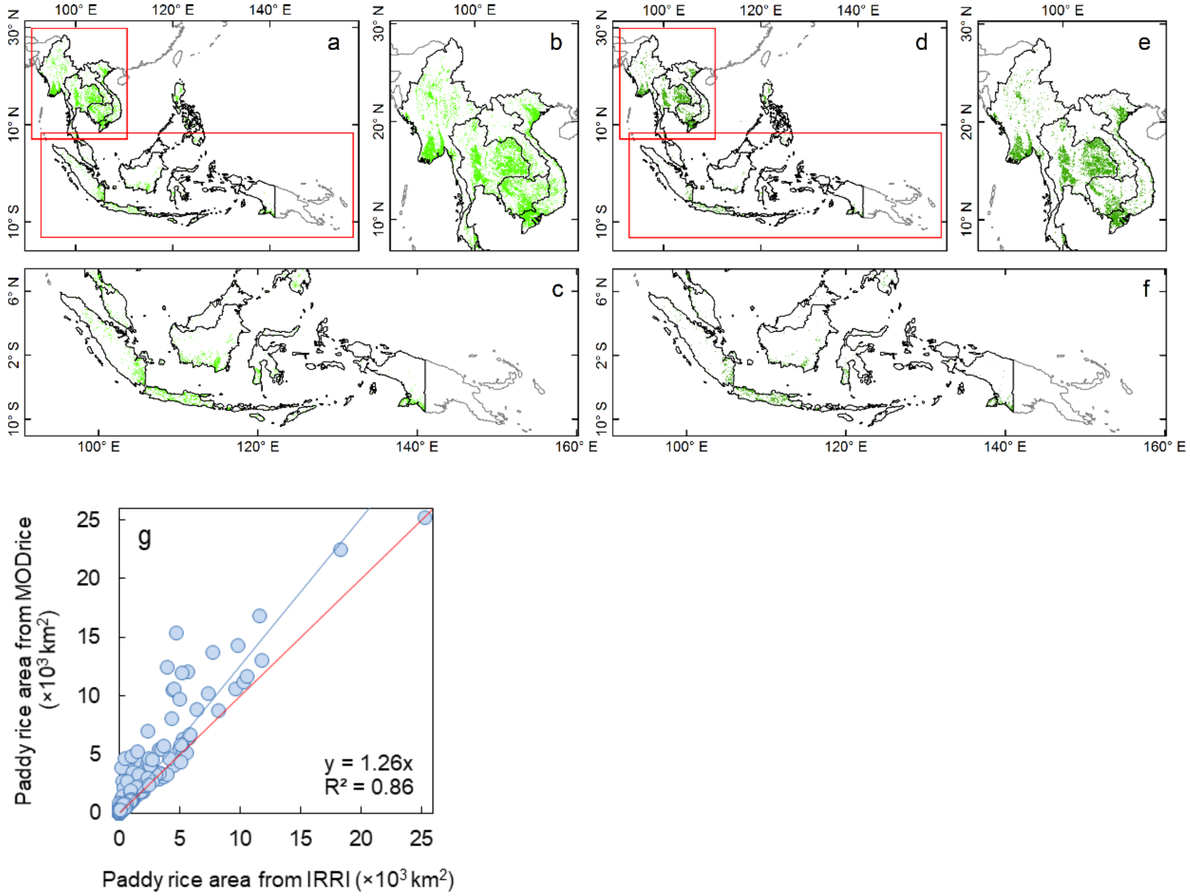

Supplementary Figure 26. The comparisons of MODIS-based paddy rice map (**a**) with the International Rice Research Institute (IRRI)-based paddy rice map (**d**) in Southeast Asia during the period of 2000–2012. **b**/**c**, **e**/**f** The case regions from the MODIS-based paddy rice maps and IRRI-based paddy rice maps, respectively. **g** The scatter plot for the comparison between MODIS-based and IRRI-based rice paddy area at provincial level in Southeast Asia for the period of 2000–2012.

Supplementary Figure 27

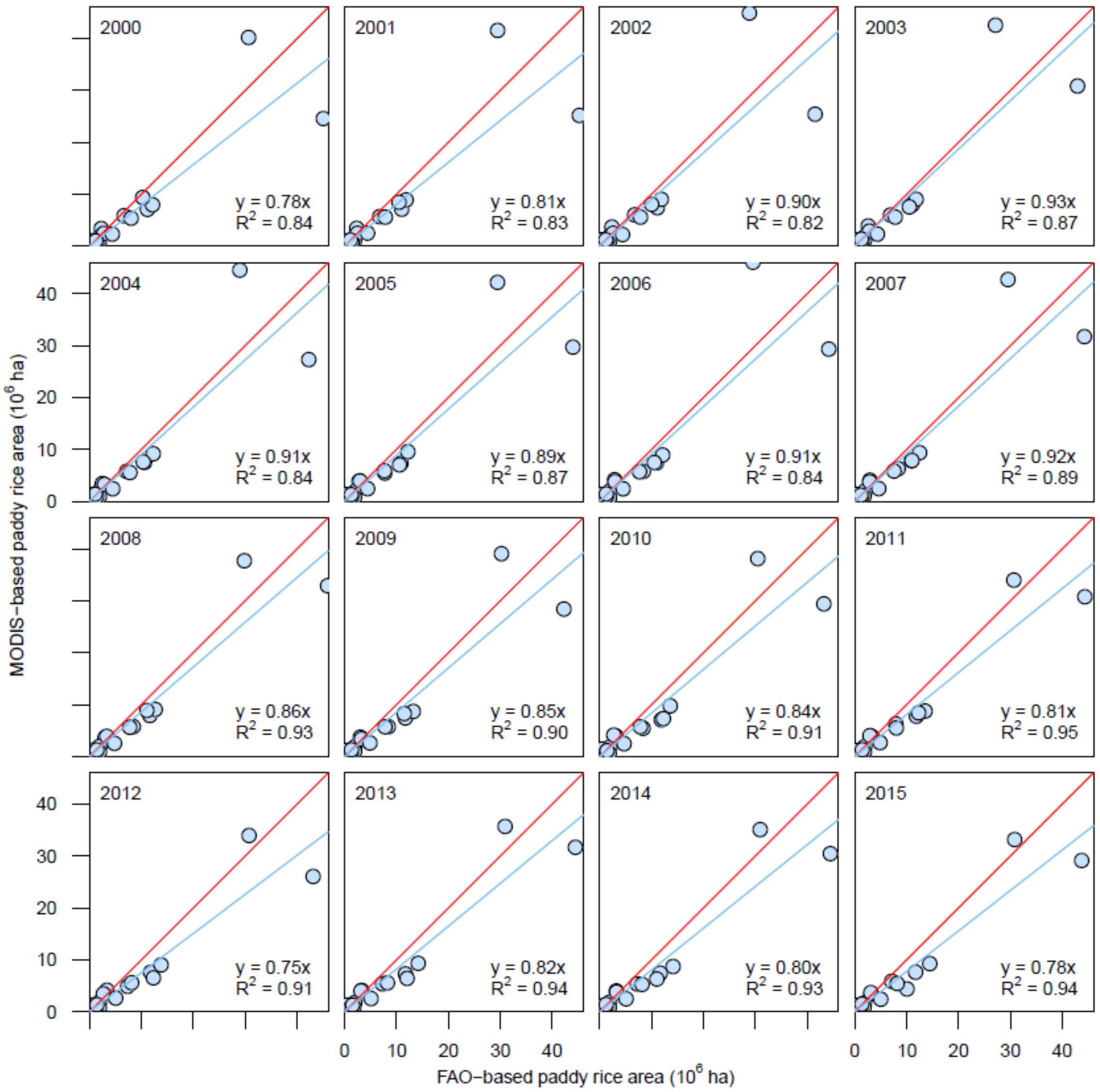

Supplementary Figure 27. The scatter plots of annual MODIS-based rice paddy areas and FAO-based rice paddy areas at national level from 2000 to 2015 for the countries dominated by rice paddy in monsoon Asia.

Supplementary Figure 28

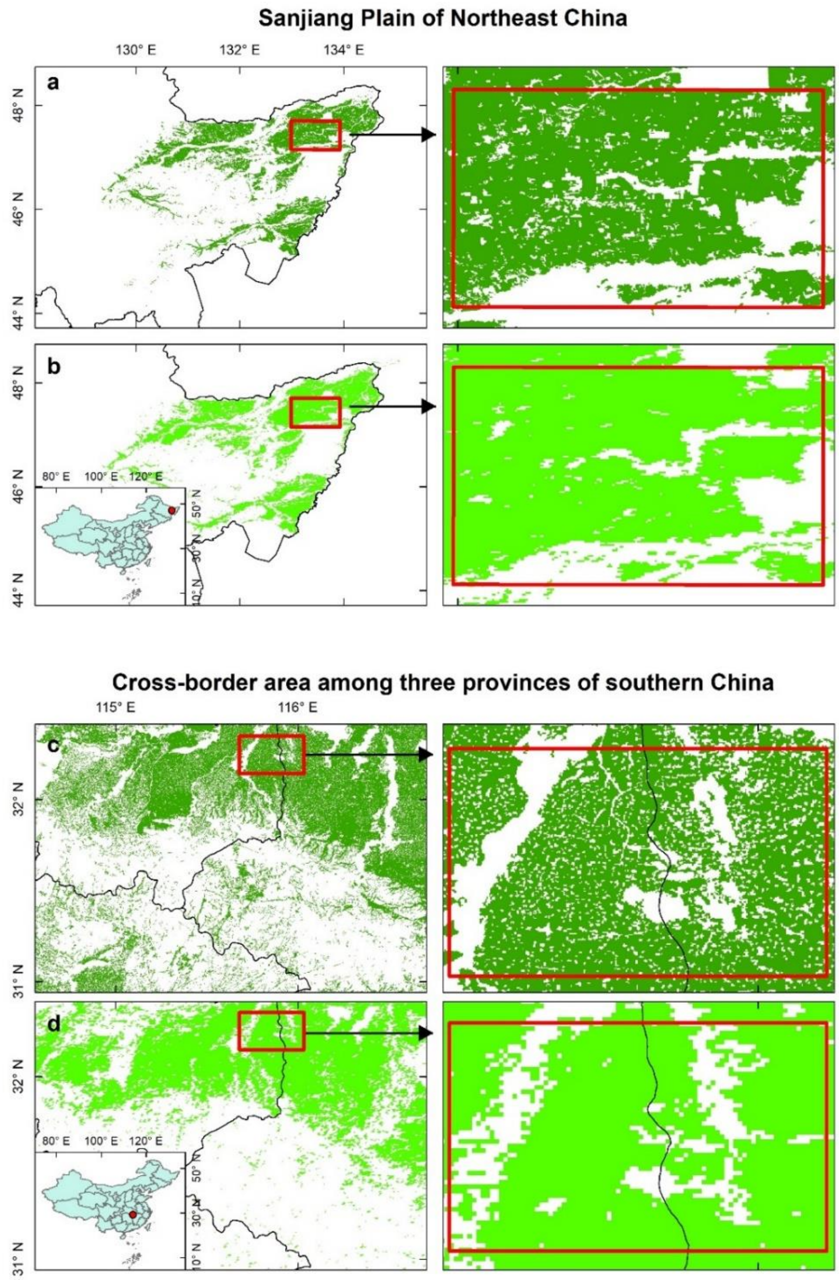

Supplementary Figure 28. Pixel-level comparisons between higher resolution imagery-based paddy rice maps (**a**, **c**) from National Land Cover Database (NLCD) with MODIS-based paddy rice maps (**b**, **d**) in this study for two case regions in 2015. **a**, **b** The Sanjiang Plain of Northeast China, where paddy rice fields and natural wetlands are mixed; **c**, **d** A

typical mountainous region in the cross-border area among three provinces of southern China (Hubei, Anhui, and Henan provinces). The right panels show more details within the red rectangle polygons in left maps. The insets in **b** and **d** show the detailed locations of these two case regions in China.

Supplementary Figure 29

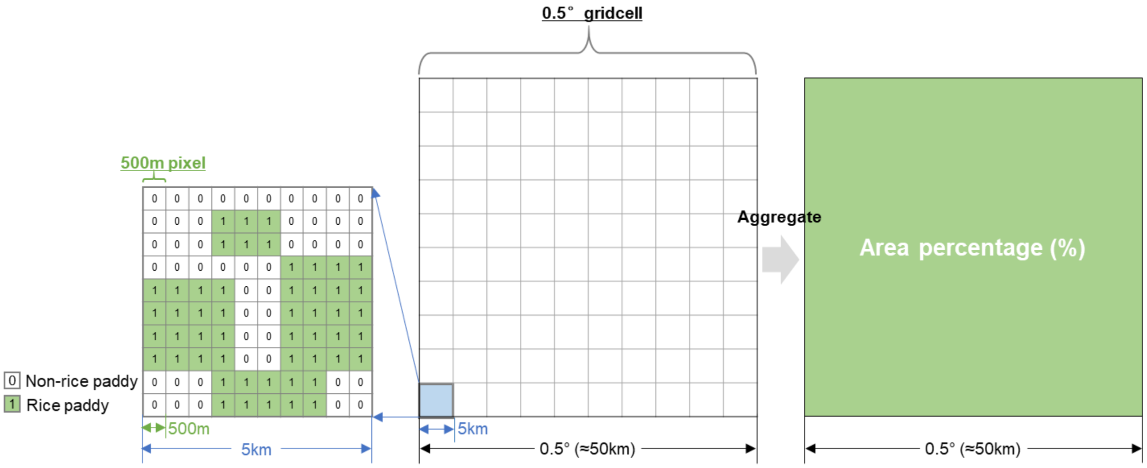

Supplementary Figure 29. Aggregation of 500 m paddy rice maps into 0.5° gridcells with rice paddy area percentages. The central figure represents a gridcell with 0.5° resolution; the left figure shows the zoom-in grids of the 500 m rice paddy and non-rice paddy pixels using a 5 km window as an example; the right figure shows the aggregated grid with area percentage information that counts all the paddy rice pixels in the central 0.5° gridcell.

Supplementary Figure 30

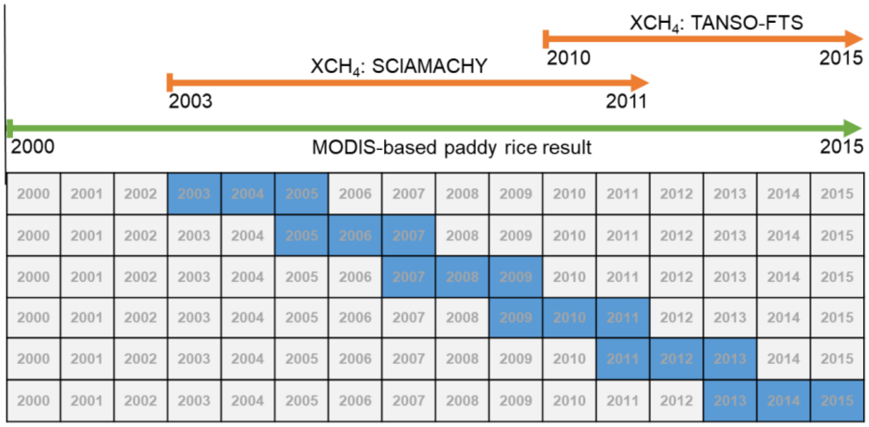

Supplementary Figure 30. Time phases of coverage for SCIAMACHY XCH_4 data, TANSO-FTS GOSAT XCH_4 data, and MODIS-based paddy rice maps. The six 3-year moving-window periods with blue color rectangle are selected to analyze the spatial and seasonal relationships between rice paddies and XCH_4 in this study.

Supplementary Figure 31

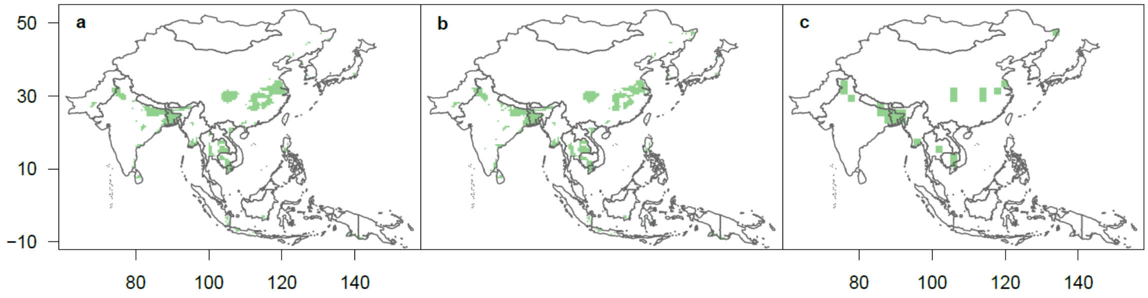

Supplementary Figure 31. Spatial distributions of MODIS-based multi-year averaged rice paddy gridcells with area proportion larger than 20% over monsoon Asia during 2003–2005 with 0.5° resolution (**a**), 2007–2009 with 0.5° resolution (**b**), and 2011–2013 with 2° resolution (**c**).

Supplementary Figure 32

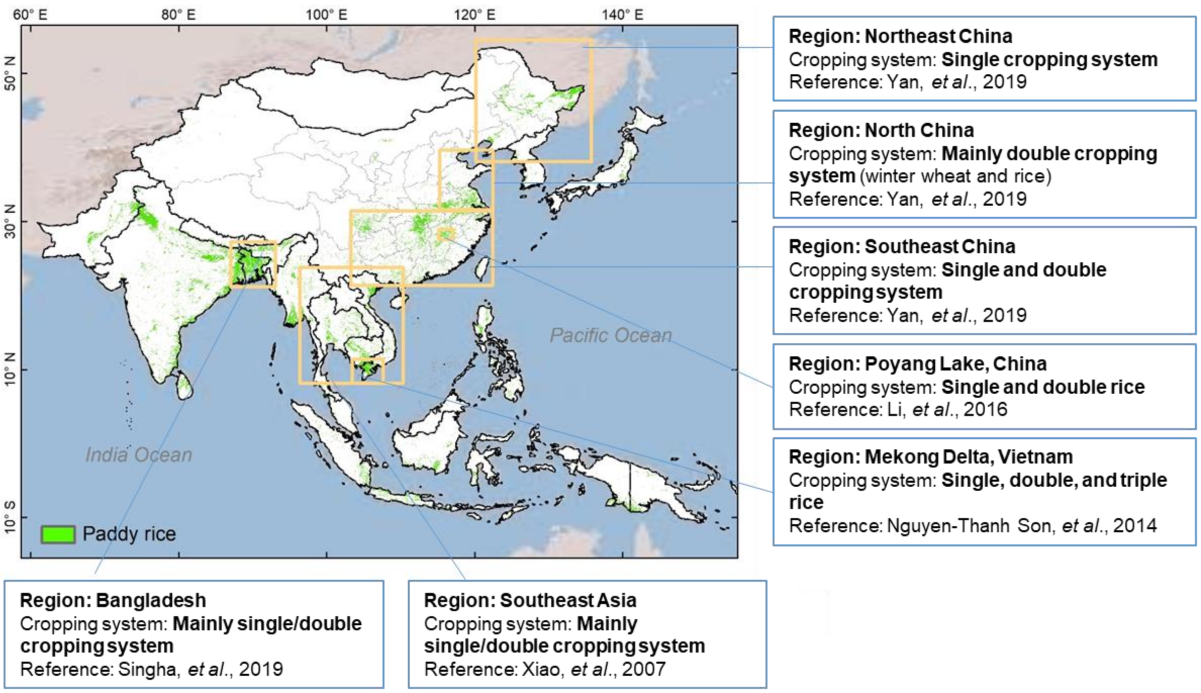

Supplementary Figure 32. Summary of cropping systems in different geographic regions based on previous studies [10, 12-15]. The map in the figure is the paddy rice map retrieved from MODIS data with 500 m resolution in monsoon Asia in 2015.

Supplementary Figure 33

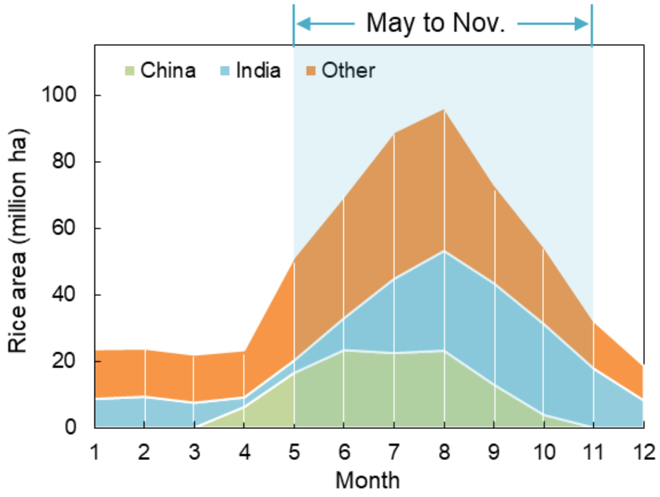

Supplementary Figure 33. Monthly rice crop area in Asia, 2010-2012. The data in the figure is from Laborte et al. [16].

Supplementary Figure 34

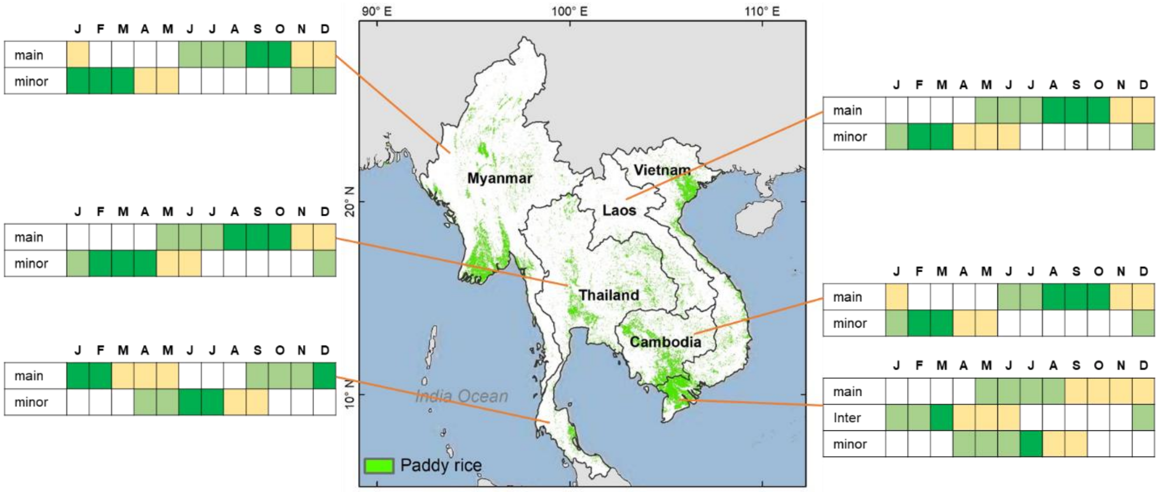

Supplementary Figure 34. Rice calendar in different geographic regions of Southeast Asia. The figure is modified from the presentation by Prof. Thuy Le Toan in the NASA LCLUC workshop 2019 (http://lcluc.umd.edu/sites/default/files/lcluc_documents/GEORICE-Thuy%20Le%20Toan.pdf). The map in the figure is the paddy rice map retrieved from MODIS data with 500 m resolution in Southeast Asia in 2015.

Supplementary Figure 35

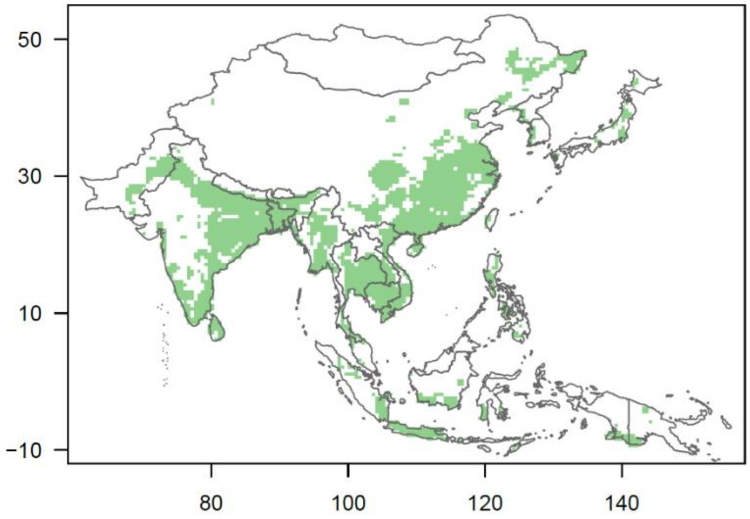

Supplementary Figure 35. Spatial distribution of MODIS-based rice paddy gridcells (0.5°×0.5°) with area proportion larger than 10%, according to the maximum spatial domain of rice paddies during 2000–2015 over monsoon Asia.

Supplementary Figure 36

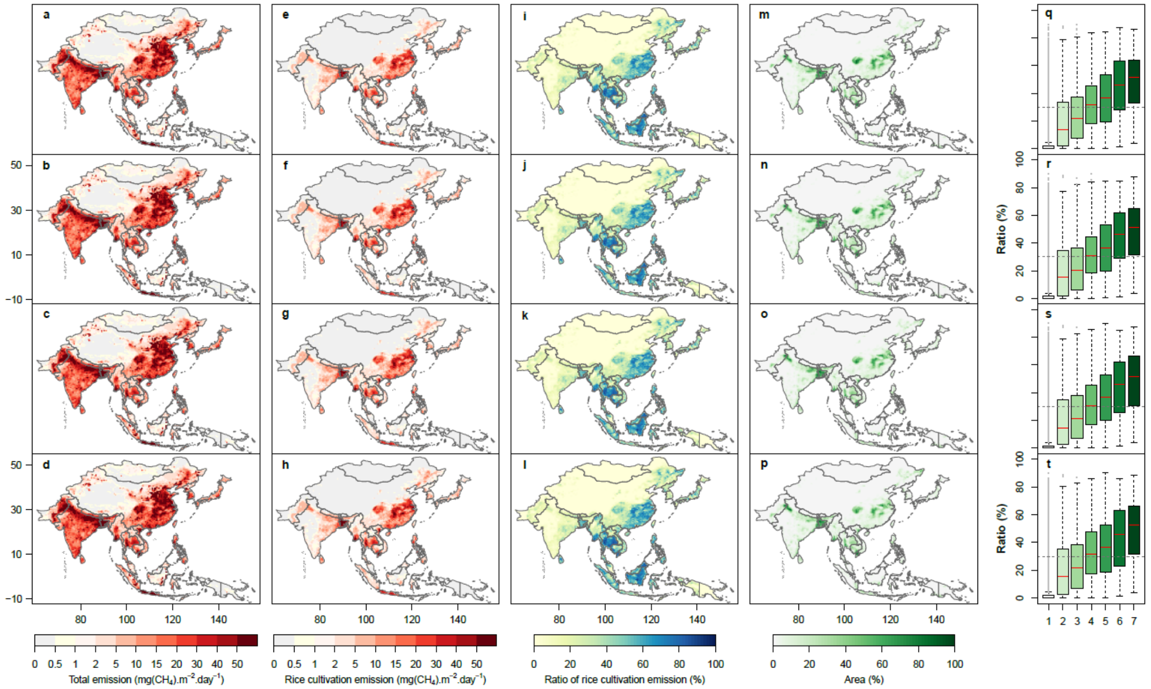

Supplementary Figure 36. Relative contribution of paddy rice cultivation CH_4 emission to the total CH_4 emission according to the Emissions Database for Global Atmospheric Research (EDGAR) CH_4 emission dataset for 3-year periods (2003-2005, 2005-2007, 2007-2009, and 2009-2011). **a-d** Spatial distributions of multi-year averaged CH_4 emission from all sectors for 2003-2005 (**a**), 2005-2007 (**b**), 2007-2009 (**c**), and 2009-2011 (**d**). **e-h** Spatial distributions of multi-year averaged paddy rice cultivation CH_4 emission for 2003-2005 (**e**), 2005-2007 (**f**), 2007-2009 (**g**), and 2009-2011 (**h**). **i-l** Spatial distributions of the ratio of paddy rice cultivation CH_4 emission to total CH_4 emission for 2003-2005 (**i**), 2005-2007 (**j**), 2007-2009 (**k**), and 2009-2011 (**l**). **m-p** Spatial distributions of rice paddy area proportions for 2003-2005 (**m**), 2005-2007 (**n**), 2007-2009 (**o**), and 2009-2011 (**p**). **q-t** Ratios of paddy rice cultivation CH_4 emission to total CH_4 emission along different paddy rice area proportion gradients for 2003-2005 (**q**),

2005-2007 (**r**), 2007-2009 (**s**), and 2009-2011 (**t**). The x-axis values in figures **q-t** represent levels of rice paddy area proportions in monsoon Asia, and 1–7 correspond to <0.5%, 0.5~1%, 1~5%, 5~10%, 10~20%, 20~40%, and >40% rice paddy area proportions, respectively. The gray dash lines in figures q-t are the horizontal lines with ratio of paddy rice cultivation CH_4 emission equal to 30%.

Supplementary Table 1. Spatial relationships between rice paddy area percentage and atmospheric methane concentration in monsoon Asia based on spatial error model (SEM) for six different 3-year periods

Period		2003–2005	2005–2007	2007–2009	2009–2011	2011–2013	2013–2015
Resolution of data		0.5 º	0.5 º	0.5 º	0.5 º	2 º	2 º
Spatial autocorrelation test of dependent	Moran's I statistic	0.83	0.71	0.69	0.68	0.61	0.53
	Moran's I statistic SD	53.08	46.47	44.23	42.86	8.67	7.76
	P-value of Moran's I test	<0.001	<0.001	<0.001	<0.001	<0.001	<0.001
Spatial error model	Intercept	1774.12	1775.80	1785.40	1806.61	1802.03	1823.99
	P-value (intercept)	<0.001	<0.001	<0.001	<0.001	<0.001	<0.001
	**Coefficient**	**11.12**	**9.54**	**10.77**	**12.77**	**61.21**	**49.56**
	**P-value (coefficient)**	**<0.001**	**<0.001**	**<0.001**	**<0.001**	**<0.001**	**0.0012**
	Adjusted R-squared	-	-	-	-	-	-
	Degrees of freedom	1775	1814	1776	1733	125	121
	AIC	12635	14213	14296	13738	1006	984
Spatial autocorrelation test of residuals	Moran's I statistic	**-0.15**	**-0.12**	**-0.10**	**-0.10**	**-0.05**	**0.02**
	Moran's I statistic SD	-9.45	-8.16	-6.62	-6.27	-0.56	0.46
	**P-value of Moran's I test**	**1.00**	**1.00**	**1.00**	**1.00**	**0.71**	**0.32**

Note: The region is the area with rice paddy area proportions larger than 10% for each period (2003–2005, 2005–2007, 2007–2009, 2009–2011, 2011–2013, and 2013–2015). SD means standard deviation.

Supplementary Table 2. Spatial relationships between rice paddy area percentage and atmospheric methane concentration in monsoon Asia but for Indonesia and Malaysia based on spatial error model (SEM). The three 3-year periods (2003–2005, 2007–2009, and 2011–2013 in Figure 2 of main text) were selected here.

Period		2003–2005	2007–2009	2011–2013
Resolution of data		0.5 º	0.5 º	2 º
Spatial autocorrelation test of dependent	Moran's I statistic	0.73	0.54	0.07
	Moran's I statistic SD	45.55	33.87	8.67
	P-value of Moran's I test	<0.001	<0.001	0.09
Spatial error model	Intercept	1780.17	1790.95	1799.57
	P-value (intercept)	<0.001	<0.001	<0.001
	**Coefficient**	**12.17**	**12.83**	**67.67**
	**P-value (coefficient)**	**<0.001**	**<0.001**	**0.0013**
	Adjusted R-squared	-	-	-
	Degrees of freedom	1614	1614	114
	AIC	11234	12785	1018
Spatial autocorrelation test of residuals	Moran's I statistic	**-0.12**	**-0.08**	**-0.006**
	Moran's I statistic SD	-7.35	-4.81	-0.23
	**P-value of Moran's I test**	**1.00**	**1.00**	**0.41**

Note: The region is the area with rice paddy area proportions larger than 10% for each period (2003–2005, 2007–2009, and 2011–2013). SD means standard deviation.

Supplementary Table 3. The confusion matrix between the MODIS-based paddy rice maps and NLCD-based paddy rice planting area maps in 2015 for two case regions

Regions	MODIS-based rice map	NLCD-based validation samples			User Acc.	Producer Acc.	Overall acc.
		Rice	Non-rice	Classif. total			
Region A	Rice	141505	18705	160210	88%	82%	91.2%
	Non-rice	31805	383175	414980	92%	95%	
	Sample total	173310	401880	575190			
Region B	Rice	30137	13075	43212	70%	73%	80.5%
	Non-rice	11243	70524	81767	86%	84%	
	Sample total	41380	83599	124979			

Note: Region A is a typical mixed region of paddy rice fields and natural wetlands in Sanjiang Plain of Northeast China; Region B is a typical mountainous region in the cross-border area among three provinces in southern China, including Hubei, Anhui, and Henan provinces. Please see the maps in Supplementary Figure 28 for details.

Supplementary Table 4. Spatial relationships between rice paddy area percentage and atmospheric methane concentration in monsoon Asia based on ordinary least squares model (OLS) for six different 3-year periods

Period		2003–2005	2005–2007	2007–2009	2009–2011	2011–2013	2013–2015
Resolution of data		0.5 º	0.5 º	0.5 º	0.5 º	2 º	2 º
Spatial autocorrelation test of dependent	Moran's I statistic	0.83	0.71	0.69	0.68	0.61	0.53
	Moran's I statistic SD	53.08	46.47	44.23	42.86	8.67	7.76
	P-value of Moran's I test	<0.001	<0.001	<0.001	<0.001	<0.001	<0.001
Ordinary least squares model	Intercept	1774.03	1773.77	1783.78	1806.92	1803.73	1824.45
	P-value (intercept)	<0.001	<0.001	<0.001	<0.001	<0.001	<0.001
	**Coefficient**	**28.80**	**28.04**	**33.25**	**29.43**	**82.82**	**56.23**
	**P-value (coefficient)**	**<0.001**	**<0.001**	**<0.001**	**<0.001**	**<0.001**	**<0.001**
	Adjusted R-squared	0.09	0.06	0.08	0.06	0.16	0.09
	Degrees of freedom	1773	1812	1774	1731	123	119
	AIC	14849	15664	15556	14965	1066	1027
Spatial autocorrelation test of residuals	Moran's I statistic	**0.80**	**0.68**	**0.65**	**0.65**	**0.57**	**0.50**
	Moran's I statistic SD	51.45	44.62	41.69	41.07	8.07	7.39
	**P-value of Moran's I test**	**<0.001**	**<0.001**	**<0.001**	**<0.001**	**<0.001**	**<0.001**

Note: The region is the area with rice paddy area proportions larger than 10% for each period (2003–2005, 2005–2007, 2007–2009, 2009–2011, 2011–2013, and 2013–2015). SD means standard deviation.

Supplementary Note 1. The role of plants in methane production and emission of rice paddy

Methanogenesis in paddy soil is mainly determined by anaerobic condition and organic matter (OM) of the flooded soils, and ultimately relies on primary production and the input of OM into soils [1]. Sources of OM may be divided into soil OM, applied OM, and rhizodeposition from current season photosynthates (Supplementary Fig. 13). The amount of soil OM is small and varies slightly in a season when compared with the other sources. Applied OM includes rice straw, stubble, and roots produced in the preceding season, organic fertilizers, and so on. The amount of applied OM varies substantially in a season, and is often large in the early growing season, and then gradually decreases over time. The rhizodeposition is mainly contributed by root exudation from living rice roots and root litter (root decay and sloughing-off cells). The amount of root exudation rises as growth of rice plants, reaches a maximum during the flowering stage with a peak in root biomass, and then decreases over time. After flowering stage, root decay maybe as a main element of rhizodeposition. Previous studies have shown that rhizodeposition from current-season photosynthesis can account for over half of the total CH_4 production [1, 17, 18], which indicates that the seasonal dynamics of rice plant growth plays a critical role in the processes of methane production [1, 19, 20].

Several recent site-based studies focused on the seasonal contribution of rice plant productivity to CH_4 emission and found a strong correlation between daily CH_4 flux and rice biomass [1, 7, 8, 19, 21-24]. It illustrated that rice growth and development (including vegetation, reproductive and ripening phases) play a critical role in the processes of methane production. That is because the growth of rice plants determines root exudates

(Supplementary Fig. 13), the important OM for CH_4 emission from rice paddies, and then further controls CH_4 emissions of rice paddies [19, 24]. Some studies also indicated that the seasonal variation of CH_4 emission could be predictable from rice production [25, 26].

Supplementary Note 2. MODIS data processing

The three Moderate Resolution Imaging Spectroradiometer (MODIS) data products, including the 8-day composite surface reflectance product (MOD09A1) [27], land surface temperature (LST) product (MYD11A2) [28], and land cover type product (MCD12Q1) [29] were used for paddy rice mapping. All the MODIS data were downloaded from the USGS EROS Data Center (https://lpdaac.usgs.gov/). The frost status and crop planting were generally determined by daily minimum temperature in the high latitude areas. The MYD11A2 product from the Aqua satellite (local time ~13:30 PM and ~01:30 AM) was used because its observations were closer to the minimum LST in comparison to the land surface temperature data from the Terra satellite (local time ~10:30 AM and ~22:30 PM). Our MODIS processing procedures included three components: (1) calculating vegetation indices (VIs), (2) removing bad observations, and (3) gap-filling LST data [30, 31].

Three spectral indices were calculated using the MOD09A1 products, including enhanced vegetation index (EVI), land surface water index (LSWI), and normalized difference snow index (NDSI). Both normalized difference vegetation index (NDVI) and EVI are related to the vegetation canopy greenness. EVI is considered to have higher robustness to atmospheric conditions and soil background relative to NDVI [32-34], while LSWI is sensitive to leaf and soil water [35-37]. NDSI is widely used for snow detection [38, 39].

$$LSWI = \frac{\rho_{nir} - \rho_{swir}}{\rho_{nir} + \rho_{swir}} \quad (1)$$

$$EVI = 2.5 \times \frac{\rho_{nir} - \rho_{red}}{\rho_{nir} + 6 \times \rho_{red} - 7.5 \times \rho_{blue} + 1} \quad (2)$$

$$NDSI = \frac{\rho_{green} - \rho_{nir}}{\rho_{green} + \rho_{nir}} \quad (3)$$

where ρ_{blue}, ρ_{green}, ρ_{red}, ρ_{nir}, and ρ_{swir} are the reflectance for the blue, green, red, Near-Infrared (NIR), and Short-Wave Infrared (SWIR1) bands, respectively.

For the MOD09A1 product, we identified bad observations (clouds, cloud shadows, and snow) in two steps. First, the quality control flag layer was used to extract the clouds and cloud shadows from each observation. Second, we further recognized clouds by using the blue reflectance of ≥ 0.2 [40, 41]. The snow cover observations were also identified and excluded by using NDSI and the NIR band (NDSI > 0.40 and NIR > 0.11) [38, 39]. All the observations identified as clouds, cloud shadows, or snow cover were excluded. Next, we filled the gaps in the time series LST data by using the linear interpolation approach [42]. The 8-day smoothed LST product was used to determine the thermal growing season of vegetation, which was used to identify the temperature-based time window for rice transplanting. The first and last dates of stable minimum temperatures higher than 5°C in continuous three 8-day intervals were identified as the start and end of thermal growing season (Supplementary Fig. 21). The resultant maps of the start and end dates of thermal growing season were resampled to 500 m using the nearest neighbor method to match the vegetation index data. The resultant maps for 2003 were used to extract rice paddies during 2000–2002 due to the unavailability of MYD11A2 during these three years.

Supplementary Note 3. Mapping rice paddies with time series MODIS data

The EVI and LSWI within the time window of flooding and transplanting were used to identify the observations with signals of flooding and transplanting, and a pixel was assumed to be a "potential or likely" rice paddy if one or more observations were identified in that manner (Eq. (4)–(7)).

$$F_{Ti} = \begin{cases} 1 & (LSWI_{Ti} + 0.05 \geq EVI_{Ti}) \\ 0 & (LSWI_{Ti} + 0.05 < EVI_{Ti}) \end{cases} \tag{4}$$

$$Rice_p = Max(F_{T1}, F_{T2}, \ldots, F_{Tn})(SOF \leq T \leq EOF) \tag{5}$$

$$SOF = DOY\left(start\,date\,of\ LST_{night} \geq 5°C\right) \tag{6}$$

$$EOF = \begin{cases} DOY\left(start\,date\,of\ LST_{night} \geq 5°C\right) + 80 & (Northern\ region) \\ DOY\left(end\,date\,of\ LST_{night} \geq 5°C\right) & (Southern\ region) \end{cases} \tag{7}$$

where Ti is the ith 8-day composite period between start date of flooding (SOF) and end date of flooding (EOF), F_{Ti} is the flooding status at Ti between the SOF and EOF, $Rice_p$ is potential rice paddy, DOY is the day of year, and LST_{night} is nighttime LST. Northern region is the region including Mongolia, four provinces in China (Inner Mongolia, Heilongjiang, Jilin, and Liaoning), North Korea, South Korea, and Japan. Southern region is the region except northern region in monsoon Asia.

Paddy rice is an unique crop type which needs to be transplanted on the flooding fields [43], so paddy flooding is characteristic of paddy rice agriculture. However, some other land covers could also be flooded, such as permanent water bodies. Other land cover could occasionally flood due to spring flooding or precipitation events. Therefore, we developed

non-cropland masks to minimize commission errors to the largest possible degree, which included evergreen vegetation, deciduous vegetation, sparsely vegetated, natural wetlands, topographic masks, and temperature-based mask.

The masks were produced using the following methods. First, we generated maps of evergreen vegetation using LSWI time series data [40, 44, 45]. If the LSWI value of a pixel was larger or equal to 0.15 in all good observations in one year, it was labeled as evergreen vegetation. We generated annual evergreen vegetation maps, and then a 16-year evergreen vegetation frequency map. To reduce the error due to effects of data quality on evergreen vegetation mapping, evergreen vegetation pixels were classified as those that were detected as evergreen at least eight times in 16 years (2000–2015, 50% detection rate) (Supplementary Fig. 22a). Second, we used the forest mask derived from the analysis of the Phased Array type L-band Synthetic Aperture Radar (PALSAR) data in 2010 at 50 m spatial resolution [46]. It was aggregated to 500 m to be spatially consistent with the MODIS data and a 70% threshold was used to extract forest (Supplementary Fig. 22b). Third, we generated maps of sparsely vegetated land (e.g., saline and alkaline land, build-up) using annual maximum EVI less than 0.4 as a threshold [31], and permanent water body was also included in this mask (Supplementary Fig. 22c). Fourth, we used two available maps of natural wetlands: the National Land Cover and Land Use Dataset (NLCD)-based wetland map in 2010 in China [47] (Supplementary Fig. 22d) and the International Geosphere-Biosphere Programme (IGBP)-based wetland map from MOD12Q1 during 2001–2013 (Supplementary Fig. 22e). Fifth, we generated topographic masks to remove regions above 2600 m above sea level (*asl.*) [48] and with a slope greater than 4° where it was unsuitable for paddy rice planting, by using Shuttle Radar Topography Mission (SRTM) 90 m Digital

Elevation Model (DEM) data (Supplementary Fig. 22f, g). Last, the length of LST >= 5 °C less than 100 days was used as an annual temperature-based mask throughout the study area (Supplementary Fig. 22h), which can help remove the noises occurring in cold region or montane areas. We first generated annual paddy rice maps in monsoon Asia during 2000-2015 by excluding these masks from annual potential rice paddy (flooding and transplanting) layers.

Supplementary Note 4. Accuracy assessment of MODIS-based paddy rice maps

The accuracy assessment of land cover maps is a critical component of land cover mapping. To assess the accuracy of annual paddy rice maps in monsoon Asia, we have conducted ground truth data based validation in different study areas by using ground survey data or crowd-sourced field photo data in high latitude regions of Asia [49]. Given the huge land area in this region, it was challenging and beyond our capacity to collect national-level ground survey data for monsoon Asia. Therefore, in this paper, we focused on the comparisons with the existing paddy rice products.

We conducted comparisons with a set of existing national paddy rice maps and statistical data. The paddy rice maps included: 1) the paddy rice map of South Asia during 2000-2001 with 500 m resolution from the International Rice Research Institute (IRRI) [50], which had 12 rice classes, including irrigated and rain-fed rice. The overall accuracy was 80 % for all classes. The irrigated rice layer in the map was used for comparison in this study for South Asia; 2) the paddy rice map of Southeast Asia from a map of lowland rice extent in the major rice planting countries of Asia with 500 m resolution [48], which was a hybrid map by integrating annual paddy rice maps from 2000 to 2012 with over three times of paddy rice occurrence frequency; 3) the RapidEye-based paddy rice map in North Korea

in 2010 with 5-6.5m resolution [11]; 4) the OECD-derived paddy rice map in South Korea in 2008 with 30m resolution (available from http://egis.me.go.kr/atlas/list.do); 5) the AVNIR-2-based rice paddy in Japan around 2009 with 30m resolution [51]; and 6) NLCD-based paddy rice maps in China in *ca*. 2000, 2005, 2008, and 2010 derived from visual interpretation of Landsat images [52-54]. The NLCD-based land use fractional data with 1km resolution was used for area comparisons at provincial and prefectural levels, also the 100-m raster layer was used for pixel-to-pixel comparisons [55]. The statistical data was from the FAO Statistical Databases (FAOSTAT) by using the indicator of paddy rice harvested area in each country of monsoon Asia from 2000 to 2015. The FAOSTAT data was downloaded from the official website (http://www.fao.org/faostat/en/).

1) Accuracy assessment of paddy rice maps in China

The NLCD datasets agreed well with the MODIS-based paddy rice maps in China. The spatial patterns of MODIS-based rice paddies were consistent with those of the NLCD-based rice paddy fractional layers in all the four periods (2000, 2005, 2008, and 2010), particularly for the pixels with dense rice paddy fractions over 40% (Supplementary Fig. 23). Statistical analyses at both provincial and prefectural levels showed significant correlations between the MODIS-based and NLCD-based maps (R^2 ranging from 0.72-0.89, Supplementary Fig. 23). All the slopes of linear regressions were close to 1.

2) Accuracy assessment of paddy rice maps in Korea Peninsular and Japan

The existing local paddy rice maps were used for the comparison of MODIS-based paddy rice maps in northeastern Asia, including the RapidEye-based rice paddy map in North Korea in 2010 [11], the OECD-derived paddy rice map in South Korea in 2008, and the AVNIR-2-based rice paddy map in Japan around 2009 [51]. The comparisons showed

that the MODIS-based paddy rice map in 2010 coincided spatially with the 5-6.5m RapidEye-based paddy rice map in North Korea (Supplementary Fig. 24a-d), even in the eastern and southern regions where small rice fields distributed sparsely [11]. The statistical analysis at provincial level also showed a significant correlation between the MODIS-based and RapidEye-based paddy rice maps (R^2=0.95, P<0.01, Supplementary Fig. 24m). In South Korea, the resultant paddy rice map agreed with the OECD-derived paddy rice map in 2008 (Supplementary Fig. 24e-h). The area statistics at provincial level was also significantly correlated with that of the MODIS-based paddy rice map (R^2=0.95, P<0.01, Supplementary Fig. 24n). In Japan, the AVNIR-2-based paddy rice map showed a consistency with the MODIS-based paddy rice map (Supplementary Fig. 24i-l). The AVNIR-2-based rice paddy area statistics at provincial level was significantly correlated with that of the MODIS-based paddy rice map (R^2=0.80, P<0.01, Supplementary Fig. 24o).

3) Accuracy assessment of paddy rice map in South Asia

The IRRI-based paddy rice map during 2000–2001 was used to compare with the MODIS-based paddy rice map in the same period in South Asia, mainly including India, Bangladesh, Pakistan, and Nepal. Both the maps showed very consistent spatial patterns (Supplementary Fig. 25). The comparisons between the MODIS-based and IRRI-based paddy rice maps at provincial and prefectural levels in South Asia during 2000–2001 showed significant correlations at both levels (R^2 = 0.92 for provincial comparison, R^2 = 0.81 for prefectural comparison, P<0.01, Supplementary Fig. 25g, h).

4) Accuracy assessment of paddy rice map in Southeast Asia

The nine countries in Southeast Asia with dense paddy rice areas were considered here, including: Cambodia, Laos, Myanmar, Thailand, Vietnam, Malaysia, Brunei, Indonesia,

and Philippines. We generated a baseline paddy rice map with over three times of rice paddy occurrence frequency during 2000–2012, to match the IRRI-based paddy rice baseline map. The results showed a consistency between the two maps (Supplementary Fig. 26). Despite an overestimate comparing to the IRRI-based paddy rice map, the statistical comparison between MODIS-based and IRRI-based paddy rice at provincial level in Southeast Asia during 2000–2012 showed a significant correlation (R^2 = 0.86, P<0.01, Supplementary Fig. 26g).

Furthermore, we compared the MODIS-based rice paddy area with the statistical data of FAOSTAT at national scale (Supplementary Fig. 27). These comparisons showed high consistencies between them for each year with R^2 ranging from 0.82-0.95. We also validated the maps based on pixel-level comparison using higher resolution Landsat-based paddy rice maps in two sample regions of China based on the 100-m NLCD: 1) one typical mixed region of paddy rice fields and natural wetlands in Sanjiang Plain of Northeast China, 2) one typical mountainous region in the cross-border area among three provinces in southern China including Hubei, Anhui, and Henan provinces (Supplementary Fig. 28). The overall accuracies of the paddy rice maps for the two regions are 91% and 81% (Supplementary Table S3). The high consistency of spatial distributions between MODIS-based paddy rice maps and these existing national paddy rice maps, as well as the high correlations between MODIS-based results and the statistical data in these countries, showed that our MODIS-based paddy rice maps had a reasonably high accuracy, and can examine inter-annual variations of rice paddy areas. Besides the aforementioned validations and comparisons, we will release the data together with the publication and expect a crowd-sourcing validation from the users.

Supplementary References

1. Hayashi K, et al. Cropland soil-plant systems control production and consumption of methane and nitrous oxide and their emissions to the atmosphere. *Soil Sci Plant Nutr* **61**, 2-33 (2015).
2. McMillan AMS, Goulden ML, Tyler SC. Stoichiometry of CH4 and CO2 flux in a California rice paddy. *J Geophys Res-Biogeo* **112**, G01008 (2007).
3. Meijide A, et al. Seasonal trends and environmental controls of methane emissions in a rice paddy field in Northern Italy. *Biogeosciences* **8**, 3809-3821 (2011).
4. Bhattacharyya P, et al. Tropical low land rice ecosystem is a net carbon sink. *Agr Ecosyst Environ* **189**, 127-135 (2014).
5. Zhang Y, et al. Simulation and estimation of methane emissions from rice paddies in Sanjiang Plain of the Northeast China. *Trans Chin Soc Agric Eng*, 293-298 (2011).
6. Ge H-X, et al. The characteristics of methane flux from an irrigated rice farm in East China measured using the eddy covariance method. *Agricultural and Forest Meteorology* **249**, 228-238 (2018).
7. Knox SH, et al. Biophysical controls on interannual variability in ecosystem-scale CO2 and CH4 exchange in a California rice paddy. *Journal of Geophysical Research-Biogeosciences* **121**, 978-1001 (2016).
8. Alberto MCR, et al. Measuring methane flux from irrigated rice fields by eddy covariance method using open-path gas analyzer. *Field Crop Res* **160**, 12-21 (2014).
9. Zhao M, et al. Simulation of greenhouse gas effluxes in rice fields based on DNDC model. *Chinese Journal of Ecology*, 1057-1066 (2019).
10. Son N-T, et al. A Phenology-Based Classification of Time-Series MODIS Data for Rice Crop Monitoring in Mekong Delta, Vietnam. *Remote Sensing* **6**, 135-156 (2013).
11. Hong S, et al. Estimation of Paddy Field Area in North Korea Using RapidEye Images. *Korean Journal of Soil Science & Fertilizer* **45**, 1194-1202 (2012).
12. Xiao XM, et al. Remote sensing, ecological variables, and wild bird migration related to outbreaks of highly pathogenic H5N1 avian influenza. *J Wildl Dis* **43**, S40-S46 (2007).
13. Li P, et al. Mapping rice cropping systems using Landsat-derived Renormalized Index of Normalized Difference Vegetation Index (RNDVI) in the Poyang Lake Region, China. *Frontiers of Earth Science* **10**, 303-314 (2016).
14. Singha M, Dong J, Zhang G, Xiao X. High resolution paddy rice maps in cloud-prone Bangladesh and Northeast India using Sentinel-1 data. *Scientific Data* **6**, 26 (2019).

15. Yan H, et al. Tracking the spatio-temporal change of cropping intensity in China during 2000–2015. *Environmental Research Letters* **14**, 035008 (2019).
16. Laborte AG, et al. RiceAtlas, a spatial database of global rice calendars and production. *Scientific Data* **4**, 170074 (2017).
17. Watanabe A, Takeda T, Kimura M. Evaluation of origins of CH4 carbon emitted from rice paddies. *J Geophys Res-Atmos* **104**, 23623-23629 (1999).
18. Tokida T, et al. Methane and soil CO2 production from current-season photosynthates in a rice paddy exposed to elevated CO2 concentration and soil temperature. *Global Change Biol* **17**, 3327-3337 (2011).
19. Huang Y, Sass RL, Fisher FM. Methane emission from Texas rice paddy soils. 2. Seasonal contribution of rice biomass production to CH4 emission. *Global Change Biol* **3**, 491-500 (1997).
20. Schutz H, Schroder P, Rennenberg H. Role of plants in regulating the methane flux to the atmosphere. In: Trace Gas Emissions by Plants. In: *Trace Gas Emissions by Plants* (ed^(eds Sharkey T, Holland E, Mooney H). Academic Press Inc (1991).
21. Sass RL, Fisher FM, Harcombe PA, Turner FT. Methane Production and Emission in a Texas Rice Field. *Global Biogeochem Cy* **4**, 47-68 (1990).
22. Huang Y, et al. Comparison of field measurements of CH4 emission from rice cultivation in Nanjing, China and in Texas, USA. *Adv Atmos Sci* **18**, 1121-1130 (2001).
23. Gogoi N, Baruah K, Gogoi B, Gupta PK. Methane Emission from Two Different Rice Ecosystems (Ahu and Sali) at Lower Brahmaputra Valley Zone of North East India. *Appl Ecol Env Res* **6**, 99-112 (2008).
24. Neue HU, et al. Factors and processes controlling methane emissions from rice fields. *Nutrient Cycling in Agroecosystems* **49**, 111-117 (1997).
25. Huang Y, Sass RL, Fisher FM. A semi-empirical model of methane emission from flooded rice paddy soils. *Global Change Biol* **4**, 247-268 (1998).
26. Fumoto T, et al. Revising a process-based biogeochemistry model (DNDC) to simulate methane emission from rice paddy fields under various residue management and fertilizer regimes. *Global Change Biol* **14**, 382-402 (2008).
27. Vermote E, Vermeulen A. Atmospheric correction algorithm: spectral reflectances (MOD09). *ATBD version* **4**, (1999).
28. Wan ZM. New refinements and validation of the MODIS Land-Surface Temperature/Emissivity products. *Remote Sens Environ* **112**, 59-74 (2008).

29. Friedl MA, et al. Global land cover mapping from MODIS: algorithms and early results. *Remote Sens Environ* **83**, 287-302 (2002).
30. Zhang G, et al. Mapping paddy rice planting areas through time series analysis of MODIS land surface temperature and vegetation index data. *ISPRS Journal of Photogrammetry and Remote Sensing* **106**, 157-171 (2015).
31. Zhang G, et al. Spatiotemporal patterns of paddy rice croplands in China and India from 2000 to 2015. *Sci Total Environ* **579**, 82-92 (2017).
32. Huete AR, Liu HQ, Batchily K, vanLeeuwen W. A comparison of vegetation indices global set of TM images for EOS-MODIS. *Remote Sens Environ* **59**, 440-451 (1997).
33. Huete A, et al. Overview of the radiometric and biophysical performance of the MODIS vegetation indices. *Remote Sens Environ* **83**, 195-213 (2002).
34. Xiao X, et al. Sensitivity of vegetation indices to atmospheric aerosols: continental-scale observations in Northern Asia. *Remote Sensing of Environment* **84**, 385-392 (2003).
35. Xiao X, et al. Quantitative relationships between field-measured leaf area index and vegetation index derived from VEGETATION images for paddy rice fields. *Int J Remote Sens* **23**, 3595-3604 (2002).
36. Maki M, Ishiahra M, Tamura M. Estimation of leaf water status to monitor the risk of forest fires by using remotely sensed data. *Remote Sens Environ* **90**, 441-450 (2004).
37. Xiao X, et al. Observation of flooding and rice transplanting of paddy rice fields at the site to landscape scales in China using VEGETATION sensor data. *Int J Remote Sens* **23**, 3009-3022 (2002).
38. Hall DK, Riggs GA, Salomonson VV. Development of Methods for Mapping Global Snow Cover Using Moderate Resolution Imaging Spectroradiometer Data. *Remote Sens Environ* **54**, 127-140 (1995).
39. Hall DK, et al. MODIS snow-cover products. *Remote Sens Environ* **83**, 181-194 (2002).
40. Xiao X, et al. Mapping paddy rice agriculture in southern China using multi-temporal MODIS images. *Remote Sensing of Environment* **95**, 480-492 (2005).
41. Xiao X, et al. Mapping paddy rice agriculture in South and Southeast Asia using multi-temporal MODIS images. *Remote Sens Environ* **100**, 95-113 (2006).
42. Pan Z, et al. Mapping crop phenology using NDVI time-series derived from HJ-1 A/B data. *International Journal of Applied Earth Observation and Geoinformation* **34**, 188-197 (2015).
43. LeToan T, et al. Rice crop mapping and monitoring using ERS-1 data based on experiment and modeling results. *Ieee T Geosci Remote* **35**, 41-56 (1997).

44. Xiao X, et al. A Simple Algorithm for Large-Scale Mapping of Evergreen Forests in Tropical America, Africa and Asia. *Remote Sensing* **1**, 355-374 (2009).
45. Xiao X, et al. Characterization of forest types in Northeastern China, using multi-temporal SPOT-4 VEGETATION sensor data. *Remote Sensing of Environment* **82**, 335-348 (2002).
46. Qin Y, et al. Mapping forests in monsoon Asia with ALOS PALSAR 50-m mosaic images and MODIS imagery in 2010. *Sci Rep* **6**, 20880 (2016).
47. Liu J, et al. Spatiotemporal characteristics, patterns, and causes of land-use changes in China since the late 1980s. *Journal of Geographical Sciences* **24**, 195-210 (2014).
48. Nelson A, Gumma MK. A map of lowland rice extent in the major rice growing countries of Asia. (ed^(eds IRRI). IRRI (2015).
49. Dong J, et al. Northward expansion of paddy rice in northeastern Asia during 2000-2014. *Geophys Res Lett* **43**, 3754-3761 (2016).
50. Gumma MK, Nelson A, Thenkabail PS, Singh AN. Mapping rice areas of South Asia using MODIS multitemporal data. *Journal of Applied Remote Sensing* **5**, 053547 (2011).
51. JAXA-EORC. High-Resolution Land Use and Land Cover Map in Japan during 2006~2011. (ed^(eds). JAXA (2012).
52. Liu J, et al. Spatial and temporal patterns of China's cropland during 1990–2000: An analysis based on Landsat TM data. *Remote Sens Environ* **98**, 442-456 (2005).
53. Liu JY, et al. Spatial patterns and driving forces of land use change in China during the early 21st century. *Journal of Geographical Sciences* **20**, 483-494 (2010).
54. Liu Z, et al. Change analysis of rice area and production in China during the past three decades. *Journal of Geographical Sciences* **23**, 1005-1018 (2013).
55. Felipe-Lucia MR, et al. Multiple forest attributes underpin the supply of multiple ecosystem services. *Nat Commun* **9**, 4839 (2018).
